# Modular Bioorthogonal Lipid Nanoparticle Modification Platforms for Cardiac Homing

**DOI:** 10.1021/jacs.3c07811

**Published:** 2023-10-09

**Authors:** Raquel Cruz-Samperio, Corrigan L. Hicks, Aaron Scott, Ignacio Gispert Contamina, Yuval Elani, Rebecca J. Richardson, Adam W. Perriman

**Affiliations:** † School of Cellular and Molecular Medicine, University of Bristol, Biomedical Sciences Building, University Walk, Bristol BS8 1TD, U.K.; ‡ School of Physiology, Pharmacology and Neuroscience, University of Bristol, Biomedical Sciences Building, University Walk, Bristol BS8 1TD, U.K.; § Department of Chemical Engineering, Imperial College London, South Kensington, London SW7 2AZ, U.K.; ∥ Research School of Chemistry, Australian National University, Canberra ACT 2601, Australia; ⊥ John Curtin School of Medical Research, Australian National University, Canberra ACT 2601, Australia

## Abstract

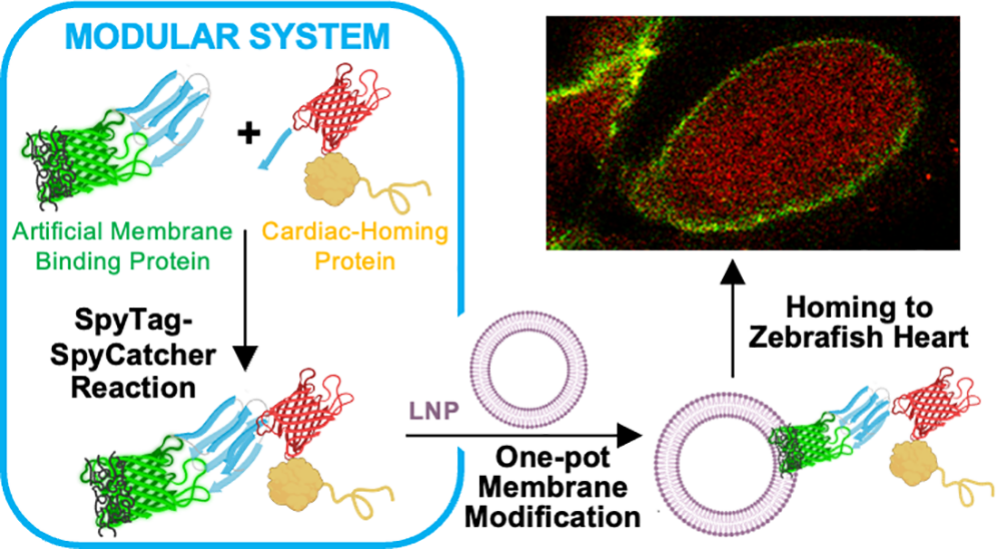

Lipid nanoparticles (LNPs) are becoming widely adopted as vectors for the delivery of therapeutic payloads but generally lack intrinsic tissue-homing properties. These extracellular vesicle (EV) mimetics can be targeted toward the liver, lung, or spleen via charge modification of their lipid headgroups. Homing to other tissues has only been achieved via covalent surface modification strategies using small-molecule ligands, peptides, or monoclonal antibodies—methods that are challenging to couple with large-scale manufacturing. Herein, we design a novel modular artificial membrane-binding protein (AMBP) platform for the modification of LNPs postformation. The system is composed of two protein modules that can be readily coupled using bioorthogonal chemistry to yield the AMBP. The first is a membrane anchor module comprising a supercharged green fluorescent protein (scGFP) electrostatically conjugated to a dynamic polymer surfactant corona. The second is a functional module containing a cardiac tissue fibronectin homing sequence from the bacterial adhesin CshA. We demonstrate that LNPs modified using the AMBP exhibit a 20-fold increase in uptake by fibronectin-rich C2C12 cells under static conditions and a 10-fold increase under physiologically relevant shear stresses, with no loss of cell viability. Moreover, we show targeted localization of the AMBP-modified LNPs in zebrafish hearts, highlighting their therapeutic potential as a vector for the treatment of cardiac disease and, more generally, as a smart vector.

## Introduction

Lipid nanoparticles (LNPs) are stable, spherical structures formed via the self-assembly of ionizable lipids. They have recently been in the spotlight for their role as RNA nanocarriers in clinical applications,^1^ and recent examples include the BioNTech/Pfizer’s BNT162b2 and Moderna’s mRNA-1273 SARS-CoV-2 vaccines. They have also been investigated as extracellular vesicle (EV) mimetics, which have been shown to play important roles in tissue repair via paracrine signaling^2^ after myocardial infarction (MI). EVs have been shown to induce angiogenesis,^3^ reduce fibrosis,^4^ and improve overall the contractile capacity of the heart in ischemic animal models due to their RNA and protein cargoes.^5^ However, clinical translation of EVs is challenging and is hampered by a lack of scalability, heterogenicity in both particle size (ranging from 30 nm up to 2 μm diameter) and cargo (proteins, enzymes, oligonucleotides, etc.), and poor in vivo pharmacokinetics.^6^ LNPs are a desirable alternative to EVs, as they are monodisperse and abundant due to scalable manufacturing processes. Moreover, there are now examples of LNPs carrying therapeutic payloads that improve angiogenesis^7^ and reduce scar size^8^ in MI animal models. Nonetheless, when delivered systemically, LNPs generally lack tissue specificity, which reduces the efficacy of targeted therapeutics. Accordingly, there have been recent efforts to improve LNP homing using engineered lipid mimetics, which have achieved improved specificity to the liver, the lung, or the spleen.^9−11^ However, systematic delivery of LNPs targeting the heart^12^ still exhibits off-target effects and has been found in other organs. Other approaches rely on covalently conjugating cell-targeting antibodies^13^ or cardiac-homing peptides^14^ to EV membrane proteins. Despite their promise, these examples generally rely on the presence of proteins on the EV membranes for chemical conjugation, which is not directly compatible with synthetic LNPs unless chemically modified lipids are used,^15^ or additional chemical steps are added to LNP manufacturing.^16^ These approaches commonly involve unstable chemical intermediates, increasing the cost and the length of synthesis and decreasing the final yield. Hence, a one-pot facile method to modify LNP membranes postformation would help drive their clinical translation.

We have recently developed an artificial membrane-binding protein (AMBP) methodology to modify the plasma membrane of cells and demonstrated the improved performance of augmented human mesenchymal stem cells (hMSCs) in regenerative medicine. The properties provided by the AMBPs to the hMSCs include responsive oxygenation,^17^ extracellular matrix (ECM) targeting^18,19^ for cartilage tissue repair, improving cell adhesion to three-dimensional (3D) scaffolds,^20^ synthetic ECM production,^21^ and cardiac homing.^22^ The AMBPs are polymer–surfactant-conjugated proteins, which are stabilized via electrostatic interactions between anionic surfactant headgroups and cationic side chains on supercharged proteins. When in the vicinity of a cell, the polymer–surfactant corona reconfigures to insert the hydrophobic tails into the lipid bilayer regions of the plasma membrane, thereby anchoring the AMBP to the cell.

**Figure 1:**
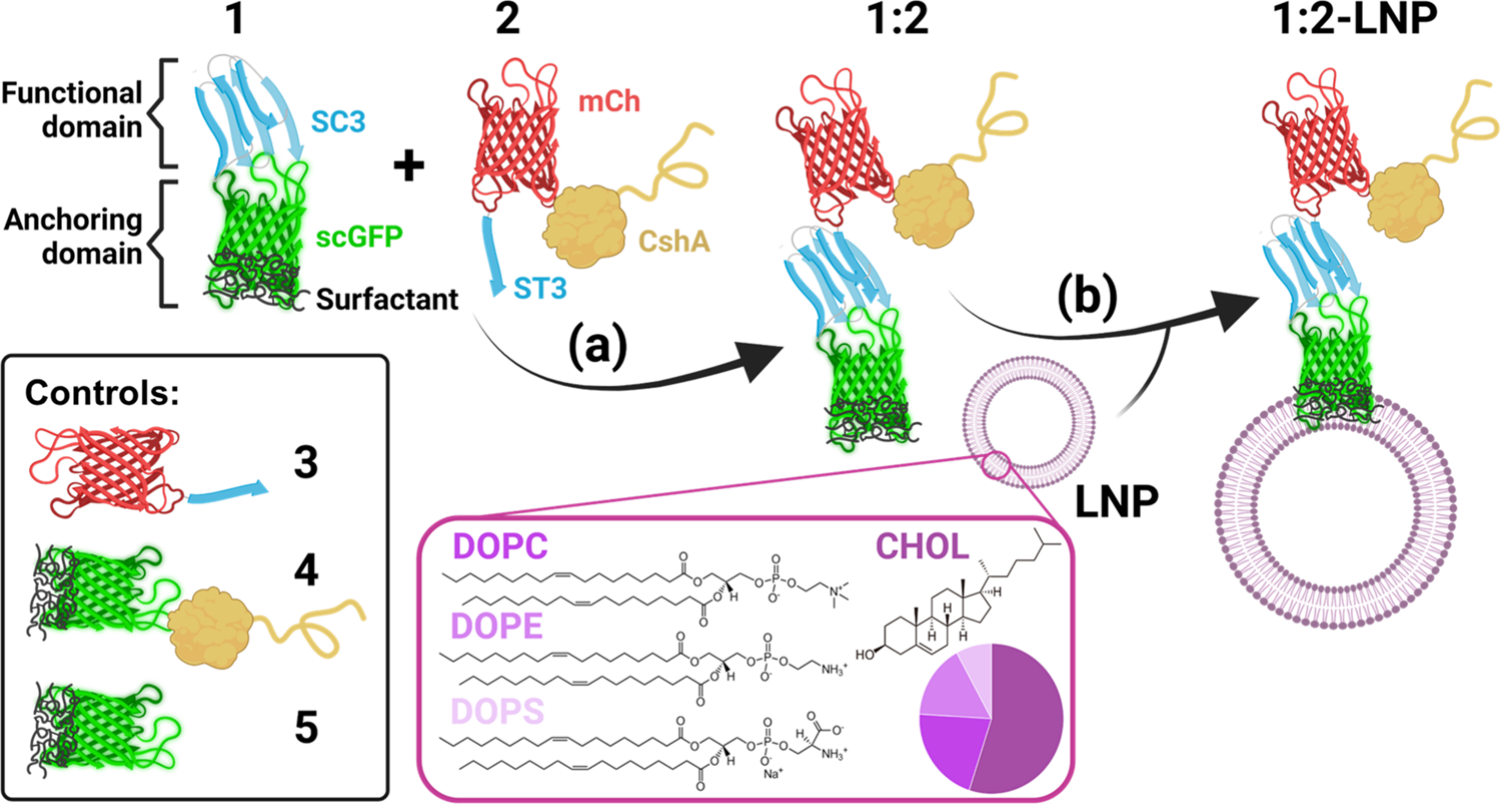
Synthesis of cardiac-homing lipid nanoparticles. (a) Reaction between protein reactive pairs to generate a modular artificial membrane-binding protein (AMBP). Protein fusion **1**, consisting of SpyCatcher (SC3, shown in blue) and supercharged green fluorescent protein (scGFP, shown in green), reacts bioorthogonally with protein fusion **2**, consisting of SpyTag (ST3, shown in blue), mCherry (mCh, shown in red), and the fibronectin-binding protein CshA (shown in yellow), forming an isopeptide bond through the side chains of SC3 Lys28 and ST3 Asp10. An additional protein fusion **3**, consisting of mCh and ST3 was generated as a negative control of cardiac homing. Other negative controls were protein fusions **4**, consisting of cardiac-homing protein CshA fused to the scGFP-anchoring domain, and **5**, consisting of just the anchoring domain. (b) Cardiac-homing (CH) lipid nanoparticle (LNP), **1:2**-LNP, is ensembled upon mixing of the modular AMBP and our in-house LNPs made of cholesterol (CHOL, 58% v/v), 2-dioleoyl-*sn*-glycero-3-phosphocholine (DOPC, 22% v/v), 1,2-dioleoyl-*sn*-glycero-3-phosphoethanolamine (DOPE, 16% v/v), and 1,2-dioleoyl-*sn*-glycero-3-phospho-l-serine (DOPS, 8% v/v). The figure was created using BioRender.com.

To date, our AMBPs comprised a single polypeptide chain, which presents challenges in expression of both high-molecular-weight proteins and undesirable polymer surfactant conjugation on key regions of functional domains. Accordingly, the prospect of an optimized universal membrane anchor is attractive. Herein, we develop a modular AMBP platform using the SpyCatcher-SpyTag^23−25^ technology (Figure 1). Protein SpyCatcher (SC) and peptide SpyTag (ST) are the result of cleaving and bioengineering the fibronectin (Fn)-binding CnaB2 domain of the FbaB protein from *Streptococcus pyogenes*, which can then react bioorthogonally, and irreversibly forming an intramolecular isopeptide bond.^23^ Since inception, both reacting partners have undergone two subsequent rounds of engineering to improve their reaction and kinetic yield,^25^ and the third iteration of the system is used in this work (SC3 and ST3). Here, SC3 is expressed as a fusion with a supercharged green fluorescent protein (scGFP), and surfactant-conjugated to produce an anchor module for the membrane of LNPs. Coupling partner ST3 is fused to red fluorescent reporter mCherry and bacterial adhesin protein CshA (*Streptococcus gordonii*),^22,26^ and bioorthogonal coupling is demonstrated. Significantly, we show that LNPs modified using the modular AMBP exhibit 20- and 10-fold increases in uptake by fibronectin-rich C2C12 myoblasts under static and dynamic conditions, respectively. Moreover, in vivo experiments performed using a zebrafish model show the homing of the modified LNPs in zebrafish hearts, signifying their potential as vectors for the treatment of cardiac diseases.

## Results and Discussion

### Synthesis and Characterization of Protein Reactive Pairs

Three gene constructs were designed to encode protein fusion **1**, comprising membrane anchor domain scGFP fused to the SC3 functional domain; protein fusion **2**, consisting of fibronectin-binding protein CshA fused to mCherry with ST3 on the C-terminus, and protein fusion **3**, consisting of mCherry fused to ST3, which was used as a negative control (scheme of genes can be found in Figure S1a and sequences are detailed in Table S1). Plasmids encoding protein fusion **4** containing scGFP fused to CshA and protein **5** consisting of scGFP were available from previous work, and these AMBPs were produced using methods described previously.^18,22^ Genes encoding proteins **1**, **2**, and **3** were cloned into pET45b vectors (primer sequences are detailed in Table S2), expressed in *Escherichia coli* BL21 (DE3), and purified by nickel affinity chromatography (IMAC) and size-exclusion chromatography (SEC), as detailed in the Supporting Information (SI), and protein purity was determined by sodium dodecyl sulfate-polyacrylamide gel (SDS-PAGE) (Figure S1). A tobacco-etch virus (TEV) cleavage sequence was incorporated in the N-termini of SC3 and CshA to remove the polyhistidine tag (His_6*x*_) to avoid interference in bioorthogonal coupling with ST3 or in fibronectin recognition and binding, respectively. Proteins were incubated with TEV at 4 °C overnight, and the cleaved His_6*x*_ was separated from the proteins via IMAC. The positively charged, solvent-exposed residues of scGFP in protein **1** were conjugated with the anionic polymer surfactant oxidized IGEPAL CO-890 ([S], Figure S2) in a 1:1.4 ratio following a previously published methodology,^22^ and the excess surfactant was removed by dialysis (20 mM phosphate buffer pH 7.5, 500 mM NaCl). Successful conjugation was confirmed by UV–vis spectroscopy (Figure S3a) with a ratio of 0.9 surfactant molecules per positively charged residue of protein fusion **1**. Dynamic light scattering (DLS) showed an increase of 13% in the hydrodynamic diameter, which corresponds to a polymer surfactant corona, with a thickness of ca. 2 nm, consistent with previously reported dimensions for protein–polymer surfactant complexes^22^ (Figure S3b,c and Table S3). Protein fusions **2** and **3** were also analyzed by UV–vis spectroscopy (Figure S3d) and showed a fivefold difference in absorbances at 280 nm, for equimolar concentrations, which is consistent with a fourfold increase in mass due to the presence of the CshA domain in protein **2** (extinction coefficients at 280 nm calculated by Expasy: EC_2_ = 112,650 M^–1^ cm^–1^, EC_3_ = 37,360 M^–1^ cm^–1^). Significantly, there was no variation in the mCherry chromophore absorbance spectra, which indicated that the fusions were intact and pure. DLS (Figure S3e,f and Table S3) showed that the fusion of CshA (∼84 kDa) to mCherry-ST3 (protein fusion **2**) also increased the hydrodynamic radius by 5.6 nm (cf. protein fusion **3**).

Potential changes in protein folding upon surfactant conjugation to protein fusion **1** and the addition of the highly flexible CshA domain to protein fusion **3** were examined by using circular dichroism (CD) spectroscopy (Figures S4–S6). Surfactant conjugation to protein fusion **1** resulted in a spectrum with little differences to that of the preconjugated protein, i.e., both with spectra typical of proteins with predominately β-sheet secondary structure (Figure S4a,b). Deconvolution of the spectra using the BeStSel algorithm (Figure S5a,b) showed negligible structural changes after surfactant addition. Thermal shift CD plots (Figure S6a,b) showed that both constructs were stable at physiological temperatures up to 54 °C for the unconjugated protein fusion **1**. This is consistent with previously published SC1 and SC2 melting midpoints at 48.5 and 49.9 °C, respectively,^24^ and sfGFP between 76 and 78 °C.^27^ It was notable that the presence of surfactant introduced higher variability in the ellipticity at 217 nm; however, it was considered that both showed the same trend and the surfactant had little effect on the secondary structure of the protein overall. In contrast, the CD spectra of protein fusions **2** and **3** show significant differences (Figure S4c,d), with mCh-ST3 exhibiting an expected high content of β-sheets (48%, Figure S5c) similarly to SC3-scGFP (40%), while CshA-mCh-ST3 was dominated by random-coil features (44%, Figure S5d) due to the intrinsically disordered NR1 domain of CshA.^26^ Other distinguishable features were the β-barrel of mCherry (33%) and α-helical regions of the NR2 and NR3 domains of CshA (10%), which agrees with previously published data.^22^ Both fusion proteins were also stable at physiological temperatures (Figure S6c,d). Interestingly, protein fusion **2** showed no sign of unfolding, as the main features in the spectra correspond to the disordered NR1 domain (Figure S6d), while protein fusion **3** started to lose ellipticity at 217 nm after ca. 85 °C, corresponding to the reported mCherry melting transition (94 ± 7 °C).^28^ Overall, CD spectroscopy confirmed that the protein fusions possessed the secondary structure from their constituent proteins and that they were stable at biologically relevant temperatures (35–40 °C). Fluorescence spectroscopy (Figure S7a) showed that GFP maintained its excitation and emission profiles despite fusion with SC3 and conjugation with the surfactant, with an excitation and emission maxima at 487 and 510 nm, respectively, which is consistent with the Stokes shift value reported for scGFP.^29^ Likewise, mCherry preserved its reported Stokes shift values^30^ of 587 nm excitation and 610 nm emission and spectral profiles upon fusion with ST3 and CshA (Figure S7b), confirming that the fusion to other proteins or the conjugation to the surfactant had no effect on mCherry and GFP tertiary structure and subsequent fluorophore formation.

### Bioorthogonal Coupling of the Fusion Protein Modules

**Figure 2:**
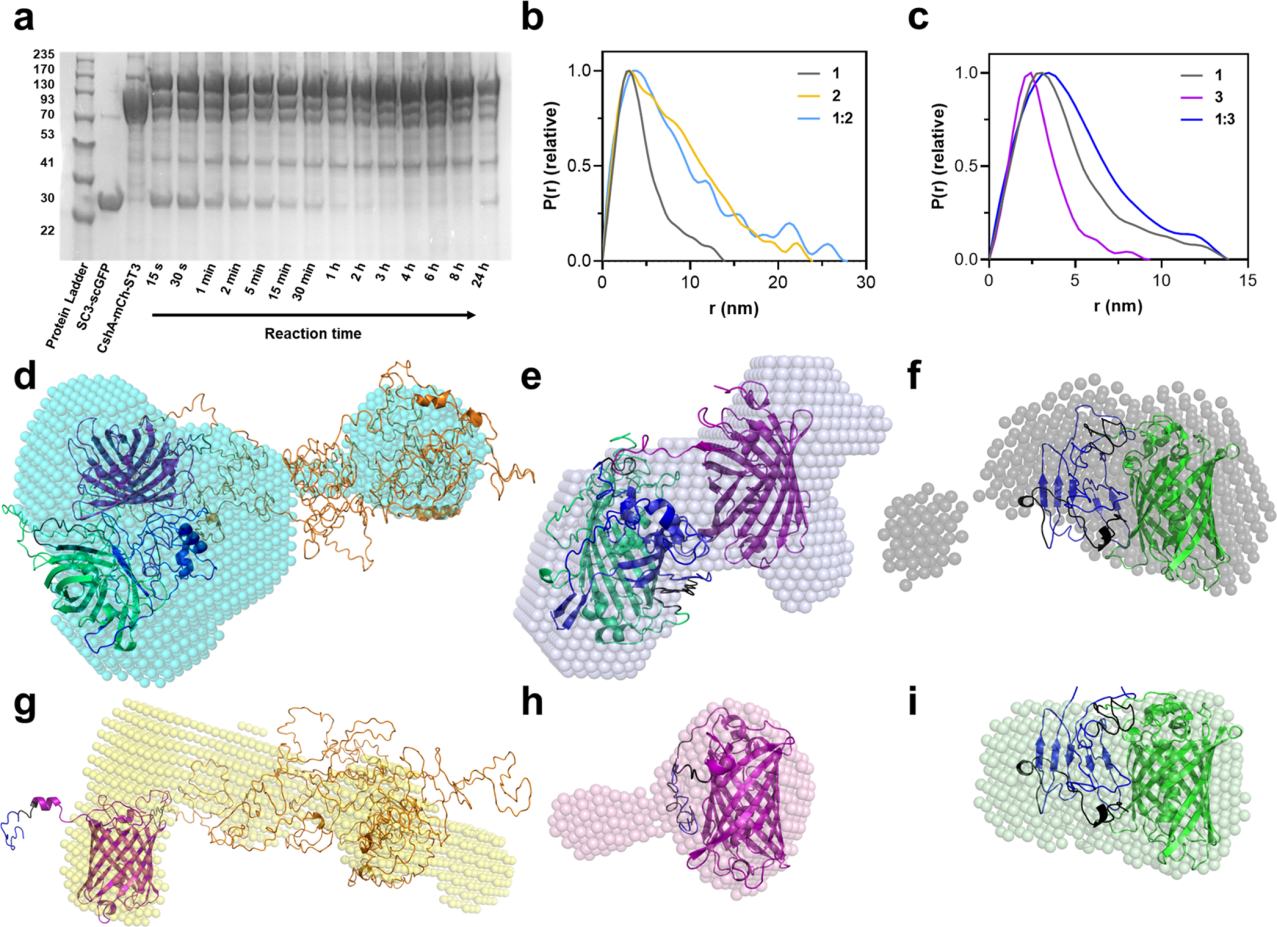
Characterization of reaction between protein constructs containing SpyCatcher and SpyTag. (a) SDS-PAGE monitoring of the reaction between **1** (10 μM [SC3-scGFP][S], ca. 42.5 kDa) and **2** (10 μM CshA-mCh-ST3, ca. 112 kDa) to yield **1:2** (CshA-mCh-ST3-[SC3-scGFP][S], ca. 155 kDa). (b) Pair-distance distribution function *P*(r) calculated using ScÅtter from synchrotron radiation small-angle X-ray scattering (SR-SAXS) data of **1** (shown in gray, χ^2^ = 1.396), **2** (shown in yellow, χ^2^ = 1.255), and the reaction product between both **1:2** (shown in light blue, χ^2^ = 1.292). (c) Pair-distance distribution function *P*(r) calculated using ScÅtter from synchrotron radiation small-angle X-ray scattering (SR-SAXS) data of **1**, **3** (mCh-ST3, shown in purple, χ^2^ = 1.110), and the reaction product between both **1:3** (mCh-ST3-[SC3-scGFP][S], shown in navy blue, χ^2^ = 1.245). (d–h) Overlay of protein crystal structures and ab initio bead models computed from the SR-SXS data of (d) reaction product of CshA-mCh-ST3 and [SC3-scGFP][S] (**1:2**, bead model shown in cyan), (e) reaction product of mCh-ST3 and [SC3-scGFP][S] (**1:3**, bead model shown in navy blue), (f) [SC3-scGFP][S] (**1**, bead model shown in gray), (g) CshA-mCh-ST3 (**2**, bead model shown in orange), (h) mCh-ST3 (**3**, bead model shown in purple), and (i) SC3-scGFP (bead model shown in green). Proteins were modeled with I-TASSER,^32−34^ and the models were selected depending on the known structures of SC3–ST3 (shown in navy blue), sfGFP (shown in green), and mCherry (shown in yellow). I-Tasser model C-scores: (d) −3.45, (e) −2.62, (f–i) −2.62, (g) −4.05, and (h) −0.62.

Reaction of fusion protein **1** with either fusion **2** or **3** was performed under physiological conditions (pH 7.5 at 37 °C) in trisaminomethane buffer (20 mM Tris–HCl, 700 mM NaCl, 10 μM protein) and monitored over 24 h by SDS-PAGE (Figure 2a) in triplicate ( Figure S8). The target coupling product could be observed immediately after mixing, as evidenced in the bands obtained after just 15 s showing 40% formation of **1:2** (CshA-mCh-ST3-SC3-scGFP; ca. 157 kDa) and 60% formation of **1:3** (mCh-ST3-SC3-scGFP; ca. 73 kDa), which were estimated by the pixel intensity of each band using GelAnalyzer software (Figure S8, Materials and Methods section described in the SI). Respective yields for **1:2** and **1:3** were approximately 80% and 60% after 24 h. Overall, the performance was similar to that by Howarth et al.^25^ for the third-generation SpyCatcher-SpyTag system. The difference in yields between the reactions can be attributed to the differences between the masses and structures of the protein fusions; i.e., the presence of the 84 kDa intrinsically disordered CshA domain in **2** would reduce the diffusion coefficient and may allow for better availability of ST3 to react with SC3. However, the yields and reaction times were deemed appropriate for downstream experiments.

To probe their structures at higher resolutions, the protein fusion substituents and the surfactant-conjugated coupling reaction products were studied using synchrotron radiation small-angle X-ray scattering (SR-SAXS) (Table S4 and Figure S9). The membrane anchor domain (protein fusion **1**) showed a 10% increase in the radius of gyration (*R*_g_) upon surfactant conjugation (from 27.4 ± 0.6 to 30.1 ± 0.6 Å), which was accompanied by a 5.5 Å increase (133.5 to 139 Å) in the *D*_max_ (maximum distance between two atoms), which was calculated from the pair-distance distribution *P*(r) (Figures 2b,c and S10). These data indicated that the surfactant electrostatically bound to the anchor domain to form a compact polymer surfactant corona.^31^ The Spy-tagged coupling partners CshA-mCh-ST3 (protein fusion **2**) and mCh-ST3 (protein fusion **3**) had *R*_g_’s of 58 ± 3 nm and 21.48 ± 0.09 Å and *D*_max_ values of 239.5 and 95.5 Å, respectively, which demonstrated that the large intrinsically disordered domain of CshA maintained an extended configuration.^22^

The *R*_g_ and *D*_max_ values obtained after the bioorthogonal coupling of **1** with **3** to yield **1:3** (mCh-ST3:[SC3-scGFP][S]) were 32 ± 1 and 48.4 Å, respectively. Bioorthogonal coupling of **1** with **2** to yield **1:2** (CshA-mCh-ST3:[SC3-scGFP][S]) gave respective *R*_g_ and *D*_max_ values of 51 ± 4 and 279 Å (Figure 2c). The dimensional parameters following the coupling reaction to yield **1:2** were lower than anticipated when considering the dimensions of the constituent proteins (Figure 2b). However, as the *R*_g_ represents the distribution of atoms around the center of mass, the flexibility of the fusion protein can affect this parameter. Indeed, large variations in *R*_g_ within the same protein are not unprecedented and have been attributed to conformational changes resulting from intramolecular arrangements of unstructured protein regions^35^ or changes in the protein surface charge guided by mutagenesis, leading to more compact structures.^36^ Therefore, it is hypothesized that the apparent contraction in the *R*_g_ arose from conformational changes in the highly flexible NR1 “catch” domain in the CshA-containing proteins. These differences in apparent protein dimensions can be observed when overlaying models of the proteins generated using I-TASSER and the ab initio bead model calculated from the diffraction data of protein fusions (Figure 2d–i). The reaction products (d and e) yield more compact structures, especially when compared to the highly elongated protein fusion **2** (Figure 2f).

The differences in flexibility between the constructs were also evident from their dimensionless Kratky plots (Figure S11). Protein fusion **2** (Figure S11d) and the bioorthogonal coupling reaction products **1:2** and **1:3** (Figure S11e,f) displayed wide, asymmetric peaks, indicative of a flexible protein, and possess several shoulders that indicate the presence of multiple domains within the proteins. In contrast, protein fusion **3** (Figure S11c) and both the free and surfactant-conjugated fusion **1** (Figure S11a,b) display profiles typical of folded globular proteins, with an asymmetric tail that indicates some flexibility. This change in flexibility was also reflected in the Porod exponents (*P*_E_), which increased from 2.7 to 3.1 after surfactant conjugation. Protein fusion **3** (*P*_E_ = 4) was more rigid, and this is not surprising given that mCherry is known to fold in a rigid β barrel, and the only feature that confers this fusion protein some flexibility would be ST3, which represents less than 7% of the total protein structure. Conversely, highly flexible proteins exhibit *P*_E_ values closer to 2, which was the case for protein fusion **2** (*P*_E_ = 2.1) and reaction products **1:2** (*P*_E_ = 2.1) and **1:3** (*P*_E_ = 2.4).

### Production and Characterization of Exosome-Inspired Cardiac-Homing Lipid Nanoparticles (CH-LNPs)

LNPs were synthesized by ten cycles of lipid extrusion through a 100 nm membrane following a standard protocol.^37^ The LNP composition was selected to represent the distribution of the most abundant lipids found in human exosomes.^38^ This resulted in LNPs comprising cholesterol (CHOL), 1,2-dioleoyl-*sn*-glycero-3-phospho-choline (DOPC), 1,2-dioleoyl-*sn*-glycero-3-phosphoethanol-amine (DOPE), and 1,2-dioleoyl-*sn*-glycero-3-phospho-l-serine (DOPS) in a 55:21:16:8% ratio with a total lipid concentration of 0.2 mM. DLS and ZetaView nanoparticle tracking (NTA) gave a hydrodynamic diameter and a concentration range of 143.3 ± 40.8 nm and 0.96 × 10^12^ to 1.6 × 10^12^ LNPs/mL, respectively (Table S5). This was in good agreement with the theoretical size and composition (eqs S1 and S2 and Figures S12–S13) based on the formulation, and a particle number density of 1.5 × 10^12^ LNPs/mL was used for all subsequent calculations. DLS also showed that the LNP formulation was stable after 24 h at 37 °C and 7 days at 4 °C and was compatible with centrifugation, dialysis, culturing cell media, UV sterilization, and 0.22 μM filtration (Figure S14 and Table S6).

**Figure 3:**
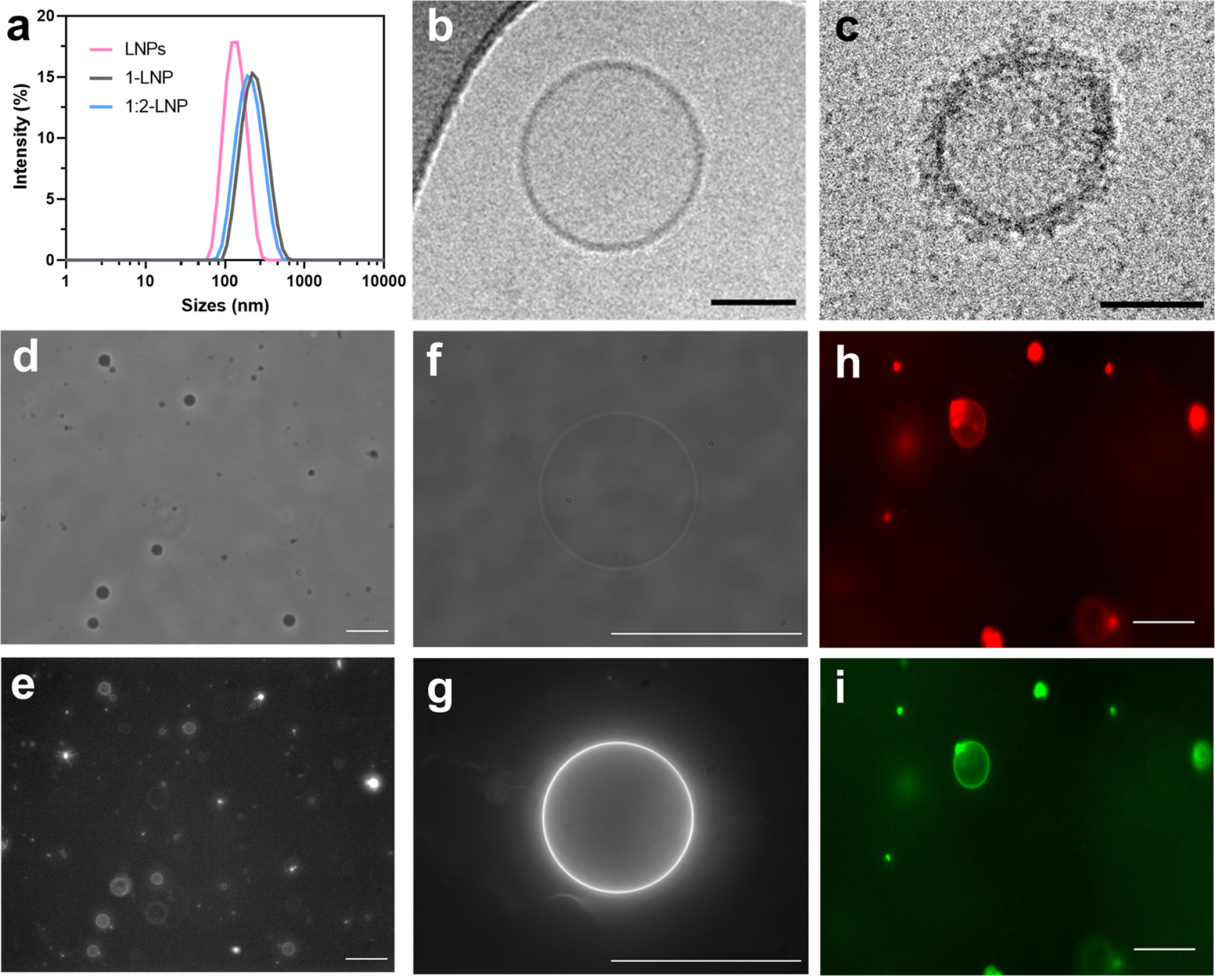
Characterization of LNPs and their modification with AMBPs. (a) Dynamic light scattering (DLS) of native LNPs (approximately 1.5 × 10^12^ LNPs/mL) or AMBP-modified LNPs (10 μM AMBP concentration, 100 μL protein per 1 mL of LNPs). (b) Cryo-TEM images of unmodified LNPs (approximately 1.5 × 10^13^ LNPs/mL) and (c) **1:2**-LNPs (approximately 1.5 × 10^13^ LNPs/mL) showing successful membrane modification. Scale bars: 50 nm. (d) Phase-contrast microscopy image of giant unilamellar vesicles (GUVs) after an overnight reaction with 2 μL of **4** (10 μM). (e) Fluorescence microscopy image of GFP from (d). (f) Phase-contrast image of a single GUV modified with **4**. (g) GFP fluorescence microscopy image of (f). (h) Fluorescence microscopy images (GUVs) labeled with Texas red (shown in red) and after addition of 2 μL of **5** (10 μM). (i) GFP channel of (h), shown in green. GUVs were composed of CHOL, DOPC (5% TR-DHPE only in (h) and (i)) DOPS, and DOPE in a 55:21:16:8% ratio, and lipid films were hydrated in 0.5 M sucrose and imaged in 0.5 M glucose (both in 20 mM phosphate buffer at pH 7.5). Scale bars: 50 μm.

Considering that the barrel-shaped membrane anchor domain of protein fusion **1** could theoretically bind to the LNP bilayer via a range of orientations (Figure S13), the theoretical protein monolayer concentration range was 2 μM (end-on binding) to 16 μM (side-on binding) (eqs S3–S6). Accordingly, 100 μL of the anchor domain [scGFP][S] in concentrations varying from 1 to 50 μM was reacted with 1 mL of LNPs (1.5 × 10^12^ LNPs/mL) overnight at 4 °C to determine the optimal protein concentration (Figure S15). Modified LNPs were then washed with phosphate buffer twice and centrifuged in a Vivaspin filter to remove unbound protein. DLS showed that the LNPs are stable in all of the protein concentrations tested, and both UV–vis and fluorescence spectroscopy showed that protein saturation was achieved at ca. 10 μM [scGFP][S], which was selected as the protein incubation concentration for all subsequent experiments. LNPs were reacted with 10 μM of either protein fusion **1** or protein fusion **1:2** following the same protocol (Figure 3a and Table S7), exhibiting an increase in the LNP hydrodynamic diameter for both conditions from 143.3 ± 40.8 to 253.4 ± 82.0 and 221.4 ± 71.7 nm, respectively.

Cryogenic electron microscopy (cryo-EM) images of unmodified LNPs (Figure 3b) showed an LNP membrane thickness of approximately 5.4 ± 0.9 nm, with particle diameters ranging from 32.7 to 121.8 nm (mean of 82.3 ± 21.9 nm) (Figure S16). LNPs modified with the **1:2** chimera (Figure 3) exhibited higher contrast membranes with a rough outer layer thickness ranging from 3.9 to 41.6 nm (mean of 10.0 ± 5.6 nm, Figure S16 and Table S8). Interestingly, **1:2**-LNPs were found to range in size from 38.9 to 213.1 nm with a mean of 92.6 ± 34.6 nm, resulting in no statistical difference in size compared to unmodified LNPs. This could perhaps be explained by LNP fusion and aggregation, which was observed in both unmodified and modified LNPs (Figures S17 and S18). Similar results were obtained in the control samples of **1**-LNPs and **1:3**-LNPs (Figures S16–S18 and Table S8) where the size of the LNPs was unchanged compared to native LNPs, but the thickness of the membrane increased to 7.1 ± 2.5 and 10.4 ± 2.8 nm, respectively.

Giant unilamellar vesicles (GUVs) are commonly used as biological membrane models in phase-contrast microscopy studies, as they range in size from 1 to 200 μm. Accordingly, GUVs were synthesized using the same LNP lipid formulation to directly image the GFP membrane anchor proteins (Figure 3d–i). GUVs were synthesized using electroformation following an adapted protocol outlined by Li et al.,^39^ with an extended electroformation period, obtaining diameters between approximately 4 and 30 μm (Figure S19). The membrane anchor domain **5** was added to the synthesized GUVs and imaged immediately after mixing (Figure S20). Interestingly, membrane binding by **5** to GUVs was highly selective, where only 10% of the GUVs were successfully modified with AMBP. Conversely, incubation overnight resulted in 100% of the GUVs exhibiting GFP fluorescence. It is hypothesized that a cooperative effect occurs, where the initial interaction between one AMBP and the lipid bilayer causes the lipid hydrocarbon chains to splay, reducing the lateral chain stress and simultaneously increasing the lateral pressure across the headgroups, driving further AMBP insertion. This behavior has been reported when nonbilayer lipids are incorporated in LNPs.^40,41^ The initial discrimination of the AMBP over certain GUVs could also be the result of electroformation-derived lipid heterogeneity,^42^ yielding enriched areas that are more susceptible to AMBP insertion, but considering this effect is overruled overnight, the optimal incubation of AMBPs with LNPs was determined to be overnight at 4 °C. Testing of fusion protein AMBPs such as **4** also showed full GUV coverage after overnight incubation (Figure 3d–f), suggesting that the size and nature of the AMBP has no effect on the LNP modification. Crucially, we showed no evidence of AMBP internalization, even upon fusion of GUVs (Figure S21), which agrees with previous studies performed on hMSCs where [CshA-scGFP][S] remained in the membrane for at least 12 h.^22^ Colocalization assays were also performed by labeling the GUV membanes with Texas red (TR)-modified 1,2-dihexadecanoyl-*sn*-glycero-3-phosphoethanolamine (TR-DHPE) (Figure 3h,i), which confirmed that the anchor was bound to the membrane. While TR-DOPC was observed throughout all the GUV membranes, the distribution was irregular, with some areas displaying more fluorescence than others, which reinforces the previous hypothesis whereby the AMBP initially inserts preferentially in certain membrane regions of specific GUVs.

### C2C12 Myoblast Interaction with AMBP-Modified LNPs

Upon myocardial infarction, endogenous cardiomyocytes undergo necrosis due to the lack of oxygen and nutrients in the injury site and are quickly replaced by recruited myofibroblasts, which commence a process of fibrosis to heal the injury by secreting protein fibers like collagen and fibronectin.^43^ Accordingly, mouse C2C12 myoblasts were selected for this study, as they are a well-characterized adherent cell line known to express fibronectin and produce fibrotic structures as part of their extracellular matrix (ECM).^44^ Moreover, this makes them suitable candidates to assess the interaction of LNPs equipped with a fibronectin-binding motif (CshA) via the AMBPs. It was hypothesized that if LNP binding to the C2C12 cells occurred, the targeted LNPs would be internalized by the cells.

**Figure 4:**
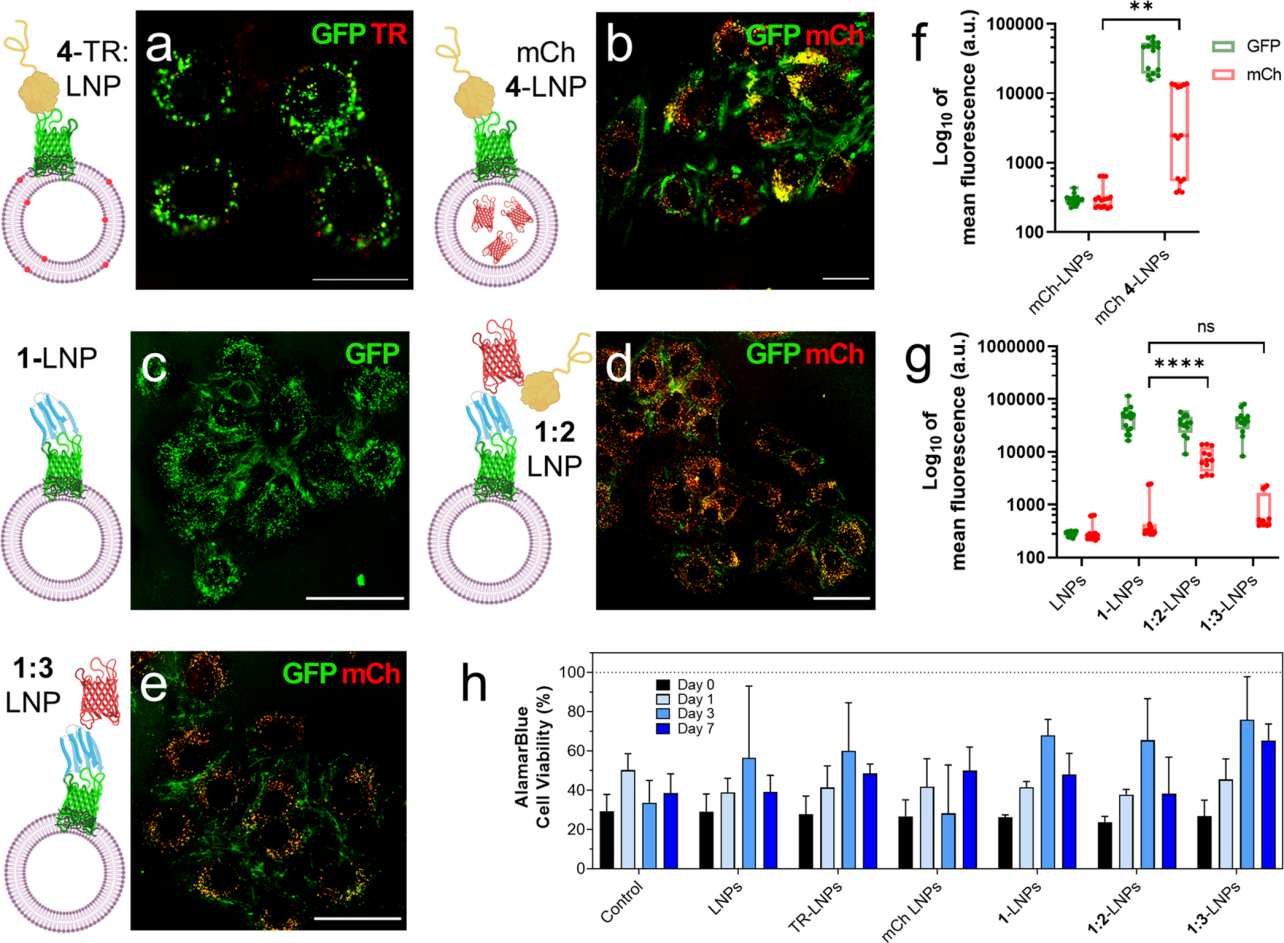
Characterization of AMBP-modified LNPs interaction with C2C12 myoblasts. Confocal images of C2C12 cells (250 000 cells/dish) after exposure to (a) Texas red (TR)-labeled **4**-LNPs, (b) mCherry-loaded **4**-LNPs, (c) **1**-LNPs, (d) **1:2**-LNPs, and (e) **1:3**-LNPs (approximately 1.5 × 10^11^ LNPs). GFP is shown in green, and mCherry or Texas red is shown in red. Scale bars: a–b, 25 μm; c–e, 50 μm. (f) Flow cytometry quantification of GFP (shown in green) and mCherry (shown in red) in C2C12 cells after 2 h of incubation with mCherry-loaded LNPs (*n* = 15) or (g) LNPs modified with protein fusions **1**, **1:2**, and **1:3** (*n* = 12). Box and whiskers plots display min to max error bars and data points. Each data point is the mean of fluorescence of 10 000 events. Multiple unpair *t* tests are displayed as n.s. for nonsignificance, **p < 0.01 and ****p < 0.0001. (h) Cell viability of C2C12 myoblasts (50 000 cells/well, 96-well plate) over 7 days after exposing them to different conditions (20 μL of 1 μM proteins or approximately 1.5 x 10^10^ vesicles) for 2 h. Cell viability (*n* = 3) was calculated via extrapolation of the fluorescence data obtained from the Alamar Blue (AB) metabolic activity assay from a previously calculated standard curve. The mean and standard deviation are reported. LNP diagrams were created with Biorender.com.

LNP membranes were labeled with TR-DHPE, and live cell confocal microscopy experiments were performed. C2C12 myoblasts imaged after a 2 h incubation period with either TR:LNPs or **4**-TR:LNPs (Figures 4a and S22a,b) revealed that only the AMBP-coated LNPs interacted with the cells (1.5 × 10^11^ LNPs per 250,000 cells). The apparent GFP (from the anchor domain of the AMBP) and TR fluorescence colocalization in individual dots in the cytosol confirmed that the LNPs were endocytosed. LNPs were also loaded with mCherry, and only the LNPs coated with protein fusion **4** were detected inside the cells (Figures 4b and S22c,d), confirming that the AMBP presence is essential for the LNPs to interact with the cell and being endocytosed. Image analysis was not sensitive enough to determine whether there is a successful endosomal escape and consequent delivery of mCherry to the cytosol, but visual analysis of the mCherry channel suggests a more evenly distributed fluorescent signal throughout the cytosol compared to GFP, which was more concentrated in “dots” (cf. Figure S22b,d). It is possible that the AMBP remained associated or intercalated with endosomes after the release of the mCherry cargo to the cytosol. Although beyond the scope of this research, other cargos with cytosolic functions could be tested to help ascertain this mechanism. The difference in mCherry fluorescence between the unmodified and coated LNPs was quantified using flow cytometry (Figure 4f), confirming that the targeted LNPs were capable of delivering the protein cargo to the cytosol.

LNPs coated with the protein fusions containing AMBP **1** displayed similar behavior to the **4**-LNPs and were localized as small dots in the cytosol of the C2C12 myoblasts (Figures 4c-e and S22e-f), with no apparent difference in cellular uptake between the **1**-, **1:2**-, or **1:3**-coated LNPs when comparing the GFP fluorescence inside the cells by flow cytometry (Figure 4g). This is not entirely surprising, as even though **1**- and **1:3**-LNPs do not contain the CshA sequence, they still possess SC3, which is the result of engineering the bacterial adhesin FbaB that binds strongly to human fibronectin in endothelial cells.^45^ Interestingly, our results infer that the mutations present in SC3 did not eliminate the fibronectin-binding capability under these experimental conditions; however, the fibronectin affinity of the complex formed between SC3 and ST3 seems to be impaired in **1:3**-LNPs based on the significant decrease in mCherry fluorescence. Previous experiments showed that the reaction between **1** and **3** to give **1:3** had a yield of 60% after 24 h, while the yield from **1:2** formation was 80% (Figure S8). Therefore, if SC3–ST3 displayed the same fibronectin affinity as the SC3 in **1**-LNPs, a similar or higher ratio between GFP and mCherry fluorescence should have been detected in cells treated with **1:3**-LNPs, as was the case for **1:2**-LNPs. Accordingly, it is hypothesized that SC3 affinity could be diminished by its location in between GFP and mCherry in the **1:3** fusion, leading to endocytosis of **1**-LNPs formed with unreacted **1** and partial uptake of the **1:3**-LNP population, resulting in a small increase in mCherry fluorescence and a much higher GFP to mCherry fluorescence ratio. This effect can be clearly seen in the confocal images, where there is a complete GFP and mCherry colocalization in cells treated with **1:2**-LNPs, while cells subjected to **1:3**-LNPs portrayed green fluorescence across the cytosol with some mCherry colocalization near the nuclear envelope (Figure S22). Surprisingly, the same behavior was observed in cells treated directly with the fusion proteins via confocal microscopy (Figures S23), showing no internalization of proteins **2** and **3**, as they lack a membrane anchoring domain, but all of the AMBP samples displayed the chromophore fluorescence as dots in the cytosol, instead of the expected localization on the cell membrane as previously reported in patient-derived mesenchymal stem cells (MSCs) with AMBP **4**(22) and other AMBPs.^18,21^ However, the protocol employed in this work retains the same incubation times optimized for LNPs and low concentrations compatible with clinical translation, while AMBP cell coating is usually performed on cells in suspension at high concentrations, possibly removing in this protocol the effect of self-assembly effects between protein units, favoring their anchoring on the cell membrane. Additionally, C2C12 myoblasts are a cell line that has been shown to have considerably higher transfection efficiencies using liposomes than primary cells, suggesting a much higher rate of endocytosis, which could also contribute to the observed outcome.^46^ Interestingly, cells treated with either protein fusion **5** or **5**-LNPs displayed a 100-fold decrease in GFP fluorescence compared to protein fusion **1** samples in flow cytometry (Figure S24), suggesting that endocytosis could be driven by the fibronectin-binding interactions instead of the AMBP anchoring to the cell membrane. Finally, cell viability assays after 2 h of cell incubation showed that none of the different conditions had a significant detrimental effect on the cell metabolic activity over 7 days (Figures 4h and S25).

### CH-LNPs Interact with C2C12s More Favorably at Biologically Relevant Shear Stress

**Figure 5:**
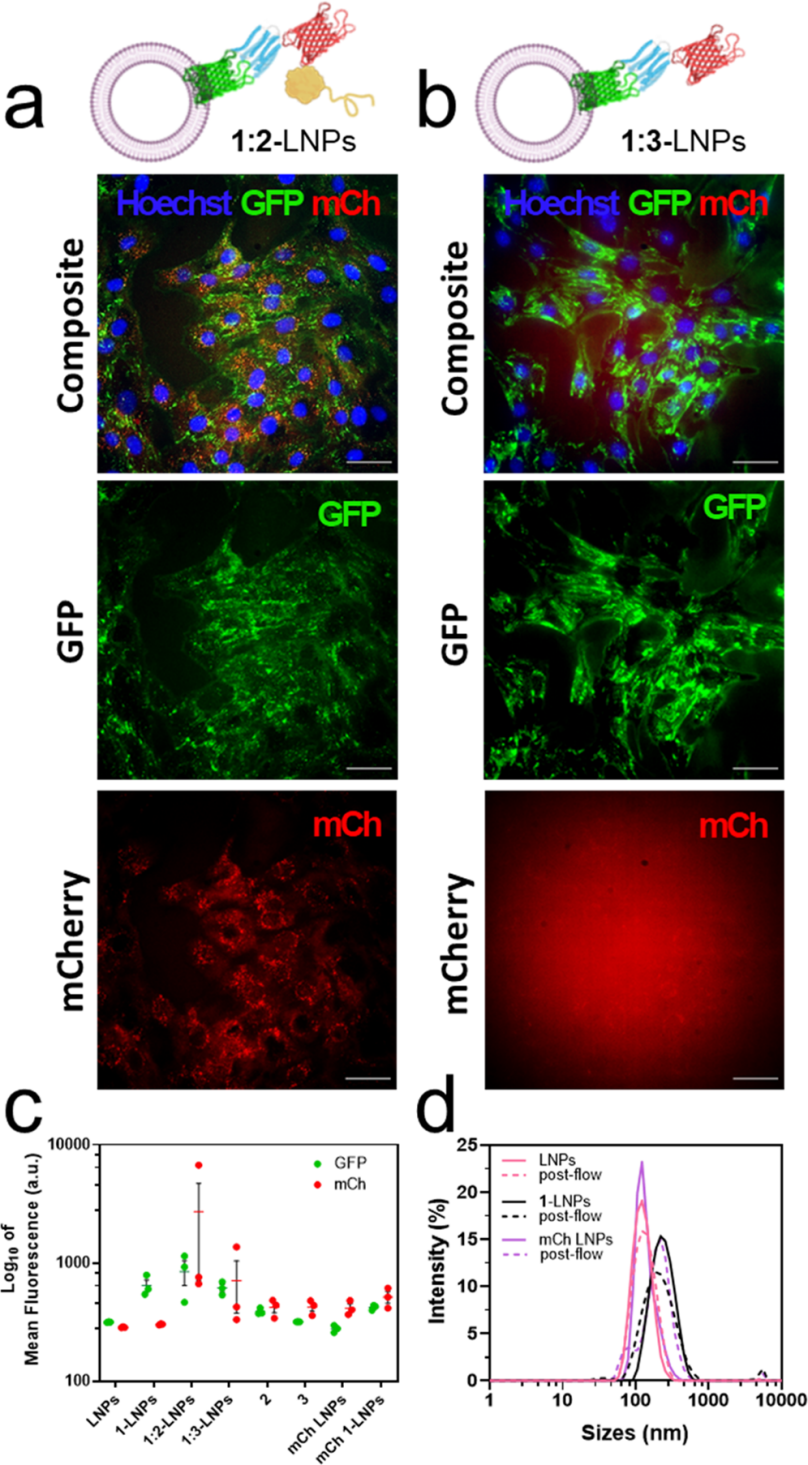
Characterization of AMBP-modified LNPs interaction with C2C12 myoblasts in flow. (a) End-point wide-field microscopy image of C2C12s cells in a flow channel after exposure with **1:2**-LNPs or (b) **1:3**-LNPs (250,000 cells per flow channel, approximately 1.5 × 10^11^ vesicles in 25 mL of DMEM) at a shear stress of 2 dyn for 15 min at 37 °C. GFP is shown in green, mCherry or Texas red is shown in red, and Hoechst 33342 is shown in blue. Scale bars: 50 μm. (c) Flow cytometry quantification of GFP (shown in green) and mCherry (shown in red) in C2C12 cells after the flow experiment (*n* = 3). Plot displays the mean ± SEM, and data points are shown. Each data point is the mean of fluorescence of 10,000 single-cell events. (d) DLS intensity data of **1**-LNPs before and after exposure to 2 dyn stress shear showing that they maintain their size. LNP diagrams were created with Biorender.com.

The previous static experiments revealed that all of the LNPs coated with proteins containing either SC3 or CshA in the N-terminal of the fusion proteins bind to C2C12 myoblasts and are internalized; however, an important aspect to consider for clinical relevance is the effect of flow-based shear stress.^47^ Consequently, C2C12 myoblasts were allowed to adhere to flow channels (Ibidi μ-slides, 250,000 cells/channel) and subjected to shear stress (2 dyn) and monitored in real time using wide-field microscopy (Figure S26). As under static conditions, colocalization of GFP and mCherry was observed only for **1:2**-LNPs (Figure 5a and Videos S1 and S2), and only GFP fluorescence was detected in cells treated with **1:3**-LNPs (Figure 5b and Videos S3 and S4). This suggested that both N-terminal SC3 and CshA are capable of binding to the cells for the sufficient time required for internalization, and no significant differences were observed between them after flow cytometry analysis (Figure 5c). Interestingly, the GFP fluorescence in C2C12 myoblasts decreased by approximately 50-fold compared with that in the equivalent static experiments (Figure 4g). This was likely due to a decrease in the number of binding events under flow conditions resulting from the higher dilution factor required for the flow assay. This large decrease in the fluorescent signal is believed to impact the results obtained for mCherry-loaded LNPs in this experiment, as no significant differences between uncoated and protein fusion **1**-coated LNPs were obtained by flow cytometry, as opposed to the static experiments. Therefore, we could not determine whether the current system is able to deliver cargo inside cells under flow as the signal strength was likely below the detection limit. Comparison between the LNPs DLS before and after being subjected to an acellular flow channel showed no significant differences between the size of uncoated LNPs and LNPs modified with protein fusion **1** (Figure 5d). This was not unexpected, as LNP scattering could be observed as green circumferences when the flow stopped between **1:2**-LNP sample application and the first DMEM wash (Figure S26). mCherry-loaded LNPs increased in size after flow to a similar size to **1**-LNPs, and therefore, it was an acceptable difference between both samples, and they were considered to be intact.

### AMBP-Modified LNPs Exhibit Selective Homing to Zebrafish Cardiac Tissue

**Figure 6:**
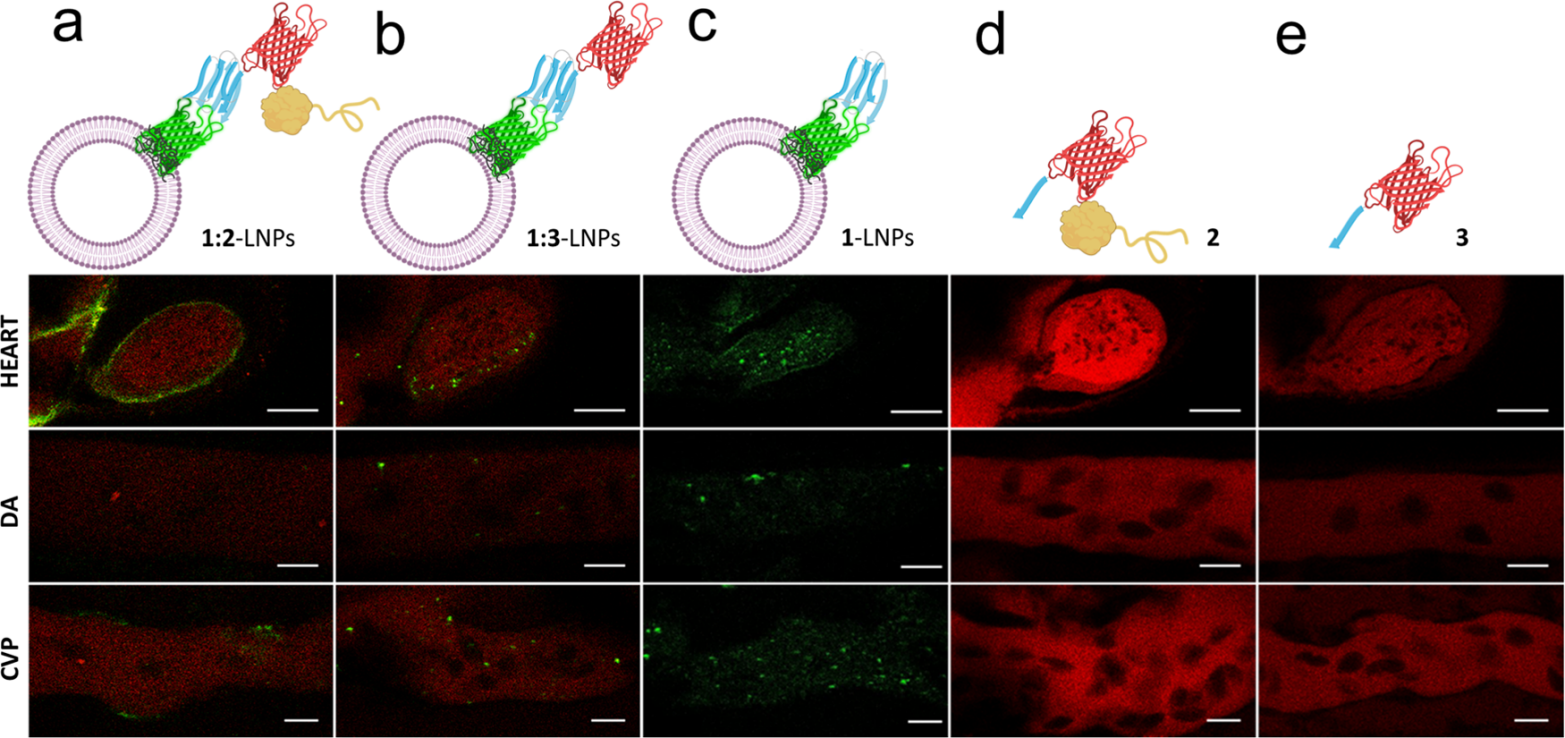
Live imaging of proteins and modified LNPs in the peripheral circulation of 2 days postfertilization larval zebrafish. Modified LNPs (2 nL, approximately 3 × 10^6^ vesicles) or control proteins (2 nL, 30 μM) were injected into the duct of Cuvier and monitored in the heart, in the arterial environment of the dorsal aorta (DA), and in venous vessels in the caudal vein plexus (CVP). Live imaging was performed on a Leica TCS SP8 AOBS confocal laser scanning microscope. GFP is shown in green, and mCherry is shown in red. The images shown are representative of each condition. Scale bars: 50 μm for the heart, 10 μm for DA and CVP images.

A two-day postfertilization larval zebrafish model was used to evaluate the cardiac-homing potential of the AMBP-coated LNPs via live imaging (Figure S27a). The fibronectin sequence is highly conserved among higher animals^48^ and so was thought to be an adequate target for CshA and SpyCatcher in zebrafish and mice.^22^ Larval zebrafish are particularly relevant for this study due to their amenability to manipulation and live imaging, and fibronectin (Fn) is especially abundant during developmental cardiac remodeling. Preliminary experiments showed that the concentration of LNPs was insufficient to observe fluorescence after 2 nL injections; therefore, LNPs were produced at tenfold of the initial number density, which did not have any significant effect on their size (DLS, Figure S27b and Table S9). Modified LNPs (2 nL, approximately 3 × 10^6^ LNPs), or control proteins (2 nL, 30 μM), were microinjected into the duct of Cuvier and monitored in the heart, in the arterial environment of the dorsal aorta (DA), and in venous vessels in the caudal vein plexus (CVP) (Figure 6). Significantly, **1:2**-LNPs showed evidence of GFP and mCherry colocalization in the heart throughout the imaging, indicating retention (Figure 6a and Videos S5, S6, and S7). While no LNPs were detected in the DA, surprisingly, many were discovered in the CVP, which was attributed to the presence of Fn produced by hematopoietic stem cells and stromal cells.^49^ Crucially, the live images here appeared like those reported for PEGylated liposomes used as nanoparticulate delivery systems^50^ and fluorescently labeled endogenous EVs in circulation in zebrafish,^51^ rather than the behavior of nanoparticles undergoing macrophage clearance.^52^ In contrast, **1:3**-LNP retention in the heart was significantly diminished, with little colocalization and mainly GFP fluorescence present in the heart (Figure 6b and Videos S8, S9, and S10), similar to **1**-LNPs (Figure 6c and Videos S11, S12, and S13), which agrees with the previous findings in C2C12 myoblasts, suggesting that most LNPs retained are unreacted **1**-LNPs. An increase in granular GFP fluorescence in circulation in the DA and CHT was observed in both conditions compared to **1:2**-LNPs, possibly a result of the nonspecific Fn affinity of SC3 adhering to plasma Fn or other ECM components in circulation. Indeed, CshA has a proven increased affinity for immobilized Fn than plasma fibronectin,^26^ explaining why **1:2**-LNPs were retained primarily in the heart. Background mCherry fluorescence was observed for both **2** (Figure 6d and Videos S14, S15, and S16) and **3** (Figure 6e and Videos S17, S18, and S19) in the heart, CVP, and DA; however, the fluorescence for the CshA-containing protein fusion **2** was much higher in the heart, suggesting that the protein alone also has a high affinity for the cardiac immobilized Fn.

## Conclusions

In conclusion, we have described the rational design of a modular membrane modification platform for LNPs that utilizes the bioorthogonal SC3/ST3 system and that does not damage the integrity of the LNP membrane. The modified LNPs were shown to specifically bind to and get internalized by C2C12 myoblasts with no loss of cell viability in both static experiments and flow cytometry experiments under biologically relevant shear stresses. Furthermore, an in vivo zebrafish model revealed accumulation of the surface-modified LNPs with cardiac homing containing proteins predominantly on the heart with minimal off-target localization or evidence of clearance by the reticuloendothelial system (RES), as is often the case with extracellular vesicle-based therapies. Our findings highlight the potential of our modular AMBP technology in combination with LNPs as an off-the-shelf vehicle for therapeutic cargos that can be easily functionalized with different modules depending on the desired outcome, as evidenced in this work using this technology as a cardiac-homing vector with high specificity in vivo.

## Supporting Information Available

Live microscopy of C2C12 myoblasts (250,000 cells) in the flow channel during the sample application of **1:2**-LNPs (approximately 1.5 × 10^10^ vesicles in 25 mL of DMEM) for 15 min at 2 dynes shear stress (MP4)
Live microscopy of C2C12 myoblasts (250,000 cells) in flow channel after sample application of **1:2**-LNPs for 15 min flowing DMEM (25 mL) at 2 dynes shear stress (MP4)
Live microscopy of C2C12 myoblasts (250,000 cells) in flow channel during the sample application of **1:3**-LNPs (approximately 1.5 × 10^10^ vesicles in 25 mL of DMEM) for 15 min at 2 dynes shear stress (MP4)
Live microscopy of C2C12 myoblasts (250,000 cells) in flow channel after sample application of **1:3**-LNPs for 15 min flowing DMEM (25 mL) at 2 dynes shear stress (MP4)
Live microscopy of the heart of a 2 day postfertilization larval zebrafish after injection of **1:2**-LNPs (2 nL, approximately 3 × 10^6^ vesicles) (MP4)
Live microscopy of the dorsal aorta of a 2 day postfertilization larval zebrafish after injection of **1:2**-LNPs (2 nL, approximately 3 × 10^6^ vesicles) (MP4)
Live microscopy of the caudal vein plexus of a 2 day postfertilization larval zebrafish after injection of **1:2**-LNPs (2 nL, approximately 3 × 10^6^ vesicles) (MP4)
Live microscopy of the heart of a 2 day postfertilization larval zebrafish after injection of **1:3**-LNPs (2 nL, approximately 3 × 10^6^ vesicles) (MP4)
Live microscopy of the dorsal aorta of a 2 day postfertilization larval zebrafish after injection of **1:3**-LNPs (2 nL, approximately 3 × 10^6^ vesicles) (MP4)
Live microscopy of the caudal vein plexus of a 2 day postfertilization larval zebrafish after injection of **1:3**-LNPs (2 nL, approximately 3 × 10^6^ vesicles) (MP4)
Live microscopy of the heart of a 2 day postfertilization larval zebrafish after injection of **1**-LNPs (2 nL, approximately 3 × 10^6^ vesicles) (MP4)
Live microscopy of the dorsal aorta of a 2 day postfertilization larval zebrafish after injection of **1**-LNPs (2 nL, approximately 3 × 10^6^ vesicles) (MP4)
Live microscopy of the caudal vein plexus of a 2 day postfertilization larval zebrafish after injection of **1**-LNPs (2 nL, approximately 3 × 10^6^ vesicles) (MP4)
Live microscopy of the heart of a 2 day post-fertilization larval zebrafish after injection of protein fusion **2** (2 nL, 30 μM) (MP4)
Live microscopy of the dorsal aorta of a 2 day postfertilization larval zebrafish after injection of protein fusion **2** (2 nL, 30 μM) (MP4)
Live microscopy of the caudal vein plexus of a 2 day postfertilization larval zebrafish after injection of protein fusion **2** (2 nL, 30 μM) (MP4)
Live microscopy of the heart of a 2 day postfertilization larval zebrafish after injection of protein fusion **3** (2 nL, 30 μM) (MP4)
Live microscopy of the dorsal aorta of a 2 day postfertilization larval zebrafish after injection of protein fusion **3** (2 nL, 30 μM) (MP4)
Live microscopy of the caudal vein plexus of a 2 day postfertilization larval zebrafish after injection of protein fusion **3** (2 nL, 30 μM) (MP4)
Materials and methods, gene sequences of proteins of interest, hydrodynamic diameters of LNPs exposed to different conditions calculated from DLS data, list of equations; protein constructs, fluorescence spectroscopy of synthesized proteins, and schematic of a large unilamellar vesicle with a diameter of 100 nm (PDF)

## Abbreviations

AMBP: artificial membrane-binding protein
CD: circular dichroism
CHOL: cholesterol
cryo-EM: cryogenic electron microscopy
CTs: cell therapies
CVD: cardiovascular disease
CVP: caudal vein plexus
DA: dorsal aorta
DLS: dynamic light scattering
*D*_max_: maximum distance
EC: extinction coefficient
DMEM: Dulbecco’s modified Eagle’s medium
ECM: extracellular matrix
EVs: extracellular vesicles
Fn: fibronectin
GFP: green fluorescent protein
GUVs: giant unilamellar vesicles
hMSCs: human mesenchymal stem cells
IMAC: immobilized metal affinity chromatography
LNPs: lipid nanoparticles
mCh: mCherry
P_E_: poroid exponent
RES: reticuloendothelial system
Rg: radius of gyration
RNA: ribonucleic acid
SC3: SpyCatcher03
scGFP: supercharged GFP
SDS-PAGE: sodium dodecyl sulfate-polyacrylamide gel electrophoresis
SEC: size-exclusion chromatography
sfGFP: superfolder GFP
SR-SAXS: synchrotron radiation small-angle X-ray scattering
ST3: SpyTag03
TEV: tobacco-etch virus
TR: Texas red

## References

[ref1] Hou X.; Zaks T.; Langer R.; Dong Y. Lipid Nanoparticles for MRNA Delivery. Nat. Rev. Mater. 2021, 6 (12), 1078–1094. 10.1038/s41578-021-00358-0. 34394960 PMC8353930

[ref2] Sid-Otmane C.; Perrault L. P.; Ly H. Q. Mesenchymal Stem Cell Mediates Cardiac Repair through Autocrine, Paracrine and Endocrine Axes. J. Transl. Med. 2020, 18 (1), 336 10.1186/s12967-020-02504-8. 32873307 PMC7466793

[ref3] Zhang H. C.; Liu X. B.; Huang S.; Bi X. Y.; Wang H. X.; Xie L. X.; Wang Y. Q.; Cao X. F.; Lv J.; Xiao F. J.; Yang Y.; Guo Z. K. Microvesicles Derived from Human Umbilical Cord Mesenchymal Stem Cells Stimulated by Hypoxia Promote Angiogenesis Both in Vitro and in Vivo. Stem Cells Dev. 2012, 21 (18), 3289–3297. 10.1089/scd.2012.0095. 22839741 PMC3516422

[ref4] Gallet R.; Dawkins J.; Valle J.; Simsolo E.; De Couto G.; Middleton R.; Tseliou E.; Luthringer D.; Kreke M.; Smith R. R.; Marbán L.; Ghaleh B.; Marbán E. Exosomes Secreted by Cardiosphere-Derived Cells Reduce Scarring, Attenuate Adverse Remodelling, and Improve Function in Acute and Chronic Porcine Myocardial Infarction. Eur. Heart J. 2017, 38 (3), 201–211. 10.1093/eurheartj/ehw240. 28158410 PMC5837390

[ref5] Ju C.; Li Y.; Shen Y.; Liu Y.; Cai J.; Liu N.; Ma G.; Tang Y. Transplantation of Cardiac Mesenchymal Stem Cell-Derived Exosomes for Angiogenesis. J. Cardiovasc. Transl. Res. 2018, 11 (5), 429–437. 10.1007/s12265-018-9824-y. 30276617 PMC6413496

[ref6] Armstrong J. P. K.; Holme M. N.; Stevens M. M. Re-Engineering Extracellular Vesicles as Smart Nanoscale Therapeutics. ACS Nano 2017, 11 (1), 69–83. 10.1021/acsnano.6b07607. 28068069 PMC5604727

[ref7] Aday S.; Hazan-Halevy I.; Chamorro-Jorganes A.; Anwar M.; Goldsmith M.; Beazley-Long N.; Sahoo S.; Dogra N.; Sweaad W.; Catapano F.; Ozaki-Tan S.; Angelini G. D.; Madeddu P.; Benest A. V.; Peer D.; Emanueli C. Bioinspired Artificial Exosomes Based on Lipid Nanoparticles Carrying Let-7b-5p Promote Angiogenesis in Vitro and in Vivo. Mol. Ther. 2021, 29 (7), 2239–2252. 10.1016/j.ymthe.2021.03.015. 33744469 PMC8261169

[ref8] Wang X.; Hu S.; Li J.; Zhu D.; Wang Z.; Cores J.; Cheng K.; Liu G.; Huang K. Extruded Mesenchymal Stem Cell Nanovesicles Are Equally Potent to Natural Extracellular Vesicles in Cardiac Repair. ACS Appl. Mater. Interfaces 2021, 13 (47), 55767–55779. 10.1021/acsami.1c08044. 34793116

[ref9] Kim M.; Jeong M.; Hur S.; Cho Y.; Park J.; Jung H.; Seo Y.; Woo H. A.; Nam K. T.; Lee K.; Lee H. Engineered Ionizable Lipid Nanoparticles for Targeted Delivery of RNA Therapeutics into Different Types of Cells in the Liver. Sci. Adv. 2021, 7 (9), eabf4398 10.1126/sciadv.abf4398. 33637537 PMC7909888

[ref10] Cheng Q.; Wei T.; Farbiak L.; Johnson L. T.; Dilliard S. A.; Siegwart D. J. Selective Organ Targeting (SORT) Nanoparticles for Tissue-Specific MRNA Delivery and CRISPR–Cas Gene Editing. Nat. Nanotechnol. 2020, 15 (4), 313–320. 10.1038/s41565-020-0669-6. 32251383 PMC7735425

[ref11] Pattipeiluhu R.; Arias-Alpizar G.; Basha G.; Chan K. Y. T.; Bussmann J.; Sharp T. H.; Moradi M. A.; Sommerdijk N.; Harris E. N.; Cullis P. R.; Kros A.; Witzigmann D.; Campbell F. Anionic Lipid Nanoparticles Preferentially Deliver MRNA to the Hepatic Reticuloendothelial System. Adv. Mater. 2022, 34 (16), 2201095 10.1002/adma.202201095. PMC9461706 35218106

[ref12] Evers M. J. W.; Du W.; Yang Q.; Kooijmans S. A. A.; Vink A.; van Steenbergen M.; Vader P.; de Jager S. C. A.; Fuchs S. A.; Mastrobattista E.; Sluijter J. P. G.; Lei Z.; Schiffelers R. Delivery of Modified MRNA to Damaged Myocardium by Systemic Administration of Lipid Nanoparticles. J. Controlled Release 2022, 343, 207–216. 10.1016/j.jconrel.2022.01.027. 35077739

[ref13] Rurik J. G.; Tombácz I.; Yadegari A.; Fernández P. O. M.; Shewale S. V.; Li L.; Kimura T.; Soliman O. Y.; Papp T. E.; Tam Y. K.; Mui B. L.; Albelda S. M.; Puré E.; June C. H.; Aghajanian H.; Weissman D.; Parhiz H.; Epstein J. A. CAR T Cells Produced in Vivo to Treat Cardiac Injury. Science 2022, 375 (6576), 91–96. 10.1126/science.abm0594. 34990237 PMC9983611

[ref14] Vandergriff A.; Huang K.; Shen D.; Hu S.; Hensley M. T.; Caranasos T. G.; Qian L.; Cheng K. Targeting Regenerative Exosomes to Myocardial Infarction Using Cardiac Homing Peptide. Theranostics 2018, 8 (7), 1869–1878. 10.7150/thno.20524. 29556361 PMC5858505

[ref15] de Lima P. H. C.; Butera A. P.; Cabeça L. F.; Ribeiro-Viana R. M. Liposome Surface Modification by Phospholipid Chemical Reactions. Chem. Phys. Lipids 2021, 237, 105084 10.1016/j.chemphyslip.2021.105084. 33891960

[ref16] Gai M.; Simon J.; Lieberwirth I.; Mailänder V.; Morsbach S.; Landfester K. A Bio-Orthogonal Functionalization Strategy for Site-Specific Coupling of Antibodies on Vesicle Surfaces after Self-Assembly. Polym. Chem. 2020, 11 (2), 527–540. 10.1039/C9PY01136F.

[ref17] Armstrong J. P. K.; Shakur R.; Horne J. P.; Dickinson S. C.; Armstrong C. T.; Lau K.; Kadiwala J.; Lowe R.; Seddon A.; Mann S.; Anderson J. L. R.; Perriman A. W.; Hollander A. P. Artificial Membrane-Binding Proteins Stimulate Oxygenation of Stem Cells during Engineering of Large Cartilage Tissue. Nat. Commun. 2015, 6, 7405 10.1038/ncomms8405. 26080734 PMC4557285

[ref18] Delint R. C.; Day G. J.; Macalester W. J. P.; Kafienah W.; Xiao W.; Perriman A. W. An Artificial Membrane Binding Protein-Polymer Surfactant Nanocomplex Facilitates Stem Cell Adhesion to the Cartilage Extracellular Matrix. Biomaterials 2021, 276, 120996 10.1016/j.biomaterials.2021.120996. 34280823

[ref19] Dabbadie A.; Salerno A.; Perriman A.; Lian L. Y.; Hollander A. P. Development of Chimeric Forms of the Matrix Metalloproteinase 2 Collagen Binding Domain as Artificial Membrane Binding Proteins for Targeting Stem Cells to Cartilage Lesions in Osteoarthritic Joints. Biomaterials 2022, 285, 121547 10.1016/j.biomaterials.2022.121547. 35533445

[ref20] Burke M.; Armstrong J. P. K.; Goodwin A.; Deller R. C.; Carter B. M.; Harniman R. L.; Ginwalla A.; Ting V. P.; Davis S. A.; Perriman A. W. Regulation of Scaffold Cell Adhesion Using Artificial Membrane Binding Proteins. Macromol. Biosci. 2017, 17 (7), 1600523 10.1002/mabi.201600523. 28233419

[ref21] Deller R. C.; Richardson T.; Richardson R.; Bevan L.; Zampetakis I.; Scarpa F.; Perriman A. W. Artificial Cell Membrane Binding Thrombin Constructs Drive in Situ Fibrin Hydrogel Formation. Nat. Commun. 2019, 10 (1), 1887 10.1038/s41467-019-09763-0. 31015421 PMC6478844

[ref22] Xiao W.; Green T. I. P.; Liang X.; Delint R. C.; Perry G.; Roberts M. S.; Le Vay K.; Back C. R.; Ascione R.; Wang H.; Race P. R.; Perriman A. W. Designer Artificial Membrane Binding Proteins to Direct Stem Cells to the Myocardium. Chem. Sci. 2019, 10 (32), 7610–7618. 10.1039/C9SC02650A. 31588312 PMC6764276

[ref23] Zakeri B.; Fierer J. O.; Celik E.; Chittock E. C.; Schwarz-Linek U.; Moy V. T.; Howarth M. Peptide Tag Forming a Rapid Covalent Bond to a Protein, through Engineering a Bacterial Adhesin. Proc. Natl. Acad. Sci. U.S.A. 2012, 109 (12), E690–E697. 10.1073/pnas.1115485109. 22366317 PMC3311370

[ref24] Keeble A. H.; Banerjee A.; Ferla M. P.; Reddington S. C.; Anuar I. N. A. K.; Howarth M. Evolving Accelerated Amidation by SpyTag/SpyCatcher to Analyze Membrane Dynamics. Angew. Chem., Int. Ed. 2017, 56 (52), 16521–16525. 10.1002/anie.201707623. PMC5814910 29024296

[ref25] Keeble A. H.; Turkki P.; Stokes S.; Anuar I. N. A. K.; Rahikainen R.; Hytönen V. P.; Howarth M. Approaching Infinite Affinity through Engineering of Peptide-Protein Interaction. Proc. Natl. Acad. Sci. U.S.A. 2019, 116 (52), 26523–26533. 10.1073/pnas.1909653116. 31822621 PMC6936558

[ref26] Back C. R.; Sztukowska M. N.; Till M.; Lamont R. J.; Jenkinson H. F.; Nobbs A. H.; Race P. R. The *Streptococcus gordonii* Adhesin CshA Protein Binds Host Fibronectin via a Catch-Clamp Mechanism. J. Biol. Chem. 2017, 292 (5), 1538–1549. 10.1074/jbc.M116.760975. 27920201 PMC5290933

[ref27] Nicholls S. B.; Hardy J. A. Structural Basis of Fluorescence Quenching in Caspase Activatable-GFP. Protein Sci. 2013, 22 (3), 247–257. 10.1002/pro.2188. 23139158 PMC3595455

[ref28] Rana M. S.; Wang X.; Banerjee A. An Improved Strategy for Fluorescent Tagging of Membrane Proteins for Overexpression and Purification in Mammalian Cells. Biochemistry 2018, 57 (49), 6741–6751. 10.1021/acs.biochem.8b01070. 30481009 PMC7266526

[ref29] Lawrence M. S.; Phillips K. J.; Liu D. R. Supercharging Proteins Can Impart Unusual Resilience. J. Am. Chem. Soc. 2007, 129 (33), 10110–10112. 10.1021/ja071641y. 17665911 PMC2820565

[ref30] Shaner N. C.; Campbell R. E.; Steinbach P. A.; Giepmans B. N. G.; Palmer A. E.; Tsien R. Y. Improved Monomeric Red, Orange and Yellow Fluorescent Proteins Derived from Discosoma Sp. Red Fluorescent Protein. Nat. Biotechnol. 2004, 22 (12), 1567–1572. 10.1038/nbt1037. 15558047

[ref31] Day G. J.; Zhang W. H.; Carter B. M.; Xiao W.; Sambrook M. R.; Perriman A. W. A Rationally Designed Supercharged Protein-Enzyme Chimera Self-Assembles in Situ to Yield Bifunctional Composite Textiles. ACS Appl. Mater. Interfaces 2021, 13 (50), 60433–60445. 10.1021/acsami.1c18857. 34894651

[ref32] Zhang Y. I-TASSER Server for Protein 3D Structure Prediction. BMC Bioinf. 2008, 9 (1), 40 10.1186/1471-2105-9-40. PMC2245901 18215316

[ref33] Roy A.; Kucukural A.; Zhang Y. I-TASSER: A Unified Platform for Automated Protein Structure and Function Prediction. Nat. Protoc. 2010, 5 (4), 725–738. 10.1038/nprot.2010.5. 20360767 PMC2849174

[ref34] Yang J.; Yan R.; Roy A.; Xu D.; Poisson J.; Zhang Y. The I-TASSER Suite: Protein Structure and Function Prediction. Nat. Methods 2015, 12 (1), 7–8. 10.1038/nmeth.3213. PMC4428668 25549265

[ref35] Balu R.; Knott R.; Cowieson N. P.; Elvin C. M.; Hill A. J.; Choudhury N. R.; Dutta N. K. Structural Ensembles Reveal Intrinsic Disorder for the Multi-Stimuli Responsive Bio-Mimetic Protein Rec1-Resilin. Sci. Rep. 2015, 5, 10896 10.1038/srep10896. 26042819 PMC4455251

[ref36] Tedeschi G.; Salladini E.; Santambrogio C.; Grandori R.; Longhi S.; Brocca S. Conformational Response to Charge Clustering in Synthetic Intrinsically Disordered Proteins. Biochim. Biophys. Acta, Gen. Subj. 2018, 1862 (10), 2204–2214. 10.1016/j.bbagen.2018.07.011. 30025858

[ref37] Nayar R.; Hope M. J.; Cullis P. R. Generation of Large Unilamellar Vesicles from Long-Chain Saturated Phosphatidylcholines by Extrusion Technique. Biochim. Biophys. Acta, Biomembr. 1989, 986 (2), 200–206. 10.1016/0005-2736(89)90468-9.

[ref38] Skotland T.; Sandvig K.; Llorente A. Lipids in Exosomes: Current Knowledge and the Way Forward. Prog. Lipid Res. 2017, 66, 30–41. 10.1016/j.plipres.2017.03.001. 28342835

[ref39] Li K.; Chang S.; Wang Z.; Zhao X.; Chen D. A Novel Micro-Emulsion and Micelle Assembling Method to Prepare DEC205 Monoclonal Antibody Coupled Cationic Nanoliposomes for Simulating Exosomes to Target Dendritic Cells. Int. J. Pharm. 2015, 491 (1–2), 105–112. 10.1016/j.ijpharm.2015.05.068. 26073939

[ref40] van den Brink-van der Laan E.; Killian J. A.; De Kruijff B. Nonbilayer Lipids Affect Peripheral and Integral Membrane Proteins via Changes in the Lateral Pressure Profile. Biochim. Biophys. Acta, Biomembr. 2004, 1666 (1–2), 275–288. 10.1016/j.bbamem.2004.06.010. 15519321

[ref41] Stubbs C. D.; Slater S. J. The Effects of Non-Lamellar Forming Lipids on Membrane Protein-Lipid Interactions. Chem. Phys. Lipids 1996, 81 (2), 185–195. 10.1016/0009-3084(96)02581-9. 8810048

[ref42] Larsen J.; Hatzakis N. S.; Stamou D. Observation of Inhomogeneity in the Lipid Composition of Individual Nanoscale Liposomes. J. Am. Chem. Soc. 2011, 133 (28), 10685–10687. 10.1021/ja203984j. 21688773

[ref43] Humeres C.; Frangogiannis N. G. Fibroblasts in the Infarcted, Remodeling, and Failing Heart. JACC: Basic Transl. Sci. 2019, 4, 449–467. 10.1016/j.jacbts.2019.02.006. 31312768 PMC6610002

[ref44] Alexakis C.; Partridge T.; Bou-Gharios G. Implication of the Satellite Cell in Dystrophic Muscle Fibrosis: A Self-Perpetuating Mechanism of Collagen Overproduction. Am. J. Physiol.: Cell Physiol. 2007, 293, 661–669. 10.1152/ajpcell.00061.2007. 17475662

[ref45] Amelung S.; Nerlich A.; Rohde M.; Spellerberg B.; Cole J. N.; Nizet V.; Chhatwal G. S.; Talay S. R. The FbaB-Type Fibronectin-Binding Protein of *Streptococcus pyogenes* Promotes Specific Invasion into Endothelial Cells. Cell. Microbiol. 2011, 13 (8), 1200–1211. 10.1111/j.1462-5822.2011.01610.x. 21615663 PMC4754676

[ref46] Vitiello L.; Bockhold K.; Joshi P. B.; Worton R. G. Transfection of Cultured Myoblasts in High Serum Concentration with DODAC:DOPE Liposomes. Gene Ther. 1998, 5 (10), 1306–1313. 10.1038/sj.gt.3300729. 9930335

[ref47] Cruz-Samperio R.; Jordan M.; Perriman A. Cell Augmentation Strategies for Cardiac Stem Cell Therapies. Stem Cells Transl. Med. 2021, 10 (6), 855–866. 10.1002/sctm.20-0489. 33660953 PMC8133336

[ref48] Henderson B.; Nair S.; Pallas J.; Williams M. A. Fibronectin: A Multidomain Host Adhesin Targeted by Bacterial Fibronectin-Binding Proteins. FEMS Microbiol. Rev. 2011, 35 (1), 147–200. 10.1111/j.1574-6976.2010.00243.x. 20695902

[ref49] Wirth F.; Lubosch A.; Hamelmann S.; Nakchbandi I. A. Fibronectin and Its Receptors in Hematopoiesis. Cells 2020, 9, 2717 10.3390/cells9122717. 33353083 PMC7765895

[ref50] Sieber S.; Grossen P.; Detampel P.; Siegfried S.; Witzigmann D.; Huwyler J. Zebrafish as an Early Stage Screening Tool to Study the Systemic Circulation of Nanoparticulate Drug Delivery Systems in Vivo. J. Controlled Release 2017, 264, 180–191. 10.1016/j.jconrel.2017.08.023. 28851572

[ref51] Scott A.; Ballesteros L. S.; Bradshaw M.; Tsuji C.; Power A.; Lorriman J.; Love J.; Paul D.; Herman A.; Emanueli C.; Richardson R. J. In Vivo Characterization of Endogenous Cardiovascular Extracellular Vesicles in Larval and Adult Zebrafish. Arterioscler., Thromb., Vasc. Biol. 2021, 41 (9), 2454–2468. 10.1161/ATVBAHA.121.316539. 34261327 PMC8384253

[ref52] Sieber S.; Grossen P.; Uhl P.; Detampel P.; Mier W.; Witzigmann D.; Huwyler J. Zebrafish as a Predictive Screening Model to Assess Macrophage Clearance of Liposomes in Vivo. Nanomedicine 2019, 17, 82–93. 10.1016/j.nano.2018.11.017. 30659929

