# Supplementary material for: Modular Bioorthogonal Lipid Nanoparticle Modification Platforms for Cardiac Homing
Source: J Am Chem Soc. 2023 Oct 9;145(41):22659–70. doi: 10.1021/jacs.3c07811 (PMC10591475; doi:10.1021/jacs.3c07811)
Supplement: Supplementary file 20 — ja3c07811_si_020.pdf [file ja3c07811_si_020.pdf]

Supplementary Information

A modular bioorthogonal lipid nanoparticle modification platform for cardiac homing.

Raquel Cruz-Samperio,[1‡] Corrigan L. Hicks,[1‡] Aaron Scott,[2] Ignacio Gispert Contamina,[3] Yuval Elani,[3] Rebecca J. Richardson[2] and Adam W. Perriman.[1*]

Table of Contents

Materials and Methods

Supplementary Tables 1-7

Supplementary Equations 1-6

Supplementary Figures 1-27

Supplementary Movies 1-19

Materials and Methods

1. Plasmid production

Gene sequence encoding for protein fusion **1** (Table S1) flanked by a 20-base pair (bp) overhang matching the ends of a pET45b vector was ordered from Eurofins Genomics (Germany) for Gibson Assembly. The vector and the synthetic gene (1 µL) were amplified by two separate polymerase chain reactions (PCR) using PrimeSTAR master mix (25 µL, Takara Bio Europe), DMSO (dimethyl sulfoxide, 2 µL) and the primers from Table S2 (0.5 µL each) to a final reaction volume of 50 µL. The PCR reaction was performed in a thermocycler following a standard protocol: initial denaturation at 98 °C for 10 seconds, then 33 cycles of 98 °C for 10 seconds, 62 °C for 10 seconds, 72 °C for 60 seconds/1000 bp, and a final extension at 72 °C for 5 minutes. The amplified DNA fragments were purified by agarose gel electrophoresis (1% agarose gel, 5 µL SYBR™ safe dye), excised under blue light and purified using a GenElute™ PCR Clean-Up kit following the manufacturer protocol (Sigma-Aldrich, UK). Purified fragments were ligated using a Gibson Assembly master mix (10 µL, ThermoFisher Scientific, UK) mixed in a 1:3 ratio of gene to vector to a final volume of 20 µL, and the reaction was carried out at 50°C for 1 hour. Final product (5 µL) was transformed in DH5α cells (New England BioLabs, UK) for plasmid amplification with the GenElute™ Plasmid Miniprep kit (Sigma-Aldrich, UK) following the manufacturers protocols. The resulting plasmid was sequenced by Eurofins Genomics (UK) from the T7 promoter and T7 terminator sequences to confirm the success of the process.

Gene sequence encoding for protein fusion **3** (Table S1) was amplified from a plasmid containing mCherry-ST1 in a pOPIN F vector (produced by Dr. Wenjin Xiao, Perriman group) by PCR following the previous protocol, using the primers denoted as 'ST3 RGVP' (Table S2) to introduce the necessary mutations to the beginning of the ST1 peptide. The PCR product was purified following the same protocol as above, and the resulting linear plasmid (10 µL) was re-circularised using Gibson Assembly master mix (10 µL) and amplified following the same protocols as above. The resulting plasmid was sequenced by Eurofins Genomics (UK) from the T7 terminator sequence to confirm the success of the process and was then subjected to another PCR to incorporate the desired mutations at the end of the ST1 peptide and to produce overhangs to ligate the product to a pET45b vector using the primers of Table S2 and following the same protocols for the PCR, DNA purification, Gibson Assembly ligation and primer amplification as described in the above paragraph. The resulting plasmid was sequenced by Eurofins Genomics (UK) from the T7 promoter and T7 terminator sequences to confirm the success of the process.

Gene sequence encoding for protein fusion **2** (Table S1) in a pET45b vector was performed by incorporating the non-repetitive domains (NR1-3) of CshA in the N-terminal of the gene sequence encoding for protein fusion **3**. A gene encoding CshA-scGFP (produced by Dr. James Armstrong, Perriman group) was used to incorporate a 20-bp overhang using the sequence of a TEV cleavage site in the N-terminal, and a 20-bp overhang matching the mCherry sequence. The same sequences were incorporated into the plasmid containing protein fusion **3**. Both gene sequences were amplified by PCRs using the primers of Table S2, purified and ligated following the protocols described above. The resulting plasmid was transformed into DH5α cells and amplified following the protocols described above, and it was sequenced by Eurofins Genomics (UK) from the T7 promoter and T7 terminator sequences to confirm the success of the process.

2. Protein expression

Plasmids encoding for proteins **1** to **5** (50-100 ng) were transformed in BL21 (DE3) cells (50µL, New England BioLabs, UK) following the manufacturer guidelines, were plated in LB (lysogeny broth) agar cultures (1% glucose, 50 µg/mL carbenicillin) and incubated overnight at 37°C. Single colonies were inoculated in 10 mL of TB (terrific broth, 1% glucose, 50 µg/mL carbenicillin) and were grown in a rotating incubator (180 RPM) at 37°C overnight. 5 mL of the overnight cultures were inoculated in 1 L TB (50 µg/mL carbenicillin, TUNAIR™ flasks) and were grown in a rotating incubator (180 RPM) at 37°C until the optical density (OD) of the culture reached 1.2 at 600 nm using an UV-vis light spectrometer (Agilent technologies, UK). Protein production was induced by addition of 1 mM IPTG (isopropylthiogalactosidase, Apollo Scientific, Japan) and cultures were incubated at 20°C overnight at 180 RPM. Cultures were harvested by centrifugation at 4000 g for 20 minutes at 4°C using a Sorvall RC6 centrifuge (ThermoFisher Scientific, UK). Pellets were stored at -80°C for future use.

3. Protein purification

A cell pellet was resuspended in 40 mL lysis buffer (**1**, **4**: 20 mM PBS (phosphate buffered saline), 2 M NaCl, 10 mM imidazole, pH 7.5, **2, 3, 5**: 20 mM PBS, 300 mM NaCl, 10 mM imidazole, pH 7.5), supplemented with 100 µL PMSF (phenylymethylsulfonyl fluoride, 100 mM in DMSO, ThermoFisher Scientific, UK), 20 mg lysozyme, and DNase (deoxyribonuclease I from bovine pancreas, Sigma Aldrich, UK). The cells were then lysed using a Vibra Cell VCX 1500 ultrasonic processor (Sonics & Materials, USA) at an amplitude of 40% for 5 minutes (5 s on, 10 s

off) in an ice bath. The cell lysate was centrifuged at 18,000 g for 45 minutes using an Avanti J 25 centrifuge (Beckamn Coulter, USA) to remove any cell debris, and filtered using a 0.45 μm cellulose membrane syringe filter. The clarified cell lysate was loaded into a HisTrap FF column (GE Healthcare Life Sciences, UK) containing Ni Sepharose 6 Fast Flow pre-equilibrated with lysis buffer. The column was washed with lysis buffer using a NGC Quest™ 10 Plus chromatography system (Bio-Rad Laboratories Ltd., UK). The bound protein was eluted in 10 mL elution buffer (**1, 4**: 20 mM PBS, 2 M NaCl, 500 mM imidazole, pH 7.5, **2, 3, 5**: 20 mM PBS, 300 mM NaCl, 500 mM imidazole, pH 7.5) in reverse flow directly onto a Size Exclusion Chromatography column (HiLoad 26/600 Superdex 200 pg, Cytiva, UK) pre-equilibrated into SEC buffer (**1, 4**: 20 mM PBS, 500 mM NaCl, pH 7.5, **2-5**: 20 mM PBS, 300 mM NaCl, pH 7.5). Proteins were monitored at 280 nm, and 487 nm or 585 nm for GFP or mCherry containing proteins respectively, 5 mL fractions were collected, and the protein fractions were pooled and concentrated in spin concentrators (Sartorius, VivaSpin 30,000 MWCO).

Proteins **1** and **2** were mix with aliquots of Tobacco-Etch Virus (TEV) protease in a 10:1 protein concentration ratio to cleave the N-terminal polyhistidine tags and the mixture was allowed to incubate overnight at 4°C. The mixture was loaded into a HisTrap FF column and washed in SEC buffer to collect the cleaved products. Proteins were dialysed into assay buffer (**1**: 20 mM Tris-HCl, 500 mM NaCl, pH 7.5; 2-5: 20 mM Tris-HCl, pH 7.5) for the SpyCatcher-SpyTag reaction assays or into cell assay buffer (**1**: PBS, 500 mM NaCl, pH 7.5, **2-5**: PBS pH 7.5) for *in vitro* and *in vivo* assays, their concentration was determined by BCA (bicinchoninic acid), flash-freezed into 10 M aliquots and stored at -80 for further use.

4. Sodium dodecyl sulphate-polyacrylamide gel electrophoresis (SDS-PAGE)

20 μL sample was added to 5 μL of sample application buffer comprised (400 mM glycerol, 5% SDS, 50 mM EDTA (ethylenediaminetetraacetic acid), 0.5 mM Tris-HCl, 7 mM β-mercaptoethanol and bromophenol blue), and denatured by heating at 95 °C for 5 minutes in a Grant QBD2 block heater (Wolf Laboratories, UK). 5-10 μL of this mixture was applied to a Novex® 4-20% Tris-Glycine precast protein gel (ThermoFisher Scientific, UK), using BLUeye Prestained Protein Ladder (Sigma Aldrick, UK) as a reference. The gel was run into a Mini Gel Tank (Thermo Fisher Scientific, UK) submerged in running buffer (50 mM Tris base, 38.5 mM glycine, 7 mM SDS) at a voltage of 180 V for 1 hour before staining with Coomassie Blue™ protein stain for 20 minutes at room temperature. The gel was destained by shaking overnight in deionized water at room temperature to reveal the protein bands.

5. Bicinchoninic acid (BCA) assay

A Pierce BCA Protein Assay kit (Thermo Fisher Scientific, UK) was used to determine the protein concentration. Working reagent (WR) was prepared by mixing reagent A (containing sodium carbonate, sodium bicarbonate, bicinchoninic acid and sodium tartrate in 0.1M sodium hydroxide) and reagent B (containing 4% cupric sulfate) in a 50:1 (v/v) ratio. A 25 μL aliquot of protein was added to a clear 96 well plate (Corning, USA) in triplicate, followed by the addition of 200 μL WR. Triplicate 25μL aliquots of bovine serum albumin (BSA) (Sigma Aldrich, UK) of known concentrations were used as controls for a standard curve. The plate was incubated at 37°C for 30 minutes and left to cool for 5 minutes before the absorbance at 562 nm was measured using a Synergy Neo2 multi-mode plate reader (BioTek, USA). The concentration of protein was calculated using the standard curve.

6. Oxidation of the polymer surfactant

The oxidised IGEPAL co-890 surfactant, [S], was synthesised following the protocol outlined by Armstrong *et al.*[1] 2 g IGEPAL co-890 dissolved in 50 mL deionised water was mixed with 50 mg sodium bromide (NaBr), 30 mg of TEMPO ((2,2,6,6 Tetramethylpiperidin-1-yl)oxyl), and 5 mL of a sodium hypochlorite (NaClO) solution containing 10-15% available chlorine. The solution was adjusted to pH 11 using NaOH and stirred for 24 hours at room temperature. 10 mL of ethanol was added to quench the reaction and the pH adjusted to 1 with HCl. The oxidised surfactant was purified through chloroform solvent extraction (3x80 mL), followed by 3 washes of 80 mL deionised water (pH = 1). The chloroform was removed under reduced pressure at 40 °C and the resulting solid dissolved in 40 mL of ethanol at 65 °C and recrystallised overnight at -20 °C. The ethanol was decanted and the solid product dried under reduced pressure at 65 °C. Fourier-transform infrared spectroscopy (FTIR, Perkin Elmer, USA), MALDI-TOF mass spectrometry, and an acid-base titration were performed to confirm successful oxidation.

7. Synthesis of SC3-scGFP/polymer surfactant complex

To form the AMBPs, the protein concentration was measured using UV-Vis spectroscopy or a BCA assay. The number of positive sites (arginine and lysine residues) on the purified protein is 60 for protein **1**, 131 for **4** and 44 for **5**. The surfactant (25 mg/mL in 20 mM phosphate buffer, pH 7.5) was added to the proteins in stoichiometric excess of 1.2 for **1** and **4**, and 1.4 for **5**, and 1.2 for [CshA-scGFP] and [SC3-scGFP]. The solution

was stirred overnight at 4 °C at 150 RPM before being dialysed into the appropriate buffer using 14000 MWCO cellulose dialysis tubing to remove excess surfactant which was not conjugated.

8. UV-Vis spectroscopy

A Cary 60 UV-Vis spectrometer (Agilent Technologies, USA) was used to measure the absorbance between λ = 200 and 800 nm. OD of cultures was measured in plastic cuvettes at 600 nm. Purified proteins were measure in quartz cuvettes (1 cm pathlength). Concentration (C) was calculated using the Beer-Lambert law.

9. Dynamic Light Scattering (DLS)

Samples for DLS characterisation were filtered through a 0.22 µm syringe filter into a semi-micro volume disposable cuvette (Sigma Aldrich, UK) and data collected using a ZetaSizer Nano ZS (Malvern Instruments, UK). Samples were incubated at 25 °C for 120 seconds and three measurements were taken for each sample. The intensity and number percentage distributions data were plotted as a frequency distribution along with a Gaussian distribution curve, fitted using Prism™ 8 (GraphPad Software, USA).

10. Circular Dichroism (CD) spectroscopy

Proteins were dialysed into CD buffer (10 mM potassium phosphate, 50 mM sodium sulphate at pH 8.0) overnight at a higher concentration than required for measurements. This was then diluted to a suitable concentration using degassed CD buffer and filtered through a 0.22 µm syringe filter before being transferred into a quartz cuvette. The sample was equilibrated at 25 °C for 5 minutes in a J-1500 CD spectrometer (Jasco) before data was collected from 260 to 190 nm (5 nm/min scanning spin) in triplicate. For full spectrum temperature unfolding scans, a single scan was taken from 260 to 190 nm (5 nm/min scanning spin) every 5 °C between 25 – 95 °C.

11. Fluorescence spectroscopy

Samples were analysed using a Cary Eclipse fluorescence spectrometer and Cary Scan software (Both Agilent Technologies, USA). Both excitation and emission spectra of proteins were performed from 350 nm to 600 nm at room temperature.

12. SpyCatcher-SpyTag reaction

Reactions between protein fusion **1** to **2** or **3** were carried out to analyse the success of isopeptide bond formation despite the fusion proteins. Reactions were carried out in 20 mM Tris-HCl buffer at pH 7.0 at either 37 °C or 4 °C, with protein concentrations of either 1 µM or 10 µM. All reactions were carried out in triplicate in a reaction volume of 250 µL and 20 µL aliquot of the reaction mixture was taken at different time points, quenched with 5 µL SDS loading buffer, boiled at 95 °C for 5 minutes and were analysed by SDS-PAGE. The reaction aliquots and starting reagents were applied to a TruPAGE 10% gel (Sigma Aldrich, UK) using BLUeye Prestained Protein Ladder ladder (10 – 245 kDa) (Sigma Aldrich, UK) as a reference. The gel was placed into a Mini Gel Tank (Thermo Fisher Scientific, UK) submerged in running buffer, and a voltage of 180 V applied for 45 minutes before staining with Coomassie Blue™ protein stain for 10 minutes at room temperature. The gel was de-stained by shaking overnight with deionised water at room temperature to reveal protein bands. Gels were imaged using a BioDoc It®2 imager (Wolf Laboratories, UK) and analysed using GelAnalyzer software (GelAnalyzer 19.1 (www.gelanalyzer.com) by Istvan Lazar Jr., PhD and Istvan Lazar Sr., PhD, CSc. Relative pixel intensity for each molecule was calculated from normalizing the pixel intensity of each species from the total pixel intensity of each lane. Data was plotted as the mean ± SD was plotted for the reaction substrates and the final products using Prism™ 8 (GraphPad Software, USA). The yield of each reaction reported in the main text was stimated based on the decrease of concentration of substrate **1** (SC3-scGFP).

13. Synchrotron radiation small angle X-ray scattering (SR-SAXS)

Sample measurements (5 mg/mL) were carried out on beamline B21 by beamline scientists as Diamond Light Source, Didcot, through their mail-in service. All samples were subjected to size exclusion chromatography before scattering (SEC SAXS) via a superdex 200 column (GE Healthcare, US). The data was buffer subtracted and analysed using ScÅtter IV software developed at the Diamond Light Source (Harwell Science and Innovation Campus, UK) and SIBYLS Beamline at LBNL (Berkeley CA). The SAXS fits and P(r) distribution files (.out) produced from ScÅtter were submitted to EMBL DAMMIF online service37 to obtain a dummy atom bead model for the proteins. The DAMMIF service performed 20 reconstructions with P1 symmetry, the volumes of all runs were combined to produce the damaver file. Subsequently, this averaged model was refined to produce the filtered average damfilt file. The final models were visualised in the PyMol software package (Schrödinger, USA), overlayed with the specified crystal structures obtained from the Protein Database (PDB).

14. Synthesis of Lipid Nanoparticles (LNPs)

1,2-dioleoyl-sn-glycero-3-phosphocholine (DOPC), 1,2-dioleoyl-sn-glycero-3-phosphoethanolamine (DOPE), 1,2-dioleoyl-sn-glycero-3-phospho-L-serine (sodium salt) (DOPS), and cholesterol (ovine wool, > 98%) (CHOL) were purchased from Avanti Polar Lipids, USA. The fluorescent lipid Texas Red™ 1,2-Dihexadecanoyl-sn-Glycero-3-Phosphoethanolamine, Triethylammonium Salt (Texas Red™ DHPE) (TR) was purchased from Thermo Fisher Scientific, UK. Chloroform (Sigma Aldrich, UK) was used to prepare the lipid stock solutions of 1 mg/mL concentration.

LNPs were formed from CHOL, DOPC, DOPS and DOPE in a 55:21:16:8 % ratio, and if required for imaging or fluorescence measurements, Texas Red™ DHPE (TR) was added at either 1 or 5 vol % to DOPC (0.7 or 3.8 mol % respectively), using a standard extrusion protocol.[2] The desired volume of a 1mM lipid in chloroform solution was added to a glass vial, the chloroform was evaporated under a nitrogen stream whilst the vial was rotated, producing a thin lipid film. This was placed in a vacuum desiccator for 2 hours to remove any residual chloroform. The lipid film was hydrated with PBS (10% volume of the original lipid in chloroform solution, or 100% for 10 times concentrated LNPs) for at least 30 minutes. LNPs encapsulating mCherry were hydrated with 10 μM mCherry in PBS instead. The lipid suspension was then agitated by vortexing for around 30 seconds before it was subjected to five freeze thaw cycles in which the suspension was placed alternately in liquid nitrogen, and a warm water bath, with vortexing in between. The suspension was then loaded into a pre-wetted gas tight syringe (Avanti ® Polar Lipids, USA) and placed into one end of the mini-extruder (Avanti ® Polar Lipids, USA) containing a polycarbonate filter with a pore size of 100 nm. The lipid solution was passed into the second syringe and back to the original syringe 10 times, with the final vesicle solution ending up in the second syringe. LNPs containing mCherry were transferred to a 100 kDa MWCO spin concentrator Sartorius, Germany) and buffer exchanged three times with PBS to remove any excess mCherry. The LNPs produced were either used immediately or stored at 4 °C.

15. Synthesis of Giant Unilamellar Vesicles (GUVs)

GUVs were formed from CHOL, DOPC, DOPS and DOPE in a 55:21:16:8 % ratio, and if required for imaging or fluorescence measurements, Texas Red™ DHPE (TR) was added at either 1 or 5 vol % to DOPC (0.7 or 3.8 mol % respectively). GUVs were formed by electroformation following an adapted protocol outlined by Li *et al.*,[3] in which 50 μL of the lipid mixture was deposited onto an ITO slide and left for 20 seconds before smearing with the corner of a coverslip to create a thin lipid film. This was placed in a vacuum desiccator for 15 minutes at room temperature to ensure removal of all chloroform. A polydimethylsiloxane (PDMS) spacer was placed over the film, closed with a second ITO slide, and held in place with bulldog clips. A 0.5 M sucrose solution in either water or PBS was injected into the well ensuring no air remained. The GUV set up was connected to a function generator and an alternating electric field was applied at 1 V, 10 Hz for 2 hours at room temperature, before changing to 1 V and 2 Hz for 1 hour. Once formed the GUVs were collected in an Eppendorf tube and used immediately or stored at 4 °C for up to 3 days.

16. AMBP modification of LNPs

The desired protein in 10 μM concentration was added to the LNP suspension in a 10% (v/v) ratio and incubated at 4 °C between 30 minutes and 24 hours. If required, the LNP protein solution was transferred to a 100 kDa or 300 kDa MWCO spin concentrator (Sartorius, Germany) and buffer exchanged three times with PBS to remove any excess, unbound protein.

17. AMBP modification of GUVs

The desired protein was added to the GUV suspension and either imaged immediately or incubated at 4 °C overnight before imaging. If required, excess, unbound protein was removed via gentle dialysis for between 6 – 24 hours using a Float-A-Lyzer™ G2 dialysis device (Spectrum Chemical Manufacturing Corp, USA) with a 100 kDa MWCO.

18. Nanoparticle tracking analysis (ZetaView®)

LPNs were diluted to a suitable concentration in the range of 105 to 107 vesicles per mL and injected into the measuring cell. Measurements were taken at 11 different positions across the cell and results were based on the tracking of thousands of individual particles. A 488 nm laser was used to excited GFP for detection of modified LNPs.

19. Cryo-EM of LNP samples

LPN suspensions (approximately 1.5 x 10^{13} LNPs/mL) in 20 mM HEPES buffer (pH 7.0) were placed on glow discharged lacey carbon films supported by 300 mesh copper grids (EMResolutions) and vitrification of was

performed using a Leica EM GP operating at a 30 second pre-blot and 1.2 second blot time. After vitrification, sample grids were maintained below -170°C and imaging was performed on a Tecnai T12 with a 120 kV BioTwin Spirit objective lens and tungsten filament equipped with a FEI Ceta 4K x 4K CCD camera. Images were processed and the LNP size and membrane thickness were determined using the Fiji software.[4] Only distinct LNPs were characterised, and data was plotted and analysed with GraphPad Prism.

20. C2C12 myoblast culture
All cell culture work was carried out in SAFE 2020 laminar flow hoods (Thermo Fisher Scientific, UK) and incubated in a Culture Safe Precision P190D incubator (LEEC, UK) at 37 °C under a 5 % carbon dioxide (CO2) atmosphere. C2C12 cell line (C3H muscle mouse myoblasts) was purchased from Merck, UK. For optimal cell growth and viability, C2C12 cells were cultured in Dulbecco's Modified Eagle's Medium (DMEM) high-glucose medium (Merck, UK) supplemented with 10 % (v/v) fetal bovine serum (FBS), and 1 % (v/v) penicillin/streptomycin. The media was changed every 2 days and cells were passaged once reached 70% confluency with trypsin/EDTA solution (Sigma Aldrich, UK) following the manufacturer guidelines.

21. Static experiments
250'000 C2C12 fibroblasts were seeded in either tissue culture treated 35 mm confocal dishes (VWR ®, USA) or 24 well plates with culture media 24 hours prior to the experiments. The following day 100 µL of sample, either 1 µM protein or approximately 1.5 x 10^{11} native or modified LNPs, was added to the cells and incubated for 2 hours at 37 °C in a 5 % CO_2 atmosphere. Cells were washed three times with 1 mL PBS, and were either supplemented with 2 mL high-glucose DMEM medium for microscopy studies, or detached using trypsin/EDTA and resuspended in PBS for flow-cytometry studies.

22. Flow experiments
250'000 C2C12 cells were seeded in a 0.4 µ-Slide I Luer glass bottom channel slide (Ibidi, Germany) 24 hours prior to the experiment. The channel was connected to a syringe pump on one end, and to waste on the other, and the flow rate was adjusted depending on the syringe volume to apply 2 dynes stress shear to the channel during the experiment. The nuclei were stained with 10 µg·mL^{-1} Hoechst 33342 (Thermo Fisher Scientific, UK) in 10 mL DMEM. Protein modified LNPs (approximately 1.5 x 10^{11} in 25 mL DMEM) were passed through the channel and images and videos were recorded at time 0, after 30 minutes, and 1 hour using a Leica DMI6000 inverted epifluorescence microscope. Cells were detached using trypsin/EDTA and resuspended in PBS at the end of the microscopy experiment for flow-cytometry studies.

23. Confocal microscopy
Confocal microscopy was performed at the Wolfson Bioimaging Facility within the Faculty of Life Sciences at the University of Bristol. Samples were placed into a 35 mm confocal dish (VWR ®, USA) and mounted on to a Leica SP8 AOBS confocal laser scanning microscope attached to a Leica DMi8 inverted epifluorescence microscope (Leica, UK) with a 63X oil immersion objective. Three dimensional stacked images were obtained using a z-separation length of 0.3 µm. Videos of varying duration were taken with 15 s intervals between images. Images and videos were captured using Leica LAS-X acquisition software (Leica, UK) and processed using Fiji software.[4]

24. Widefield microscopy
Fluorescence microscopy was performed on a Leica DMI6000 at the Wolfson Bioimaging Facility within the Faculty of Life Sciences at the University of Bristol, or at the Molecular Sciences Research Hub, Imperial College London for the GUV experiments with the help of Dr Yuval Elani and Ignacio Gispert Contamina. Images and videos were recorded in green, red, and blue channels for imaging of GFP, mCherry or Texas Red, and Hoechst 33342 labelled samples respectively. Images were captured using Leica LAS-X acquisition software (Leica, UK) and processed using Fiji software.[4] For GUV phase contrast imaging, 10 µL of GUVs were added to 90 µL of a 0.5 M glucose solution on a microscope slide, using a PDMS spacer.

25. Alamar Blue Cytotoxicity Assay
An initial experiment was conducted to determine the optimal incubation period to determine at which time point there was significant reduction of AlamarBlue™. 50'000 C2C12 cells were seeded into 24 well plates and allowed to adhere overnight at 37 °C in a 5 % CO2 atmosphere. The following day, the media was replaced with 500 µL fresh media containing 10 % (v/v) AlamarBlue™ cell viability reagent and incubated for either 2, 4, 6, 8, or 24 hours at 37 °C in a 5 % CO2 atmosphere. It was considered that 6-hour incubation with the reagent was optimal. Either 1 µM protein or approximately 3 x 10^{10} native or modified LNPs were added to 50'000 C2C12 cells in 24 well plates, and incubated for 2 hours. All wells were washed three times with PBS before replacing with 500 µL fresh media containing 10 % (v/v) AlamarBlue™ and incubating for 6 hours before 100 µL media from each well was added to a 96 well untreated plate in triplicate. Fluorescence at excitation 560 nm and

emission 590 nm was measured using a Synergy Neo2 plate reader (BioTek, USA). The remaining AlamarBlue™ media was aspirated and all wells replenished with fresh media. This was repeated on days 1, 3 and 7. For each condition, one well was replenished with fresh media without AlamarBlue™ and used for live/dead imaging.

26. Flow cytometry studies

Flow cytometry studies were performed in the Flow Cytometry Facility (School of Cellular and Molecular Medicine, University of Bristol, UK) using a BD Fortessa X20 (BD Biosciences) cytometer, equipped with a 405, 488, 561 and 633 nm lasers. After incubation experiments, followed by DMEM or PBS washes, cells were detached using trypsin/EDTA (100 µL/well or flow slide) and resuspended in 200 µL PBS for analysis. Samples were vortexed briefly before measurement to resuspend any cell aggregates. C2C12 myoblast population was defined by forward scatter area (FSC-A) vs the side scatter area (SSC-A) and single cells were gated by FSC-A vs forward scatter height (FSC-H), with the assistance of Dr. Andrew Herman. GFP (FITC-A channel) and mCherry (PE-CF594-A channel) fluorescence were measured for 10’000 single cell events in triplicate for each static condition and once for the flow conditions. Each experiment was repeated at least 3 times for statistical analysis. Data was analysed using FlowJo v10 software to obtain the mean of fluorescence of each cell population.

27. Zebrafish experiments

2 day post-fertilisation larval zebrafish were anaesthetised in 1.3 mM tricaine mesylate (MS-222) and mounted in 1% low-gelling agarose (Sigma Aldrich, UK). 2 nL of sample, either 20 µM surfactant coated **1,** and proteins **2** and **3**, or 3 x 10^6 **1**-LNPs, **1:2**-LNPs and **1:3**-LNPs, were microinjected into the duct of Cuvier. Live imaging was performed on a Leica TCS SP8 AOBS confocal laser scanning microscope with a 25 x / 0.95 W HC FLUOTAR objective with resonant scanner. Frame intervals were 0.02 – 0.04 seconds.

28. Statistical analysis

GraphPad Prism was used to plot the different graphs in the paper and to perform the statistical analysis using ANOVA and a two-tailed Student’s t-test with p-value of less than 0.05 were considered significant (*). P-values of less than 0.01 were considered highly significant (** and more).

29. References

(1) Armstrong, J. P. K.; Shakur, R.; Horne, J. P.; Dickinson, S. C.; Armstrong, C. T.; Lau, K.; Kadiwala, J.; Lowe, R.; Seddon, A.; Mann, S.; Anderson, J. L. R.; Perriman, A. W.; Hollander, A. P. Artificial Membrane-Binding Proteins Stimulate Oxygenation of Stem Cells during Engineering of Large Cartilage Tissue. *Nat. Commun.* **2015**, *6*, 7405. https://doi.org/10.1038/ncomms8405.

(2) Nayar, R.; Hope, M. J.; Cullis, P. R. Generation of Large Unilamellar Vesicles from Long-Chain Saturated Phosphatidylcholines by Extrusion Technique. *BBA - Biomembr.* **1989**, *986* (2), 200–206. https://doi.org/10.1016/0005-2736(89)90468-9.

(3) Li, K.; Chang, S.; Wang, Z.; Zhao, X.; Chen, D. A Novel Micro-Emulsion and Micelle Assembling Method to Prepare DEC205 Monoclonal Antibody Coupled Cationic Nanoliposomes for Simulating Exosomes to Target Dendritic Cells. *Int. J. Pharm.* **2015**, *491* (1–2), 105–112. https://doi.org/10.1016/j.ijpharm.2015.05.068.

(4) Schindelin, J.; Arganda-Carreras, I.; Frise, E.; Kaynig, V.; Longair, M.; Pietzsch, T.; Preibisch, S.; Rueden, C.; Saalfeld, S.; Schmid, B.; Tinevez, J. Y.; White, D. J.; Hartenstein, V.; Eliceiri, K.; Tomancak, P.; Cardona, A. Fiji: An Open-Source Platform for Biological-Image Analysis. *Nat. Methods* **2012**, *9* (7), 676–682. https://doi.org/10.1038/nmeth.2019.

(5) Basham, M.; Filik, J.; Wharmby, M. T.; Chang, P. C. Y.; El Kassaby, B.; Gerring, M.; Aishima, J.; Levik, K.; Pulford, B. C. A.; Sikharulidze, I.; Sneddon, D.; Webber, M.; Dhesi, S. S.; Maccherozzi, F.; Svensson, O.; Brockhauser, S.; Náray, G.; Ashton, A. W. Data Analysis WorkbeNch (DAWN). *J. Synchrotron Radiat.* **2015**, *22*, 853–858. https://doi.org/10.1107/S1600577515002283.

Supplementary Tables

Table S1. Gene sequences of proteins of interest. All of the gene sequences were cloned into pET45b vectors.

Construct	Sequence 5'→3'
SC3-scGFP	ATG GCA CAC CAC CAT CAC CAT CAT TCC AGT GGT TTA GAG GTG TTG TTT CAG GGT CCA GAA AAT CTG<br>TAC TTC CAA GGC GCG ATG GTT ACC ACC CTG AGC GGG TTA AGC GGT GAG CAA GGC CCT AGT GGG GAT<br>ATG ACG ACG GAA GAG GAT TCG GCA ACG CAT ATC AAA TTT AGC AAG CGT GAC GAA GAT GGC CGT GAA<br>CTT GCG GGT GCA ACG ATG GAA CTT CGT GAT TCG TCA GGC AAA ACG ATC TCC ACC TGG ATT AGC GAT<br>GGG CAT GTT AAA GAC TTC TAC CTC TAT CCG GGC AAA TAT ACC TTT GTG GAA ACT GCG GCT CCT GAT<br>GGT TAT GAG GTT GCG ACG CCG ATT GAA TTT ACC GTG AAT GAA GAT GGC CAG GTC ACT GTG GAC GGT<br>GAA GCC ACT GAA GGT GAT GCG CAT ACA GGT GGT TCA GGT GGA ACA GGC GGA AGT GGT GGC ACC GGA<br>GGG TCA GGT GGC ACT GGG GCT TCG AAA GGA GAA CGG CTG TTT CGC GGC AAA GTG CCC ATT CTG GTT<br>GAA CTC AAA GGT GAC GTG AAT GGC CAC AAA TTC AGC GTA CGT GGC AAA GGC AAG GGC GAT GCT ACT<br>CGC GGC AAA CTG ACC CTG AAG TTC ATT TGT ACG ACT GGG AAA TTG CCA GTA CCG TGG CCA ACA CTC<br>GTG ACA ACC CTG ACC TAT GGC GTT CAG TGC TTT AGC CGC TAT CCC AAA CAC ATG AAG CGC CAT GAT<br>TTC TTC AAG TCT GCC ATG CCG AAA GGC TAT GTC CAG GAA CGG ACC ATC TCC TTT AAG AAA GAT GGC<br>AAG TAC AAA ACC CGT GCG GAA GTA AAA TTC GAA GGT CGC ACG CTG GTT AAC CGC ATC AAA CTG AAA<br>GGT CGT GAC TTT AAA GAG AAA GGG AAC ATT TTG GGT CAC AAA CTT CGC TAC AAC TTC AAC TCT CAC<br>AAA GTC TAC ATT ACC GCC GAT AAA CGC AAG AAC GGC ATT AAG GCC AAA TTT AAA ATC CGC CAT AAC<br>GTC AAA GAC GGC TCT GTG CAA CTG GCA GAT CAT TAC CAG CAG AAT ACC CCG ATT GGA CGT GGT CCG<br>GTC TTA CTG CCG CGT AAT CAC TAT CTG AGT ACC CGC AGC AAA CTG TCG AAA GAC CCG AAA GAG AAA<br>CGC GAC CAT ATG GTG TTA CTG GAG TTT GTA ACG GCC TAA
mCh-ST3	ATG GCA CAC CAT CAC CAC CAT CAC AGC AGC GGT CTG GAA GTT CTG TTT CAG GGC CCG ATG GTG AGC<br>AAG GGC GAG GAG GAT AAC ATG GCC ATC ATC AAG GAG TTC ATG CGC TTC AAG GTG CAC ATG GAG GGC<br>TCC GTG AAC GGC CAC GAG TTC GAG ATC GAG GGC GAG GGC GAG GGC CGC CCC TAC GAG GGC ACC CAG<br>ACC GCC AAG CTG AAG GTG ACC AAG GGT GGC CCC CTG CCC TTC GCC TGG GAC ATC CTG TCC CCT CAG<br>TTC ATG TAC GGC TCC AAG GCC TAC GTG AAG CAC CCC GCC GAC ATC CCC GAC TAC TTG AAG CTG TCC<br>TTC CCC GAG GGC TTC AAG TGG GAG CGC GTG ATG AAC TTC GAG GAC GGC GGC GTG GTG ACC GTG ACC<br>CAG GAC TCC TCC CTG CAG GAC GGC GAG TTC ATC TAC AAG GTG AAG CTG CGC GGC ACC AAC TTC CCC<br>TCC GAC GGC CCC GTA ATG CAG AAG AAG ACC ATG GGC TGG GAG GCC TCC TCC GAG CGG ATG TAC CCC<br>GAG GAC GGC GCC CTG AAG GGC GAG ATC AAG CAG AGG CTG AAG CTG AAG GAC GGC GGC CAC TAC GAC<br>GCT GAG GTC AAG ACC ACC TAC AAG GCC AAG AAG CCC GTG CAG CTG CCC GGC GCC TAC AAC GTC AAC<br>ATC AAG TTG GAC ATC ACC TCC CAC AAC GAG GAC TAC ACC ATC GTG GAA CAG TAC GAA CGC GCC GAG<br>GGC CGC CAC TCC ACC GGC GGC ATG GAC GAG CTG TAC AAG GGT GGT AGC GGT GGC ACC CGT GGT GTG<br>CCG CAT ATT GTT ATG GTT GAT GCA TAT AAA CGC TAT AAA TAA
CshA-mCh-ST3	ATG GCA CAC CAT CAC CAC CAT CAC AGC AGC GGT CTG GAA GTT CTG TTT CAG GGT CCG GAG AAT CTT<br>TAT TTT CAG GGC ATG GAT GAA ACA AGT GCG AGT GGG GTA CAG AAC GAA GTT GCT CGT GCC GAT TTG<br>GCG GAA TCC CCG GCT ACC GCC ACC GCG CCC GTC GCG TCT GAA GCC TCC CAG AAT GCG GAA ACC ACA<br>GTG GCT GCG ACC GCT ACT GAA GCC CCG CAG ACC GCG GAA AAC ACT GCT CCT ACC AAC AGT GCC AGT<br>GAA TCA ACC GAA AAA CCG ATG GAC GAA CAG CCT GTG GCT TCT GAA ACC CCA CAA CCA AGC GTC GAG<br>AAA CCG GTA TTA CCG ACC GAG GTG AAA CCG GCA GAG AAT ACA ACT CCG GCA AGT ACG GAG GCC AGT<br>CCG GAG ACA GTT TCT CCA TCG CGC GCG ACA GAC CAG CCT GTA GCC ACC CGT GAT TCC GTG CAG TCT<br>AGC CGT TCG CGC CGG TTG CGC CGT GAC CTG GAA GCT ACC GCT GTG ACG CCA GGT ACA GGT CCG GCT<br>GGT GCG GAT GAT GCA ACA CCG ATT CCT CGT GTC AGC AAA CCG ACC CTG TCA GAG TCA GAA AAA AAA<br>GAA TCC ACC CAA CTG GCG AAG CAG ATT AAC TGG GTA GAC TTC TCC GAC ACA GCG TCA ATG AAG AAT<br>TTA GAT CCG CAA GGA GGA TTC AAA GTA GGT ACC GTT TTC AAA AAG GAA ATC TCG CCG GGT TAT GTG<br>GTG ACG CTG ACC GTT ACT GAA CTG AAA CCC TTC AAC AGC ACC GAA ATT TAC AAG AAA CGC GTT GAA<br>GGG ACT CCC ACG GCA AAT ACC TAC GAC CCG AAC GCG ATC AAT AGC TAT CTG AAA GGC TAT AAA GAT<br>TAC GGT AAA ACC CCG CCG TCT GTT ACG GGC CGC CCG CAG AAC AAA TTT TCA ACC ATT GGC GGT CAG<br>GGG TTC GAT ACC CAA GGT CGT AAA ACG CAG ATC ATT TTG CCG GAC GAC GCG GTT AAT TGG GGC ATC<br>AAA TTT AAG GTG GAA GCA ACC TAT CGC GGA AAT CCT GTG AAA CCT TCG GTC GTT ATG GCC GAT GGG<br>GAA GAT GCC AAT CCG GCT GAA TAT GGG ATT TTC ACG ACT AAC GGT GAA GGG TGG GAG TAT GTG GGC<br>GAA TGG ATG AAA GGA CCC CGC GCG AAA GGC CCG TAC ACT GTG ATG ACT GAA GAT ATG GTG AAG GCA<br>TTC GAT AAA ACC CGC AAA GAC GGT CTG CTG ATC CTG AAA GAT AAA AGC GTT GAC TGG AGC AAA TAC<br>TTG TCT CCA GAC ACA GTT ACT GGT GGA TTA GGT AGC CAG GTG TTC GGC CCG ATC ATC TCA GCC TCA<br>AAA GCG GTA CCG GTG GTT ATG ACT CGC GGT GCG AGC GAA GTC GGG TTT TAT GTC GCC ACG GGT GGG<br>CAA CAA GCC CTC ATG ATG GGT TTT CTC GTT GTC GAT TCG AGC GAC GCA CCA GCC AGC TAT GGC GAA<br>GCG TAT CAT ACT ATT GGC ACG CGG GAT TCC ATT GCG AAT ACC CCG ATC AAT CAG CCT TAC TTA GGT<br>AGC ACC GCA GCA GAC ATT GAT GCG GAC TCT GAA AGT GAC TGG ACT GCC GAT GAC CGC GAA GAT GTA<br>GCA GAT GAA GGC CCC GCC CAG TTG CTG ACG GCT GAC CAG CTT AGC AAA ACC AAC GAT TTA CTG GAC<br>CTG AAC AAA GCC AAG AAC GGG ACC TAC ACC CTC AAA ATC AAA GCG AAC CCA AAC GGT AAC GCA AAA<br>GCG TAC GTC AAG GCA TGG GTG GAT TTC AAC AAC AAT GGC AAA TTT GAT GAC AAT GAA GGC TCG GTG

	GTG AAG GAG ATT ACC GCC AAC GGG GAT CAT ACG CTG TCC TTT AAC GCC ATT CCT GGC CTT ACC GGC<br>GGC CTG GTG GAC CAG ATT GGC ATG CGG GTA CGC ATT GCG ACG AAT GCA GGG GAT ATT GAG AAA CCG<br>ACA GGT ACC GCG TTC AGT GGG GAA GTA GAG GAT ATG CTG GTT CGC CGT GTC TAT CCG CCA CAA GGC<br>GAA AAG CAG GAA TCT ACT GGC TTC CAA GGA GAA ACC CAG AAT GCT TCG GTG CAC TTT ACC GCA AAA<br>GGA CCG GAT CGC TCC GAT TTT GTA ACC AAC GCG AGC ATG AGC AAT CAA GCG CCA CAG GTT CTG GAT<br>AAT CAG GGC AAC GTT CTG ACG CCG ACC AAT GGT AAT ACC TAT GTA CGT CCC GAG GGA ACG TAC GTG<br>GTG ACA GCC AAT GGC GAT GAT GTC AAC GTT ACG TTC ACT CCG AAC GAG GAT TTC AGC GGT GTT GCG<br>GAG GGT ATT AAC ATT CGT CGC ACT GAC TCA AAT GGT TCC AGC ACG GGT TGG CAG TCG ACG GAT GCA<br>GCA GAT CCG AAT AAG AAC GAT CGC TTG AAC AAC ATG GAC GGC CGT TTT GTG CCA ACC GTC CGC AAA<br>GTG CCT AAA TAC GAC AGT ACG GGC ATT CAG GGC CAG GAT TCC GGT GGA GGT TCT GGA GGT GGT TCC<br>GGT GGA GGT ATG GTG AGC AAG GGC GAG GAG GAT AAC ATG GCC ATC ATC AAG GAG TTC ATG CGC TTC<br>AAG GTG CAC ATG GAG GGC TCC GTG AAC GGC CAC GAG TTC GAG ATC GAG GGC GAG GGC GAG GGC CGC<br>CCC TAC GAG GGC ACC CAG ACC GCC AAG CTG AAG GTG ACC AAG GGT GGC CCC CTG CCC TTC GCC TGG<br>GAC ATC CTG TCC CCT CAG TTC ATG TAC GGC TCC AAG GCC TAC GTG AAG CAC CCC GCC GAC ATC CCC<br>GAC TAC TTG AAG CTG TCC TTC CCC GAG GGC TTC AAG TGG GAG CGC GTG ATG AAC TTC GAG GAC GGC<br>GGC GTG GTG ACC GTG ACC CAG GAC TCC TCC CTG CAG GAC GGC GAG TTC ATC TAC AAG GTG AAG CTG<br>CGC GGC ACC AAC TTC CCC TCC GAC GGC CCC GTA ATG CAG AAG AAG ACC ATG GGC TGG GAG GCC TCC<br>TCC GAG CGG ATG TAC CCC GAG GAC GGC GCC CTG AAG GGC GAG ATC AAG CAG AGG CTG AAG CTG AAG<br>GAC GGC GGC CAC TAC GAC GCT GAG GTC AAG ACC ACC TAC AAG GCC AAG AAG CCC GTG CAG CTG CCC<br>GGC GCC TAC AAC GTC AAC ATC AAG TTG GAC ATC ACC TCC CAC AAC GAG GAC TAC ACC ATC GTG GAA<br>CAG TAC GAA CGC GCC GAG GGC CGC CAC TCC ACC GGC GGC ATG GAC GAG CTG TAC AAG GGT GGT AGC<br>GGT GGC ACC CGT GGT GTG CCG CAT ATT GTT ATG GTT GAT GCA TAT AAA CGC TAT AAA TAA

Table S2. Primers used to amplify the genes of interest and the pET45b vector by PCR. Cloning strategy includes 20 base pairs (bp) overhangs in each terminus for Gibson assembly. FWD = forward primer, REV = reverse primer.

Plasmid	Primer	Sequence 5'→3'
SC3-scGFP/ pET45b	FWD SC3-scGFP	ATG GCA CAC CAT CAC CAC CAT CAC AGC AGC GGT CTG GAA G
	REV SC3-scGFP	CGA TGA GCG CTA TAA ATA AAG CTT GCG GCC GCA CTC GA
	FWD pET45b	GCG ATG AGC GCT ATA AAT AAA GCT TGC GGC CGC ACT CG
	REV pET45b	CTT TAA GAA GGA GAT ATA CCA TGG CAC ACC ATC ACC ACC A
mCh-ST3/ pET45b	FWD ST3 RGVP	GTG GCA CCC GTG GTG TGC CGC ATA TTG TTA TGG TTG ATG C
	REV ST3 RGVP	CGG CAC ACC ACG GGT GCC ACC GCT ACC
	FWD mCh-ST3	ATG GCA CAC CAT CAC CAC CAT CAC AGC
	REV mCh-ST3	TCG AGT GCG GCC GCA AGC TTT ATT TAT AGC GTT TAT ATG C
	FWD pET45b	CGC TAT AAA TAA AGC TTG CGG CCG CAC TCG AGT CTG G
	REV pET45b	TGG TGG TGA TGG TGT GCC ATG GTA TAT CTC CTT CTT AAA G
CshA-mCh-ST3/ pET45b	FWD CshA	GAG AAT CTT TAT TTT CAG GGC ATG GAT GAA ACA AGT GCG AGT GGG GTA C
	REV CshA	GAA CCA CCT CCA GAA CCT CCA CCG GAA TCC TGG CCC TGA ATG C
	FWD mCh-ST3-pET45b	GGA GGT TCT GGA GGT GGT TCC GGT GGA GGT ATG GTG AGC AAG GGC GAG G
	REV mCh-ST3-pET45b	GCC CTG AAA ATA AAG ATT CTC CGG ACC CTG AAA CAG AAC TTC CAG ACC

Table S3. Hydrodynamic diameter of synthetised proteins calculated from dynamic light scattering (DLS) data. All DLS measurements were performed at 10μM protein concentration in triplicate after equilibration at 25°C for 120 seconds. Graphical representation of this data is available in Figure S3. Hydrodynamic diameter distributions were averaged and fitted to a Gaussian. Diameter provided below is the mean of the fitted distribution and the error provided is the standard deviation of the fit.

Construct	D_H (nm) Intensity	D_H (nm) Numbers	PDI[a]
SC3-scGFP	9.2 ± 0.4	4.6 ± 0.2	0.792
[SC3-scGFP][S]	10.6 ± 0.4	5.3 ± 0.2	0.435
mCh-ST3	2.8 ± 0.8	2.6 ± 0.3	0.516
CshA-mCh-ST3	14.5 ± 0.4	8.2 ± 0.2	0.549

[a] Polydispersity Index.

Table S4. Structural parameters of protein constructs calculated from synchrotron radiation-small angle X-ray scattering (SR-SAXS).

PROTEIN CONSTRUCT	I(0)[a]	R_g (Å)[b]	D_{max} (Å)[c]	V (Å^3)[d]	P_E[e]	r (Å)[f]	χ^2
**SC3-scGFP**	(731 ± 3)·10^{-5}	27.4 ± 0.6	133.5	117’439	2.7	43.6	1.442
**[SC3-scGFP][S]**	(293 ± 1)·10^{-4}	30.1 ± 0.6	139	151’128	3.1	44.3	1.396
**mCh-ST3**	(320 ± 3)·10^{-5}	21.48 ± 0.09	95.5	43’802	4.0	28.8	1.110
**CshA-mCh-ST3**	(182 ± 1)·10^{-4}	58 ± 3	239.5	447’821	2.1	76.9	1.255
**mCh-ST3: [SC3-scGFP][S]**	(79 ± 4)·10^{-4}	32 ± 1	138	236’036	2.4	48.4	1.245
**CshA-mCh-ST3: [SC3-scGFP][S]**	(133 ± 1)·10^{-4}	51 ± 4	279	524’767	2.1	82.7	1.292

[a] Scattering intensity at q=0. [b] Radius of gyration. [c] Maximum dimension. [d] Volume. [e] Porod exponent. [f] Radial average. [g] Chi-squared of the fit.

Table S5. Data collected the ZetaView® nanoparticle tracking analysis for 4-LNPs and unmodified LNPs. Particle size, zeta potential (ζ), and particle concentration in scatter mode and fluorescence mode using a 488 nm laser were determined. Modification percentage was calculated based on the number of particles observed in fluorescence mode vs scatter mode. The average values are reported along with the standard deviation (size) or relative standard deviation (concentration).

Construct	Size (nm)	ζ (mV)	Concentration (per mL)	Concentration at 488 nm (per mL)	Modification (%)
**LNPs**	131.5 ± 1.5	-35.8 ± 0.7	(0.900 ± 0.001)·10^{12}	-	-
**4-LNPs**	129.5 ± 1.4	3.7 ± 0.9	(1.13 ± 0.05)·10^{12}	(0.8 ± 0.03)·10^{12}	70

Table S6. Hydrodynamic diameters of LNPs exposed to different conditions calculated from dynamic light scattering (DLS) data. All DLS measurements were performed at in triplicate after equilibration at 25°C for 120 seconds. Graphical representation of this data is available in Figure S15. Hydrodynamic diameter distributions were averaged and fitted to a Gaussian. Diameter provided below is the mean of the fitted distribution and the error provided is the standard deviation of the fit.

Condition	D_H (nm) Intensity	D_H (nm) Numbers	PDI[a]
7-day incubation at 4 °C	140.80 ± 2.95	99.72 ± 2.18	0.061
0.22 µm filtration	134.47 ± 0.75	96.16 ± 3.67	0.059
Overnight dialysis	130.60 ± 1.45	61.32 ± 0.86	0.064
4,000 xg centrifugation	133.57 ± 2.16	95.06 ± 0.92	0.090
Dilution in DMEM	130.63 ± 2.62	68.30 ± 1.82	0.230
Overnight incubation at 37 ºC	150.67 ± 8.12	83.73 ± 4.75	0.023
UV sterilisation	129.33 ± 4.11	80.53 ± 4.98	0.043

[a] Polydispersity Index.

Table S7. Hydrodynamic diameter of LNPs before and after modification with AMBPs calculated from dynamic light scattering (DLS) data. All DLS measurements were performed at in triplicate after equilibration at 25°C for 120 seconds. Graphical representation of this data is available in Figure 3a. Hydrodynamic diameter distributions were

averaged and fitted to a Gaussian. Diameter provided below is the mean of the fitted distribution and the error provided is the standard deviation of the fit.

Sample	D_H (nm) Intensity	D_H (nm) Numbers	PDI[a]
LNPs	143.3 ± 40.8	93.2 ± 20.9	0.112
**1**-LNP	253.4 ± 82.0	163.8 ± 47.3	0.117
**1:2**-LNP	221.4 ± 71.7	131.4 ± 36.3	0.121

[a] Polydispersity Index.

Table S8. CryoEM LNP and membrane size data. All LNP measurements were performed in triplicate and the mean and standard deviation (SD) for each n (n = number of LNPs measured) was calculated. All the values for each sample were plotted (Figure SI17) with the mean and SD of the population. The minimum (min.) and maximum (max.) value measured for each condition can be found below. Last column corresponds to the hydrodynamic dimeter of the samples calculated from DLS data.

Sample	LNP Size (nm)			Membrane Size (nm)			n	D_H (nm) Intensity (100% v/v)
	Mean ± SD	Min.	Max.	Mean ± SD	Min.	Max.		
LNPs	82.3 ± 20.9	32.7	121.8	5.4 ± 0.9	4.0	7.4	24	122.1 ± 19.6
**1**-LNP	88.8 ± 29.4	40.7	202.7	7.1 ± 2.5	4.0	23.3	75	749.7 ± 22.6
**1:2**-LNP	85.9 ± 28.7	32.7	163.6	10.1 ± 5.6	3.9	41.6	72	235.4 ± 18.8
**1:3**-LNP	79.1 ± 20.1	33.7	122.0	10.4 ± 2.8	5.3	16.7	48	170.6 ± 11.8

Table S9. Hydrodynamic diameter of concentrated LNPs before and after modification with protein fusion 1 calculated from dynamic light scattering (DLS) data. All DLS measurements were performed in triplicate after equilibration at 25°C for 120 seconds. Graphical representation of this data is available in Figure 3a. Hydrodynamic diameter distributions were averaged and fitted to a Gaussian. Diameter provided below is the mean of the fitted distribution and the error provided is the standard deviation of the fit.

LNPs (% v/v)	D_H (nm) Intensity	PDI[a]	D_H (nm) Intensity + [SC3-scGFP][S]	PDI[a]
10	130.4 ± 20.4	0.099	164.1 ± 26.8	0.323
100	121.6 ± 30.1	0.077	265.0 ± 21.5	0.211

[a] Polydispersity Index.

List of Equations

Equation S1. Number of lipid molecules in an unilamellar LNP. The radius of the LNP, r, was assumed to be 50 nm, the bilayer thickness, h, was estimated to be 5 nm, and the surface of the lipid headgroup, a, is 0.71 nm^2 for a phosphatidylcholine.

$$N_{lipid} = \frac{[4\pi\,(r)^2 + 4\pi\,(r-h)^2]}{a} \qquad \textbf{(1)}$$

Equation S2. Number of LNPs per millilitre. The molarity of lipids in 1 mL of sample, n_{lipid}, was 0.4 μmol, and the number of lipid molecules in a LNP, N_{lipid}, was calculated to be 80085. N_A is the Avogadro's number.

$$N_{LNPs} = \frac{N_A \cdot n_{lipid}}{N_{lipid}} \qquad \textbf{(2)}$$

Equation S3. Area of AMBP in protein fusion 1 in perpendicular. Superchaged GFP (scGFP) radius, r, is known to be 1.2 nm.

$$A_{p1} = \pi r^2 \qquad \textbf{(3)}$$

Equation S4. Area of AMBP of protein fusion 1 in parallel. scGFP width, w, is known to be 2.4 nm, and the length, l, of protein fusion **1** was assumed to be 13.9nm, value obtained from the D_{max} calculated from SR-SAXS data from Table S4.

$$A_{p2} = w \cdot l \qquad \textbf{(4)}$$

Equation S5. Number of AMBP per LNP. The surface area of the LNP, A_{LNP}, was calculated to be 31416 nm^2, and the area of the AMBP, A_p, was calculated to be either 4.5 or 33.4 nm depending on the AMBP orientation.

$$N_{AMBP} = \frac{A_{LNP}}{A_p} \qquad \textbf{(5)}$$

Equation S6. Number of moles of protein required for monolayer coverage of LNPs. The number of AMBPs per LNP, N_{AMBP}, was found to be either 6945 or 942 molecules, depending on their orientation, and the number of LNPs in solution, N_{LNPs}, was calculated to be $1.5 \cdot 10^{12}$ LNPs/mL. N_A is the Avogadro's number.

$$n_{AMBP} = \frac{N_{AMBP} \cdot N_{LNPs}}{N_A} \qquad \textbf{(6)}$$

Supplementary Figures

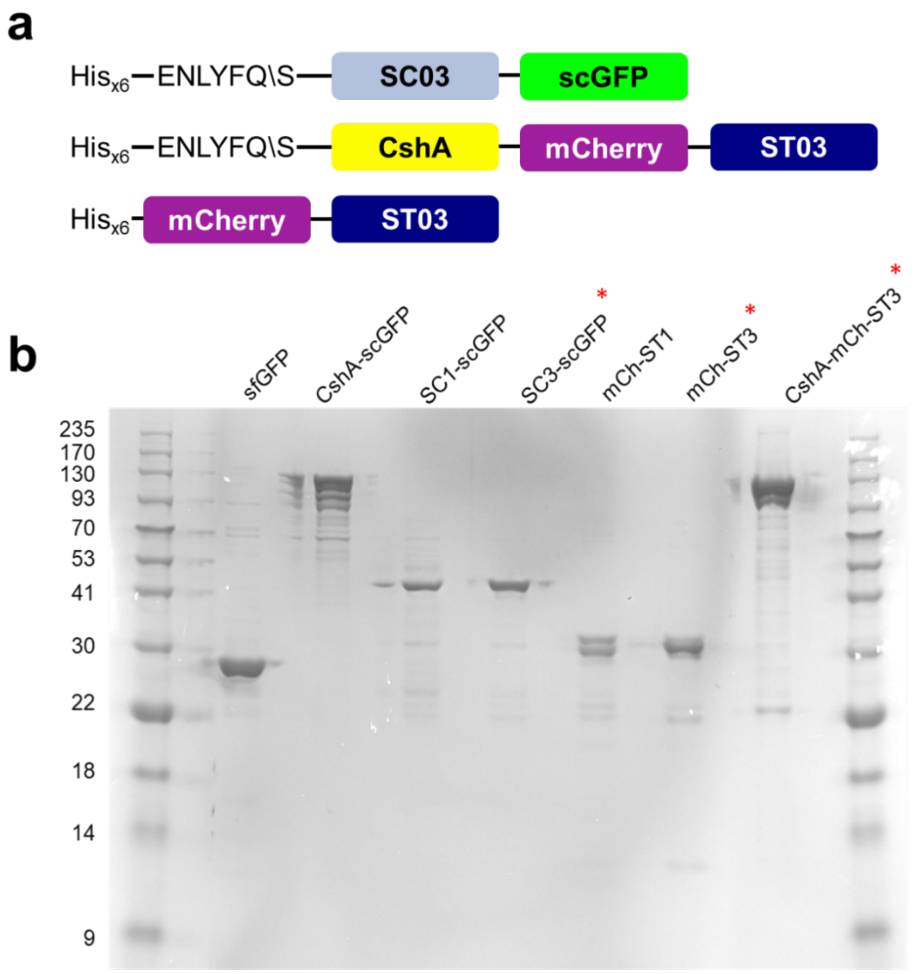

Figure S1. Protein constructs. (a) Schematic of protein constructs. His_{6x} = poly histidine tag, Tobacco Etch Virus (TEV) cleavage site = ENLYFQ, SC03 = SpyCatcher 03, ST03 = SpyTag 03, scGFP = supercharged green fluorescent protein. **(b) SDS-PAGE of purified proteins.** The first and last lanes correspond to BLUeye Prestained Protein Ladder (5 µL). Samples were prepared by mixing 15 µL protein in 5 µL of 4X reducing SDS loading dye. Samples were boiled for 5 minutes at 95ºC before loading 5 µL of each sample in the gel with a spacer lane in between ('empty' spacer lanes that contain protein is due to leak in the loading). Proteins of interest are marked with a red asterisk. Expected masses of the proteins are 45 kDa for **1** (SC3-scGFP), 30 kDa for **3** (mCh-ST3) and 116 kDa for **2** (CshA-mCh-ST3). The other proteins were produced to be used as controls in different experiments.

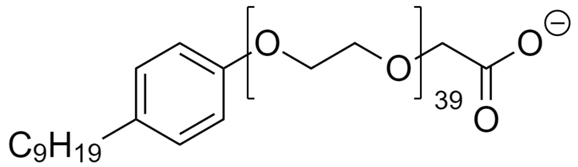

Figure S2. Chemical structure of oxidised IGEPAL CO-890.

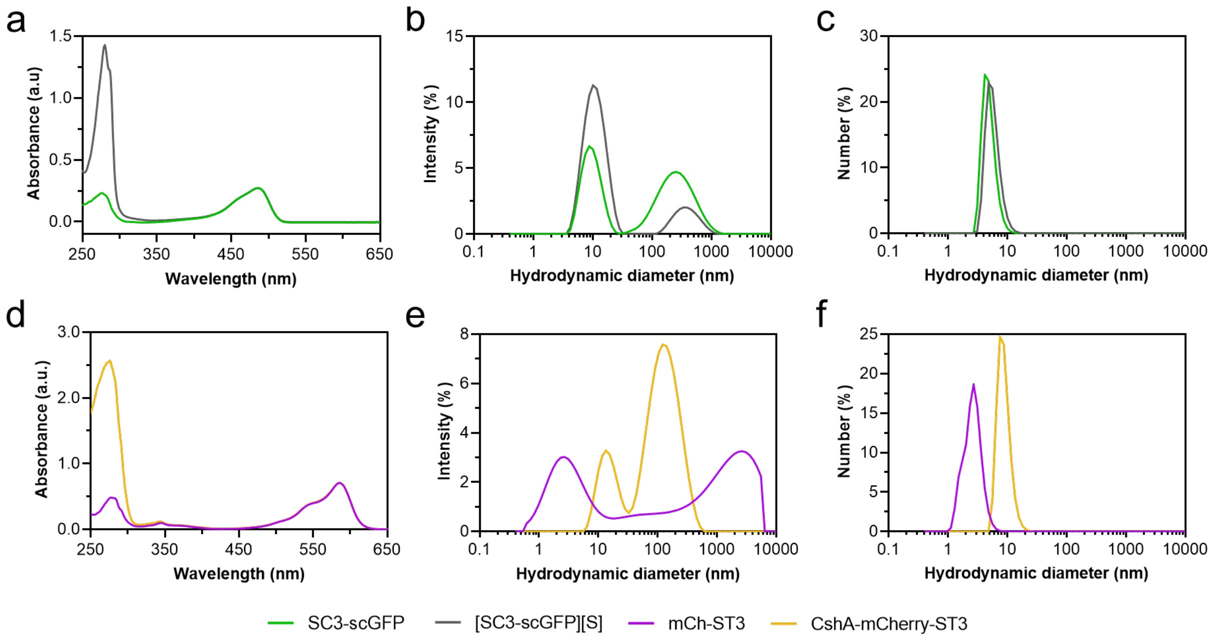

Figure S3. UV-Vis spectroscopy and DLS characterisation of synthetised proteins: **1** (10 µM SC3-scGFP, shown in green, a-c), surfactant-conjugated **1** (10 µM [SC3-scGFP][S], shown in black, a-c) **3** (10 µM mCh-ST3, shown in purple, d-f) and **2** (10 µM CshA-mCh-ST3, shown in yellow, g-i). **(a)** UV-Vis spectroscopy of **1** and the surfactant-conjugated construct **1**. **(b)** Dynamic light scattering (DLS) showing the hydrodynamic diameter intensity and **(c)** number distribution for both constructs showing an increase in size when conjugated to the surfactant as expected. All DLS measurements were performed in triplicate after equilibration at 25°C for 120 seconds. Average of the three measurements is plotted for each sample. **(d)** UV-Vis spectroscopy of **3** (shown in purple) and **2** (shown in yellow). **(e)** DLS showing the hydrodynamic diameter intensity and **(f)** number distribution of **3** and **2**.

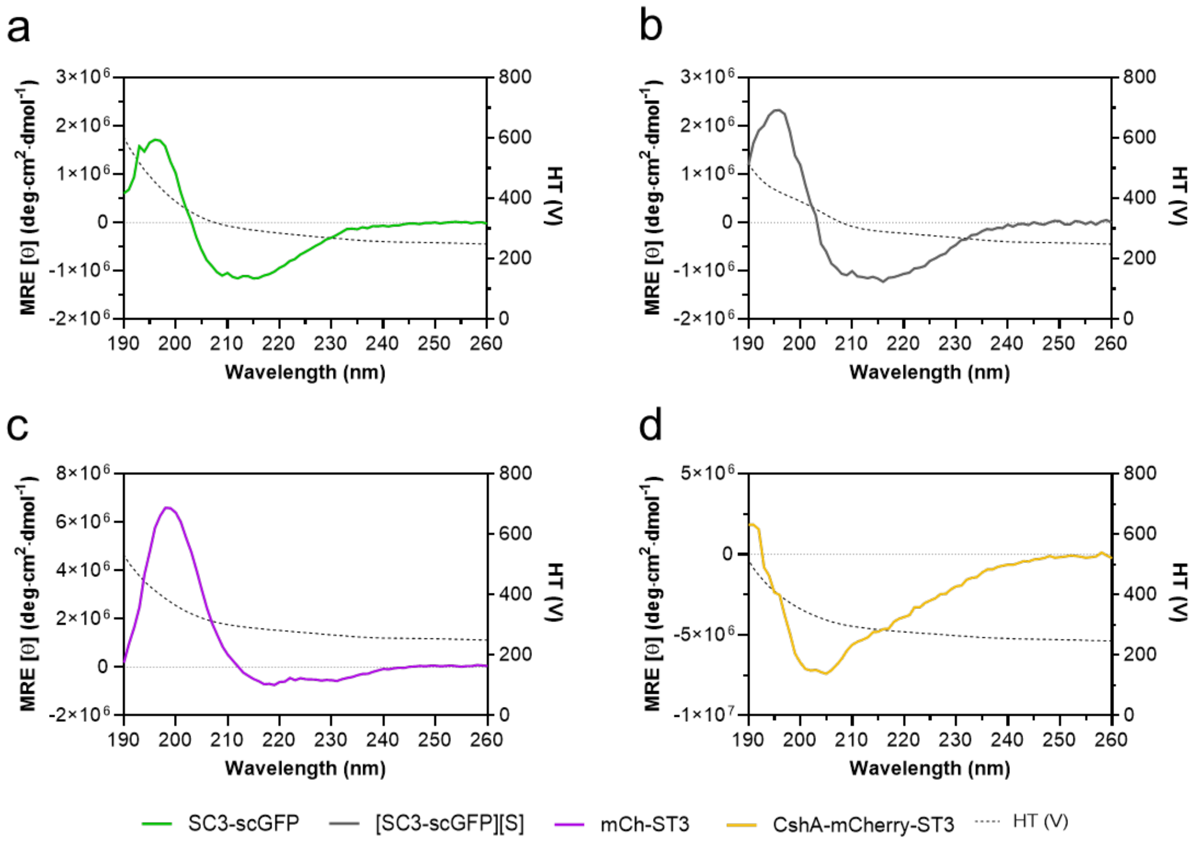

Figure S4. Circular dichroism (CD) spectroscopy of synthetised proteins: (a) 1 (SC3-scGFP, shown in green), **(b)** surfactant-conjugated **1** ([SC3-scGFP][S], shown in black), **(c) 3** (mCh-ST3, shown in purple) and **(d) 2** (CshA-mCh-ST3, shown in yellow). The associated high-tension (HT) voltage is plotted on the right-hand Y-axis (shown in black dashed line). All samples were measured at 25 ºC at a concentration of 0.1 mg·mL^{-1} (10 mM potassium phosphate buffer with 50 mM sodium sulphate at pH 8.0) in triplicate and the average plotted.

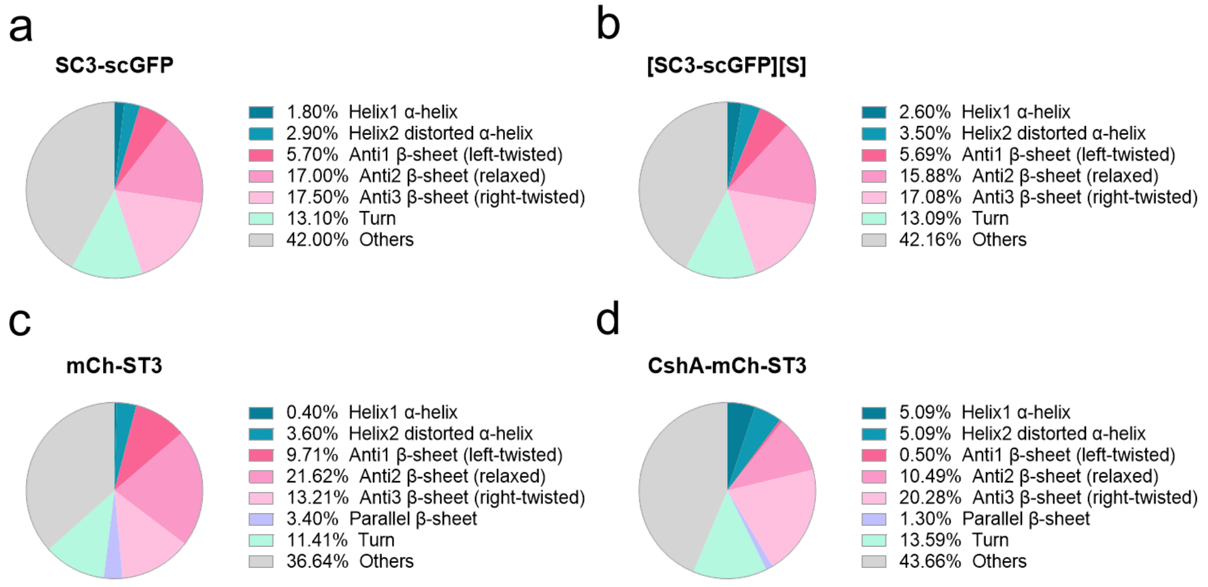

Figure S5. Secondary structure of synthetised proteins calculated from CD spectroscopy data: (a) 1 (SC3-scGFP), **(b)** surfactant-conjugated **1** ([SC3-scGFP][S]), **(c) 3** (mCh-ST3) and **(d) 2** (CshA-mCh-ST3). CD data was deconvoluted using BeStSel software. 'Others' include loops, bends, unstable left-handed π-helix and β-bridges.

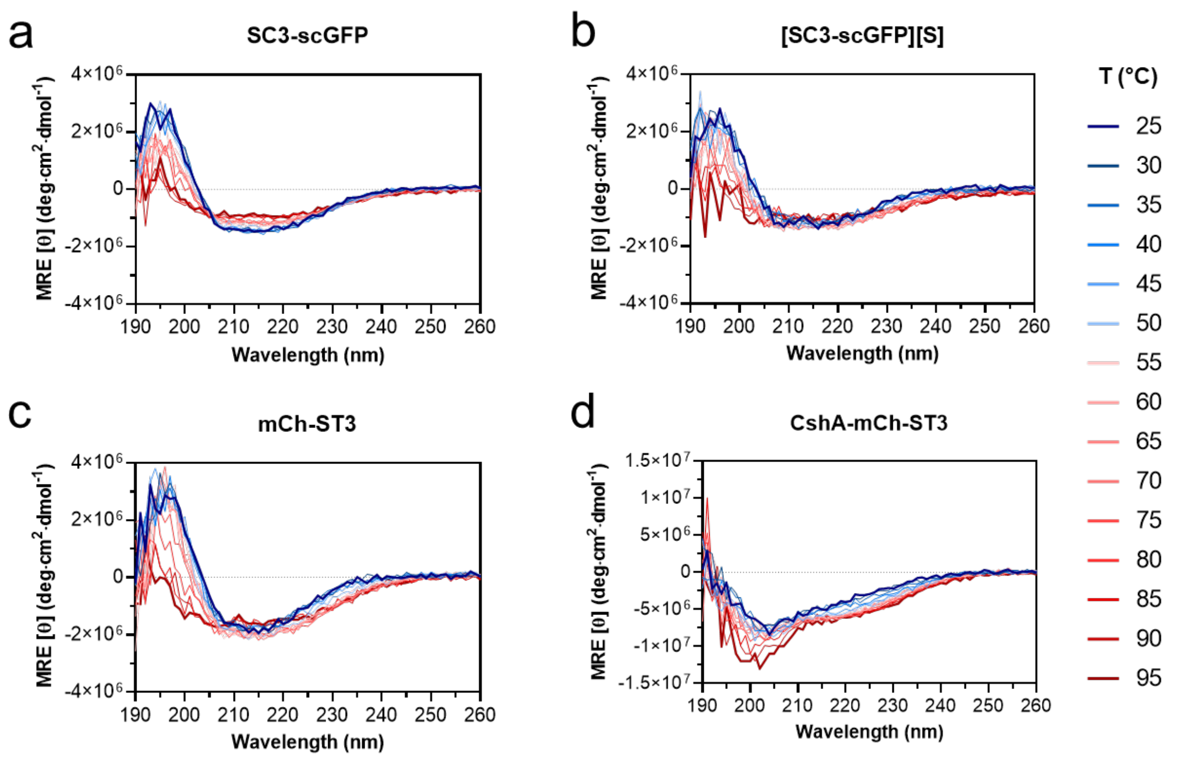

Figure S6. CD spectroscopy data of temperature ramp of synthetised proteins. CD spectra of **(a) 1** (SC3-scGFP), **(b)** surfactant-conjugated **1** ([SC3-scGFP][S]), **(c) 3** (mCh-ST3) and **(d) 2** (CshA-mCh-ST3) (0.1 mg·mL^{-1}, 10 mM potassium phosphate buffer with 50 mM sodium sulphate at pH 8.0) were measured from 25 to 95 °C (blue to red traces).

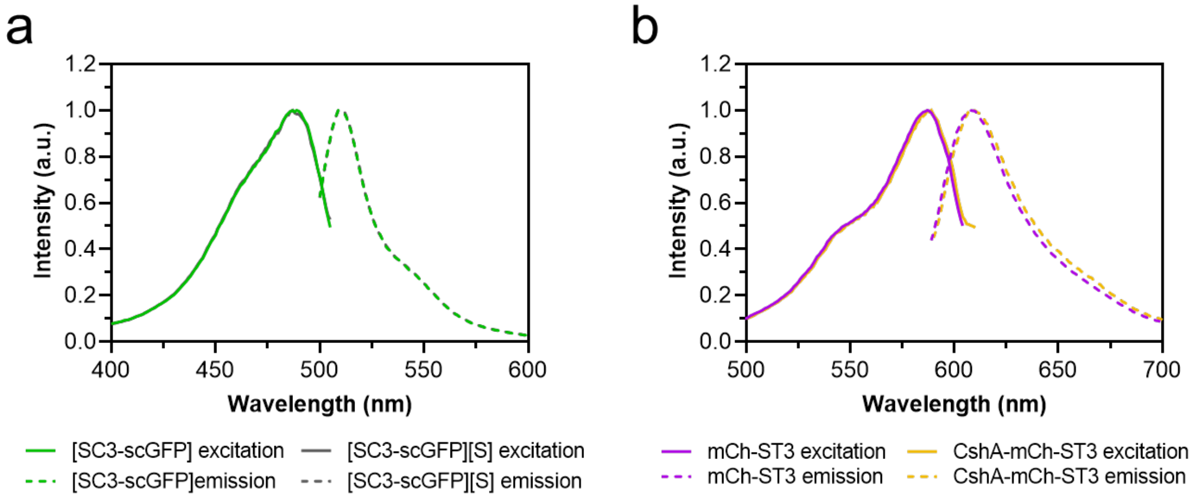

Figure S7. Fluorescence spectroscopy of synthetised proteins. (a) Excitation spectra of **1** (SC3-scGFP, shown in green) and surfactant-conjugated **1** ([SC3-scGFP][S], shown in black) was measured at 510 nm emission and emission spectra of **1** (shown in green, dashed line) and surfactant-conjugated **1** (shown in black, dashed line) was measured at 488 nm excitation. Surfactant conjugation of SC3-scGFP showed no effect on the fluorescence profile of the protein. Spectra was fitted to a Lorentzian (Cauchy) distribution to determine the λ_{Ex} to be 483 nm and the λ_{Em} to be 510 nm. Measurements performed in 20 mM Tris-HCl pH 7.5, 0.5 M NaCl buffer. **(b)** Excitation spectra of **3** (mCh-ST3, shown in purple) and **2** (CshA-mCh-ST3, shown in yellow) was measured at 610 nm emission and emission spectra of **3** (shown in purple, dashed line) and **2** (shown in yellow, dashed line) was measured at 585 nm excitation. Spectra was fitted to a Gaussian distribution to determine the λ_{Ex} to be 586 and 583 nm respectively, and the λ_{Em} to be 610 and 611 nm respectively. CshA fusion lead to a 2 nm blue shift of mCherry excitation maximum and a 1 nm red shift of the emission maximum. Measurements performed in 20 mM Tris HCl pH 7.5, 300 mM NaCl buffer.

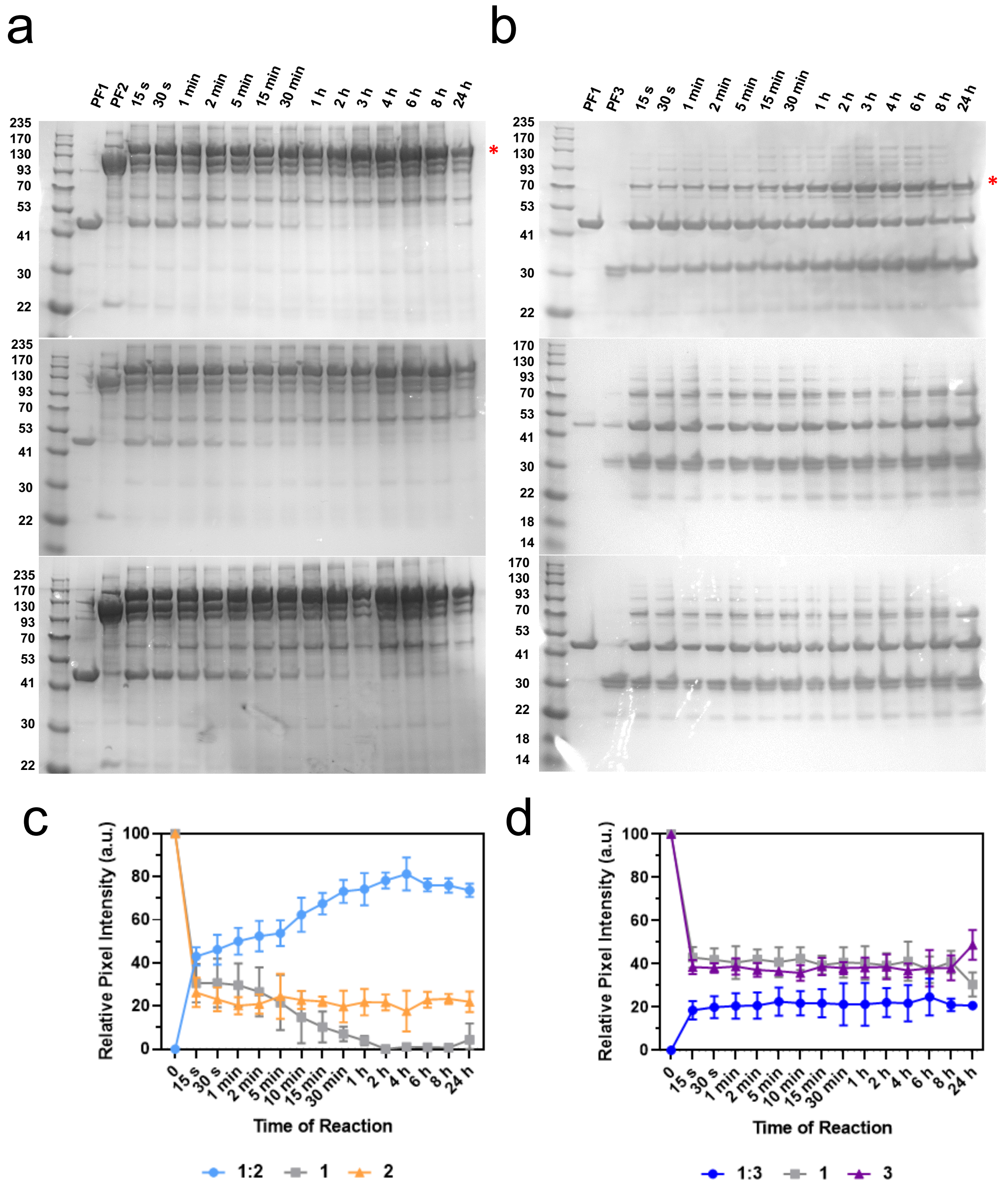

Figure S8. SDS-PAGE of reaction between SpyCatcher and SpyTag fusion constructs. (a) Reaction between **2** (CshA-mCh-ST3, *ca.* 115 kDa) and **1** ([SC3-scGFP][S], *ca.* 43 kDa). **(b)** Reaction between **3** (mCh-ST3, *ca.* 29 kDa) and **1** (SC3-scGFP, *ca.* 43 kDa). Reactions were performed at 37°C in triplicate by mixing both proteins to a final 10 µM concentration (0.5 mL reaction volume, 20 mM Tris-HCl buffer at pH 7.0). Aliquots of the reaction (15 µL) were quenched at the specified time points by mixing with 4X SDS loading dye (5 µL) and boiling for 5 minutes at 95°C. Desired product is signified with red star respectively. **(c)** Relative pixel intensity of **1:2** reaction and **(d) 1:3** reaction over 24 hours calculated from the SDS-PAGE images. Each protein band was normalised from the total pixel intensity of each lane and the mean ± SD was plotted for the reaction substrates and the final products.

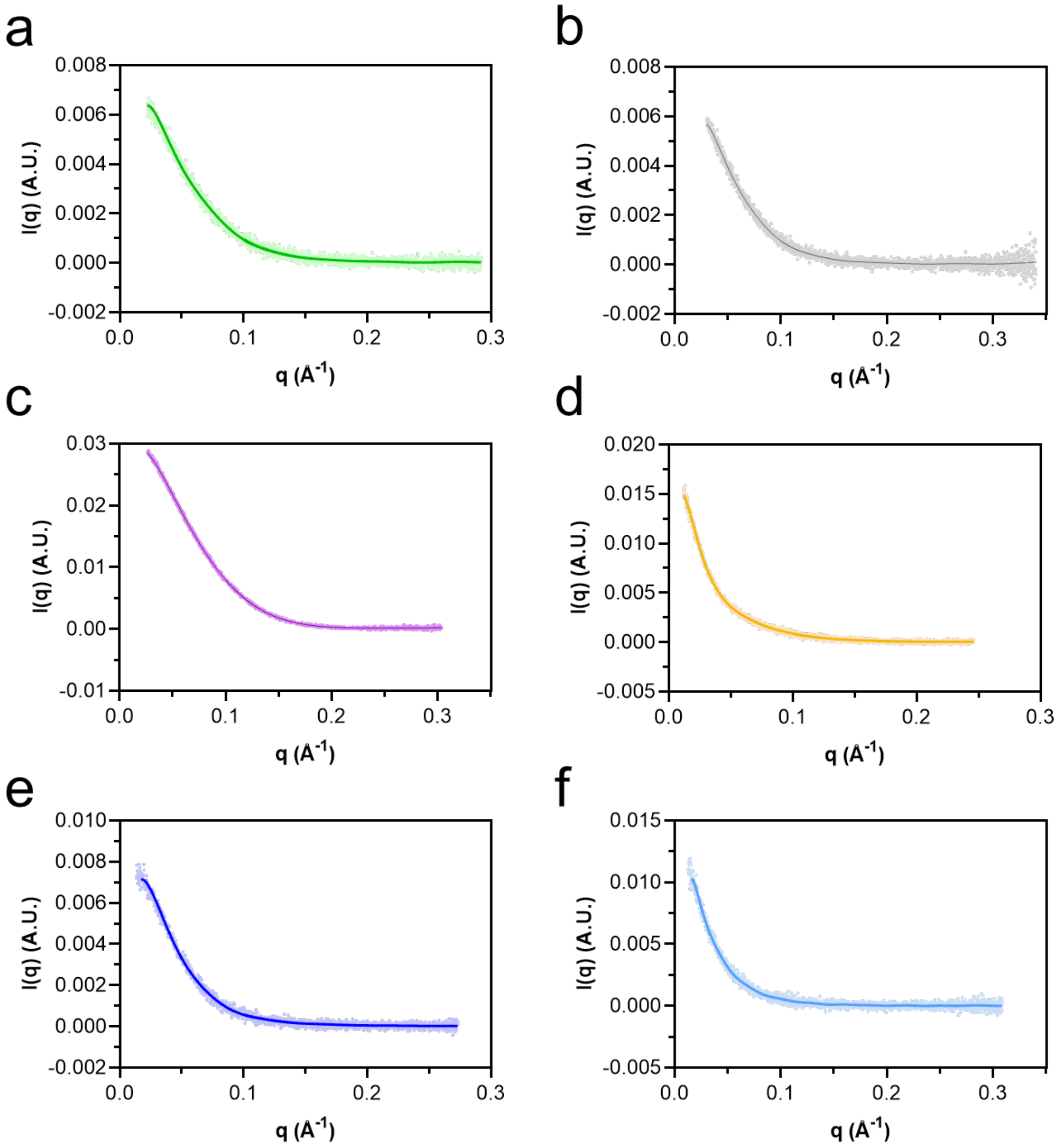

Figure S9. Synchrotron radiation small angle X-ray scattering (SR-SAXS) of protein constructs. Data was collected at 25°C, shown as dots, and processed using ScÅtter, fit is shown as a darker line (χ^2 represents the fit score). **(a) 1** (SC3-scGFP, shown in green, χ^2=1.442), **(b)** surfactant-conjugated **1** ([SC3-scGFP][S], shown in grey, χ^2=1.396), **(c) 3** (mCh-SC3, shown in purple, χ^2=1.110), **(d) 2** (CshA-mCh-SC3, shown in orange, χ^2=1.255), **(e) 1:3** (mCh-ST3:[SC3-scGFP][S], shown in navy blue, χ^2=1.245), and **(f) 1:2** (CshA-mCh-ST3:[SC3-scGFP][S], shown in light blue, χ^2=1.292).

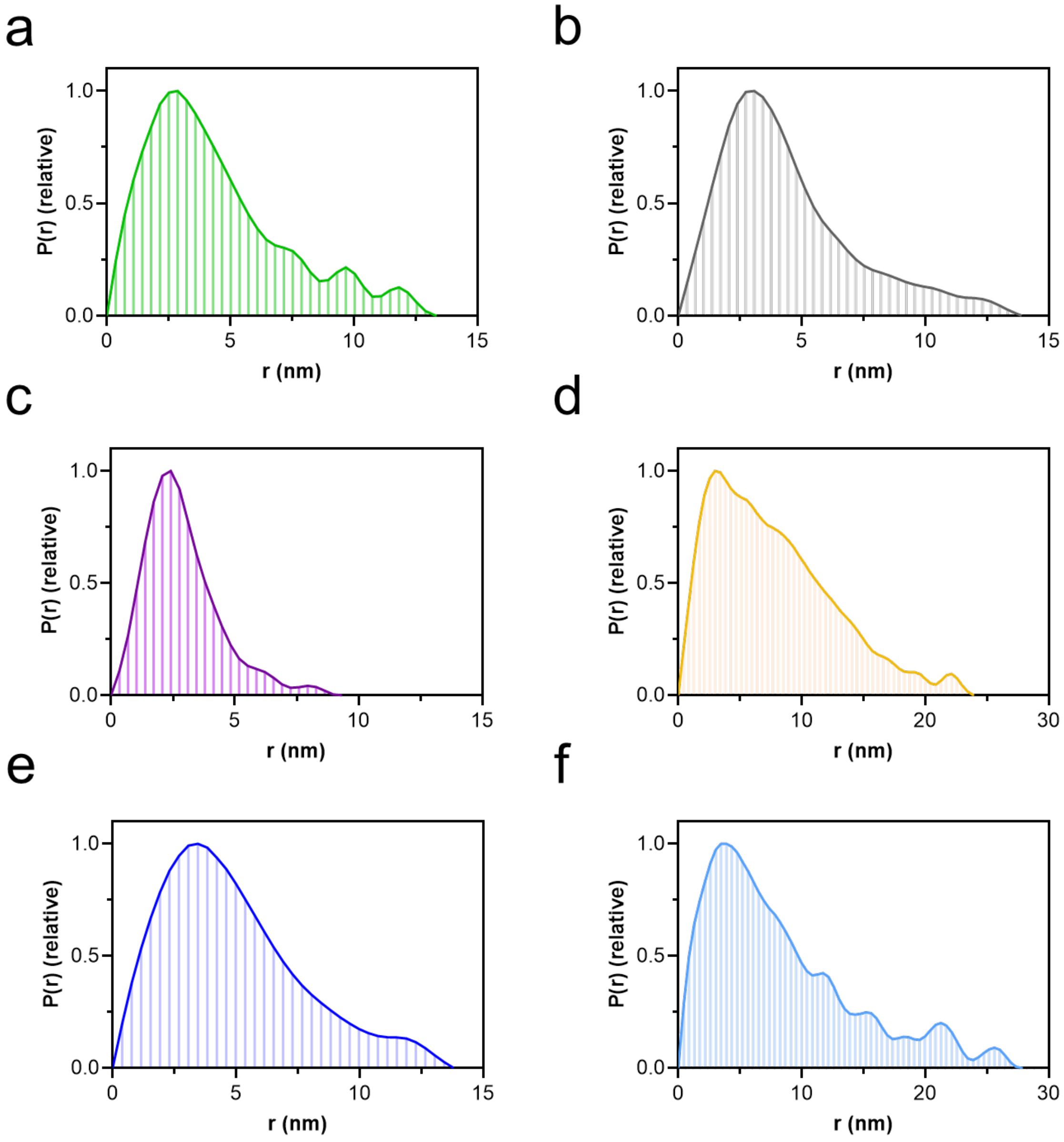

Figure S10. Pair-distance distribution function P(r) for protein constructs calculated using ScÅtter from synchrotron radiation small angle X-ray scattering (SR-SAXS) data. (a) 1 (SC3-scGFP, shown in green, χ^2=1.442), **(b)** surfactant-conjugated **1** ([SC3-scGFP][S], shown in grey, χ^2=1.396), **(c) 3** (mCh-SC3, shown in purple, χ^2=1.110), **(d) 2** (CshA-mCh-SC3, shown in orange, χ^2=1.255), **(e) 1:3** (mCh-ST3:[SC3-scGFP][S], shown in navy blue, χ^2=1.245), and **(f) 1:2** (CshA-mCh-ST3:[SC3-scGFP][S], shown in light blue, χ^2=1.292).

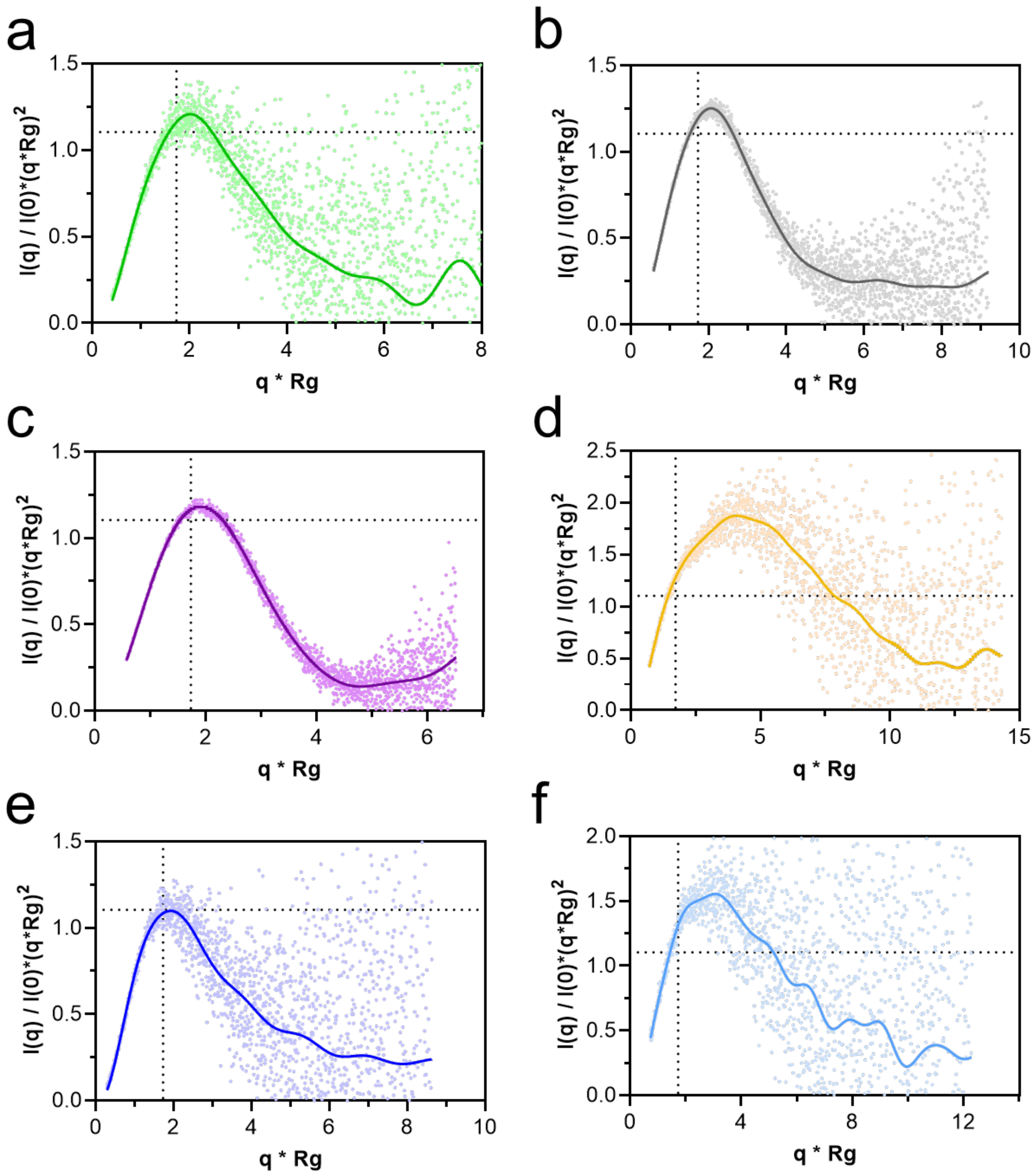

Figure S11. Dimensionless Kratky plot of protein constructs from synchrotron radiation small angle X-ray scattering (SR-SAXS) data. Data was collected at 25°C, shown as dots, and processed using ScÅtter, fit is shown as a darker line (χ^2 represents the fit score). **(a) 1** (SC3-scGFP, shown in green, χ^2=1.442), **(b)** surfactant-conjugated **1** ([SC3-scGFP][S], shown in grey, χ^2=1.396), **(c) 3** (mCh-SC3, shown in purple, χ^2=1.110), **(d) 2** (CshA-mCh-SC3, shown in orange, χ^2=1.255), **(e) 1:3** (mCh-ST3:[SC3-scGFP][S], shown in navy blue, χ^2=1.245), and **(f) 1:2** (CshA-mCh-ST3:[SC3-scGFP][S], shown in light blue, χ^2=1.292).

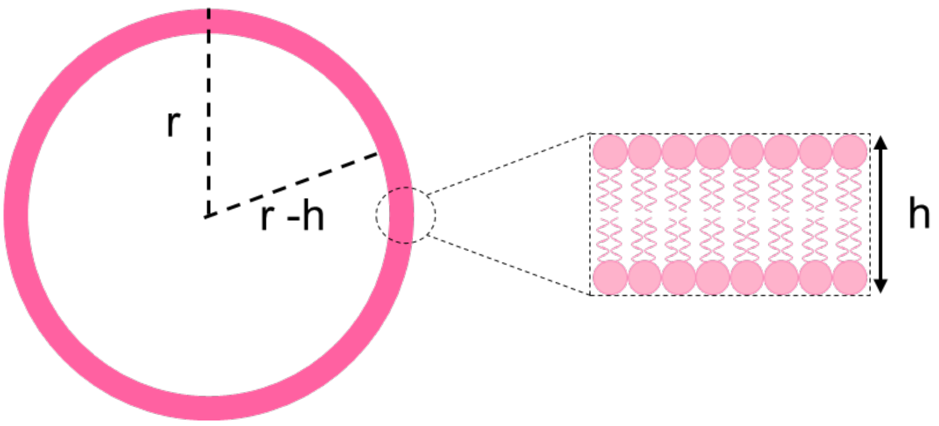

Figure S12. Schematic of a large unilamellar vesicle with a diameter of 100 nm. To calculate the surface area of the vesicles, both the inner and outer layer surface areas are required. The outer layer surface area is calculated from the radius (r), and the inner layer surface area is calculated from (r-h), where h=bilayer thickness, approximately 5 nm for a unilamellar bilayer.

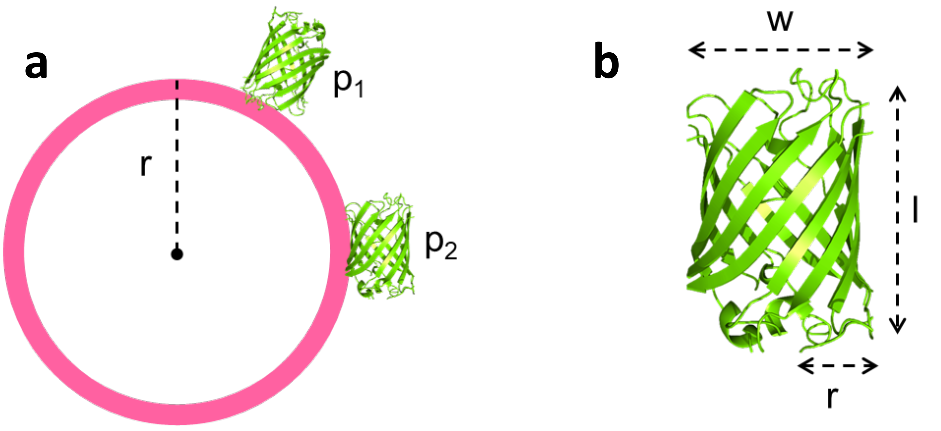

Table S13. Schematic of the possible interactions between AMBPs and LNPs. (a) AMBPs could anchor to the lipid bilayer in different orientations, the two extremes are shown as p_1 and p_2. **(b)** To calculate the concentration of AMBP required for monolayer coverage of the LNP, the area of the AMBP was calculated for p_1 and p_2, using Equation S3 and Equation S4 respectively.

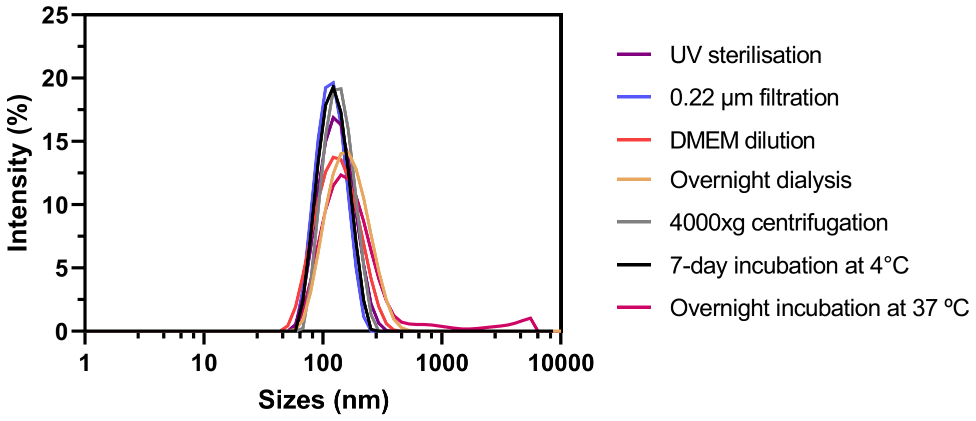

Figure S14. Hydrodynamic diameters of LNPs exposed to different conditions calculated from dynamic light scattering (DLS) data. All DLS measurements were performed at in triplicate after equilibration at 25°C for 120 seconds.

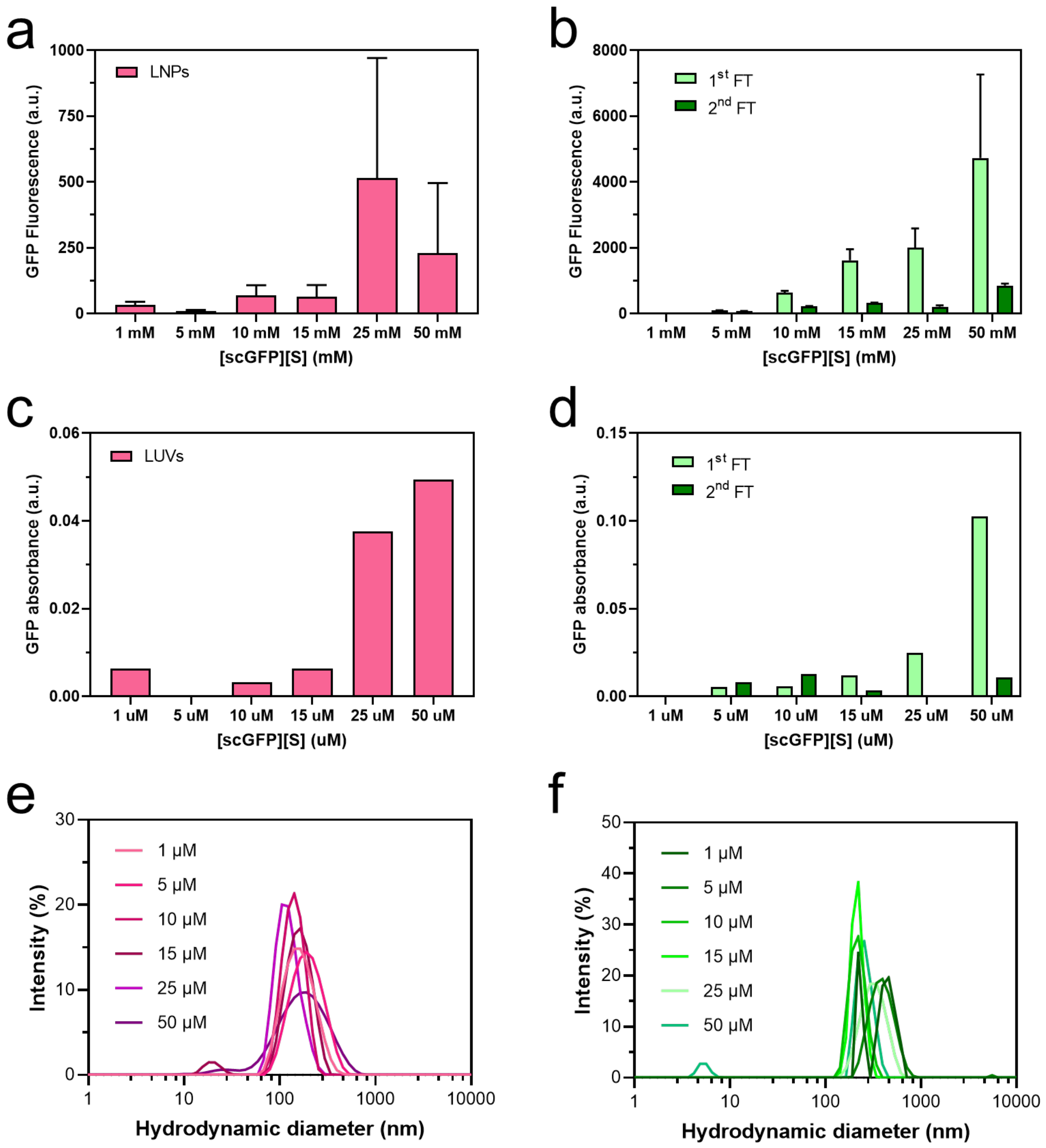

Figure S15. Determination of optimal protein concentration required to modify LNPs using [scGFP][S] anchor domain. (a) GFP fluorescence spectroscopy (λ_{Em} = 515 nm) of **5**, [scGFP][S], modified LNPs after two rounds of vivaspin centrifugation to remove unbound protein. (b) GFP fluorescence spectroscopy (λ_{Em} = 515 nm) of centrifugation flowthroughs to determine unbound protein. (c) GFP UV-Vis spectroscopy (λ_{Abs} = 488 nm) of **5** modified LNPs after two rounds of vivaspin centrifugation to remove unbound protein. (d) GFP UV-Vis spectroscopy (λ_{Abs} = 488 nm) of centrifugation flowthroughs to determine unbound protein. (e) Hydrodynamic diameters of **5** modified LNPs calculated from dynamic light scattering (DLS) data after two rounds of vivaspin centrifugation to remove unbound protein. (f) Hydrodynamic diameters of flowthroughs from the first round of centrifugation calculated from dynamic light scattering (DLS) data showing aggregated protein. All DLS measurements were performed at in triplicate after equilibration at 25°C for 120 seconds.

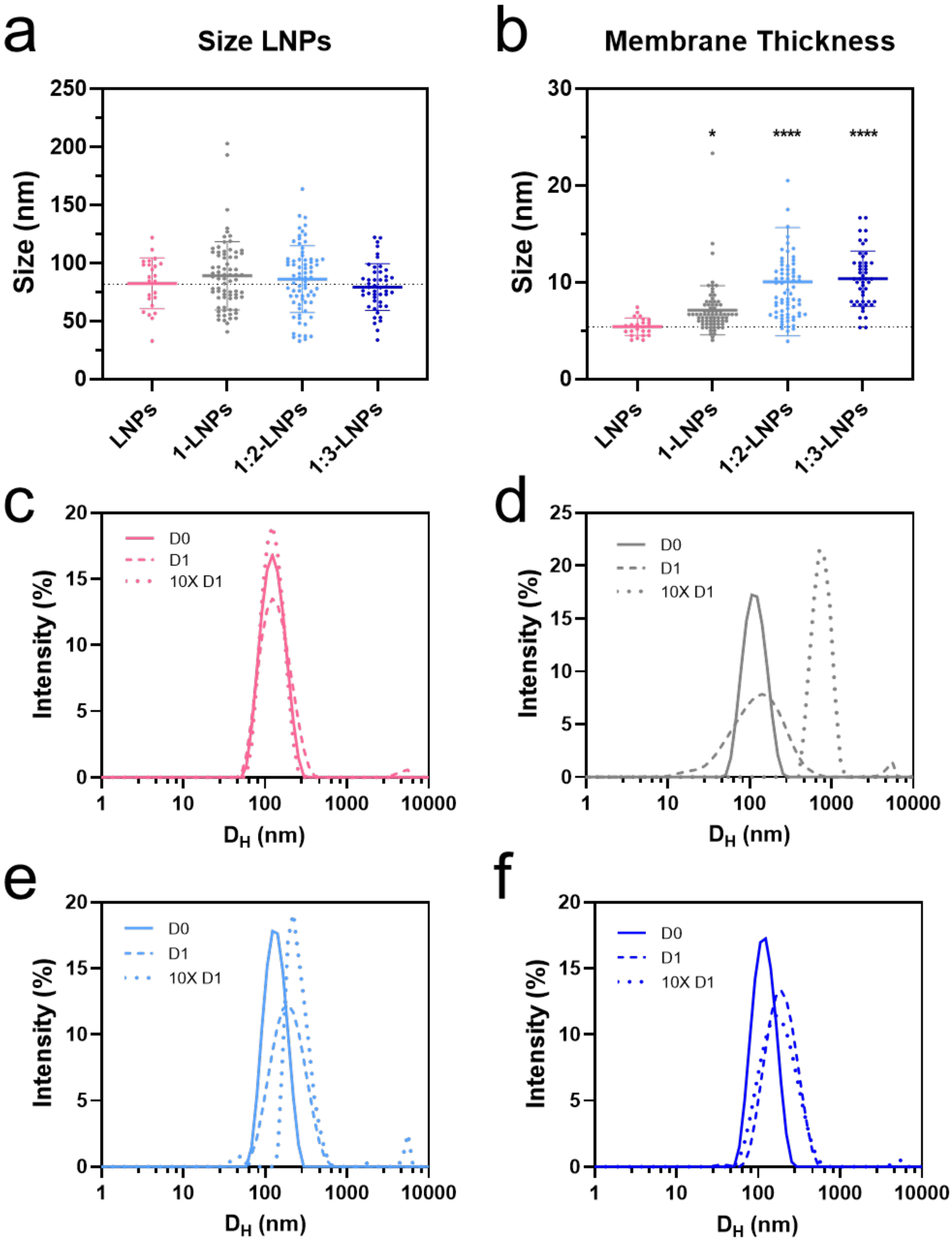

Figure S16. Size (a), and membrane thickness (b) of 1-, 1:2-, and 1:3-modified and native LNPs determined by cryo-EM images. Images of distinct LNPs were manually analysed using the Fiji software[4] measuring tool. Plots show each data point, which is the mean resulting of 3 measurements for each distinct **1**-LNPs (n=47), **1:2**-LNPs (n=73), **1:3**-LNPs (n=74), or native LNPs (n=23), and the mean ± SD of each population. The overall size of the LNPs were found to be not significantly different statistically, whilst the membrane thickness was found to be statistically different in **1:2**- and **1:3**-LNPs in a 2way ANOVA analysis performed in GraphPad Prism (*= p<0.05, **** = p<0.0001). Some LNPs were part of clumps and could not be analysed by this method, so this data is only relevant for isolated LNPs or distinct LNPs in a clump. (c) Hydrodynamic radius of LNPs, (d) 1-LNPs, (e) 1:2-LNPs and (f) 1:3-LNPs calculated from DLS data of samples after extrusion (D0), the next day after AMBP modification (D1) and after concentrating the LNPs from 10% to 100% v/v (10X D1) using viva-spin centrifugation.

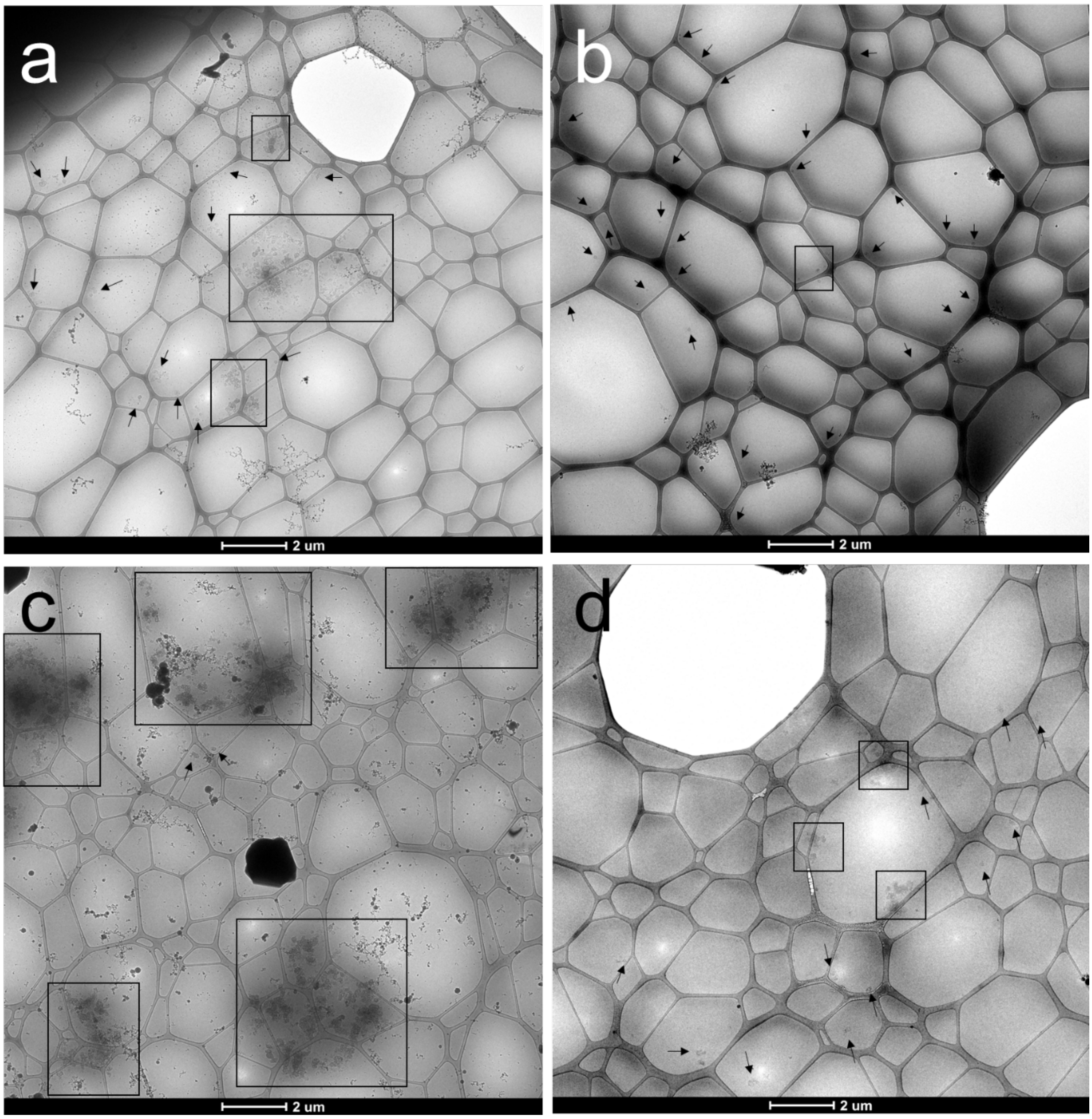

Figure S17. Representative image of a zoom-out grid of (a) 1:2 LNP, (b) native LNP, (c) 1 LNP, and (d) 1:3 LNP cryo-EM samples. Black squares highlight areas with clusters of LNPs, and black arrows are pointing single LNPs and groups of few LNPs. Scale bars = 2 μm.

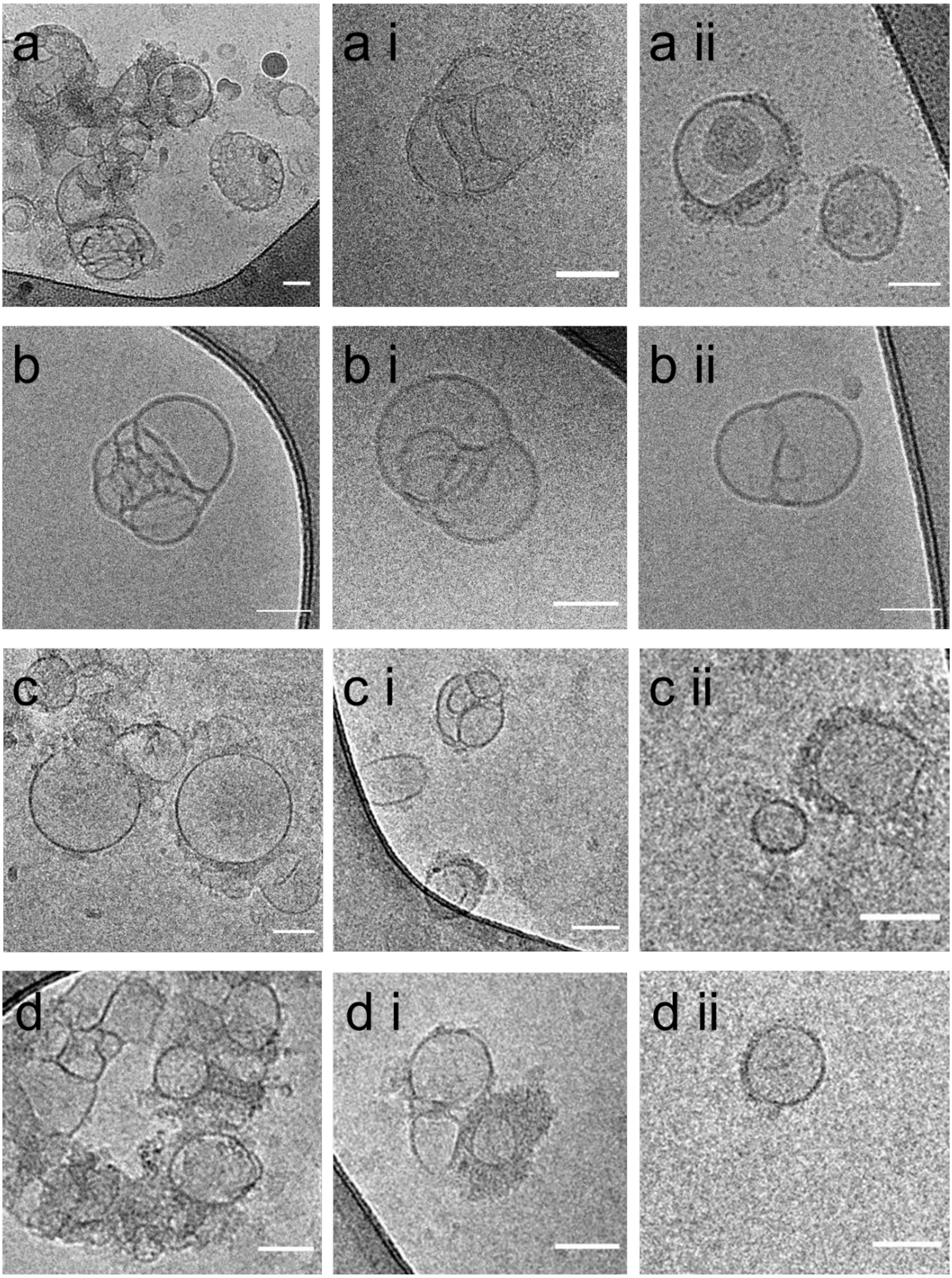

Figure S18. Cryo-TEM images of (a) 1:2-LNPs, (b) un-modified LNPs, (c) 1-LNPs and (d) 1:3-LNPs. Samples contained approximately $1.5 \cdot 10^{13}$ LNPs/mL and show AMBPs give contrast to the membranes, and they tend to clump with one another, especially in the presence of AMBP. Scale bars: 50 nm.

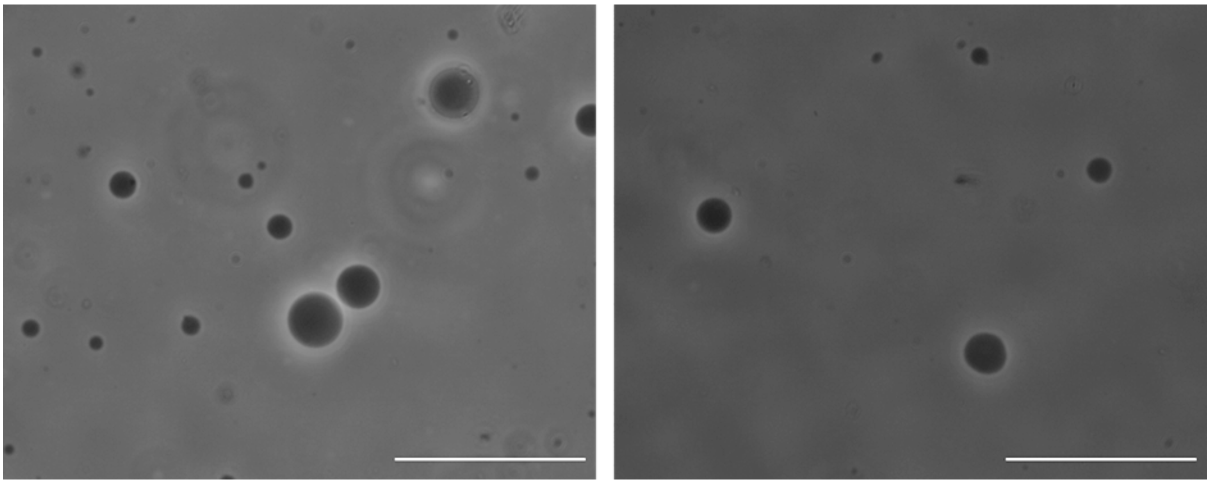

Figure S19. Phase contrast microscopy images of giant unilamellar vesicles (GUVs). GUVs were synthesised via an electroformation method in a composition of CHOL, DOPC, DOPS, and DOPE in a 55:21:16:8 % ratio. Lipid films were hydrated in 0.5 M sucrose and imaged in 0.5 M glucose (both in 20 mM phosphate buffer at pH 7.5). Scale bars = 100 µm.

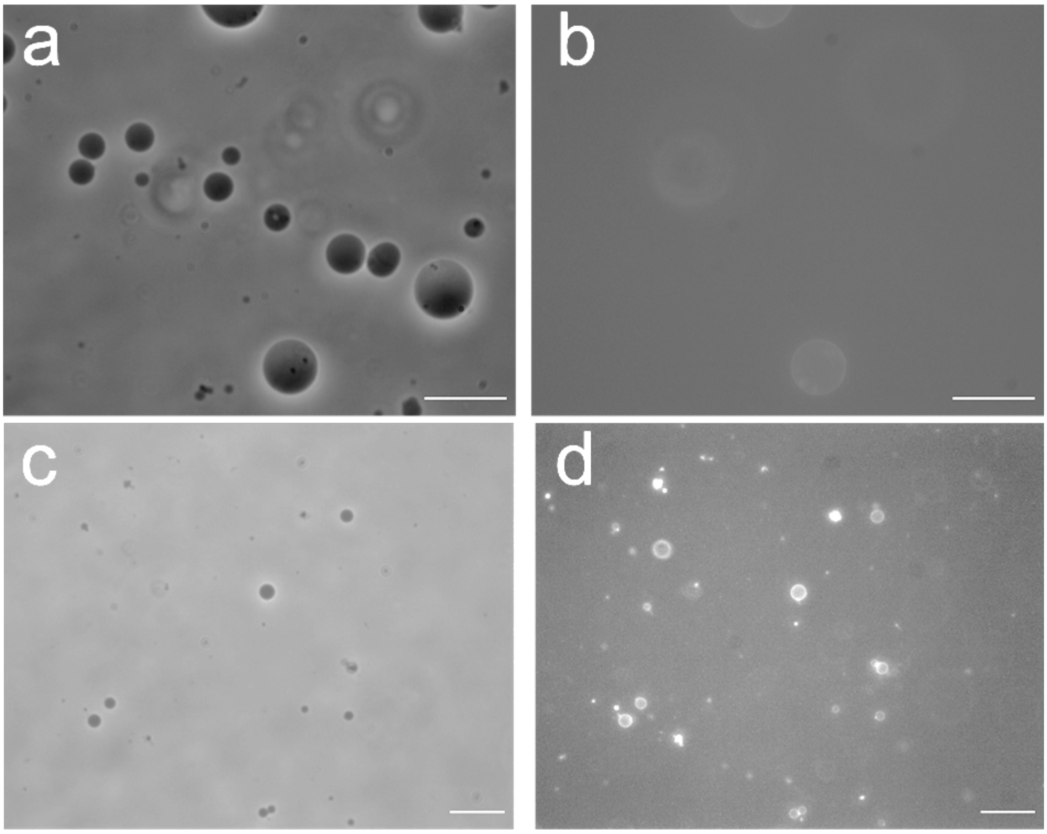

Figure S20. Phase contrast microscopy images of giant unilamellar vesicles (GUVs) after addition of [scGFP][S] AMBP. (a) Phase contrast microscopy images displaying GUVs after addition of 2 µL of 10 µM **5**, [scGFP][S], with diameters between 1 and 40 µm. (b) The green fluorescence channel of image (a) exhibiting one GUV with a fluorescent lipid bilayer. (c) Phase contrast microscopy images displaying GUVs after 24 h from addition of 2 µL of 10 µM **5**, with diameters between 1 and 40 µm. (d) The green fluorescence channel of image (c) exhibiting one GUV with a fluorescent lipid bilayer. GUVs were synthesised via an electroformation method in a composition of CHOL, DOPC, DOPS, and DOPE in a 55:21:16:8 % ratio. Lipid films were hydrated in 0.5 M sucrose and imaged in 0.5 M glucose (both in 20 mM phosphate buffer at pH 7.5). Scale bars = 50 µm.

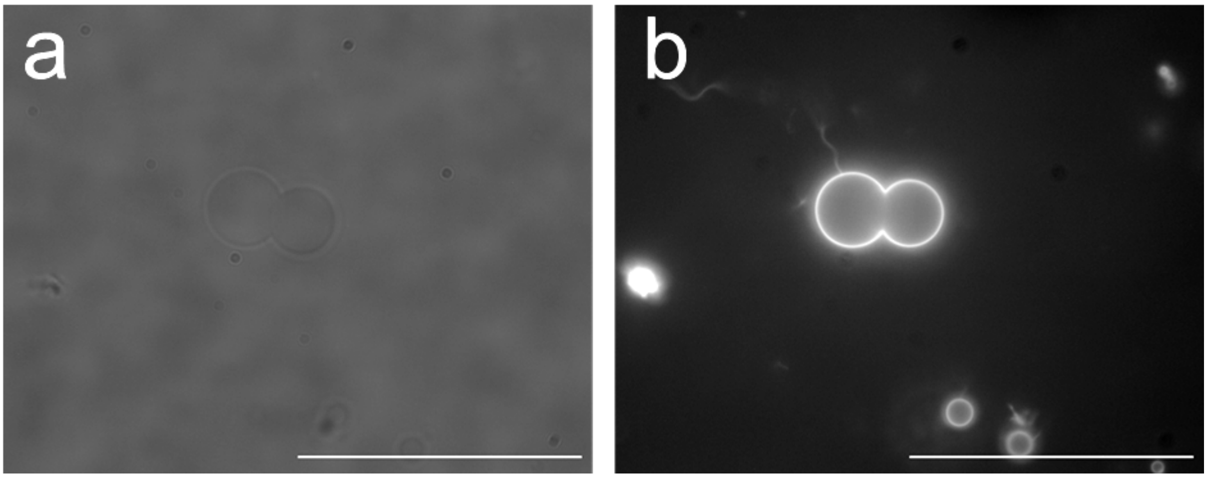

Figure S21. Phase contrast microscopy images of giant unilamellar vesicles (GUVs) after addition of [CshA-scGFP][S] AMBP. (a) Phase contrast microscopy images displaying GUVs modified with **4**, [CshA-scGFP][S]. (b) The green fluorescence channel of image (a) exhibit two GUVs fusing, showing no fluorescence on the inner membranes of fused GUVs. GUVs were synthesised via an electroformation method in a composition of CHOL, DOPC, DOPS, and DOPE in a 55:21:16:8 % ratio. Lipid films were hydrated in 0.5 M sucrose and imaged in 0.5 M glucose (both in 20 mM phosphate buffer at pH 7.5). Scale bars = 50 μm.

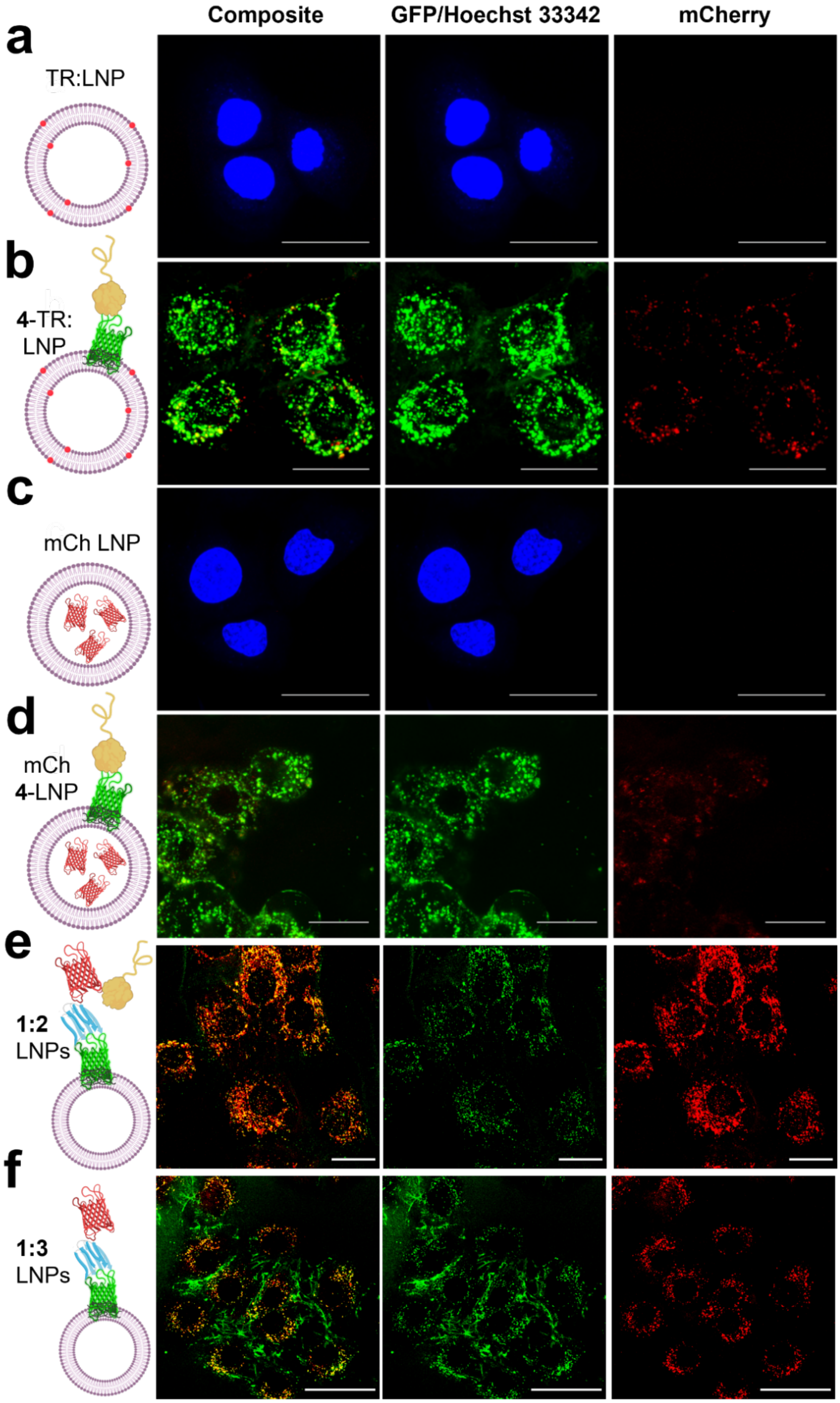

Figure 22. Confocal images of C2C12 cells after exposure to various LNP conditions. C2C12s (250'000 cells/dish) were incubated with (a) TR:LNPs, (b) **4**-TR:LNPs (c) mCherry loaded LNPs, (d) mCherry loaded **4**-LNPs, (e) **1:2**-LNPs, or (f) **1:3**-LNPs (approximately 1.5 x 10^{11} vesicles, 100 µL) for 2 hours at 37 ºC. Cells were washed 3 times with PBS before treatment with Hoechst 33342 dye (10 µg/mL), when specified, and before imaging. GFP fluorescence shown in green, mCherry fluorescence shown in red and Hoechst 33342 shown in blue. Scale bars = 25 µm.

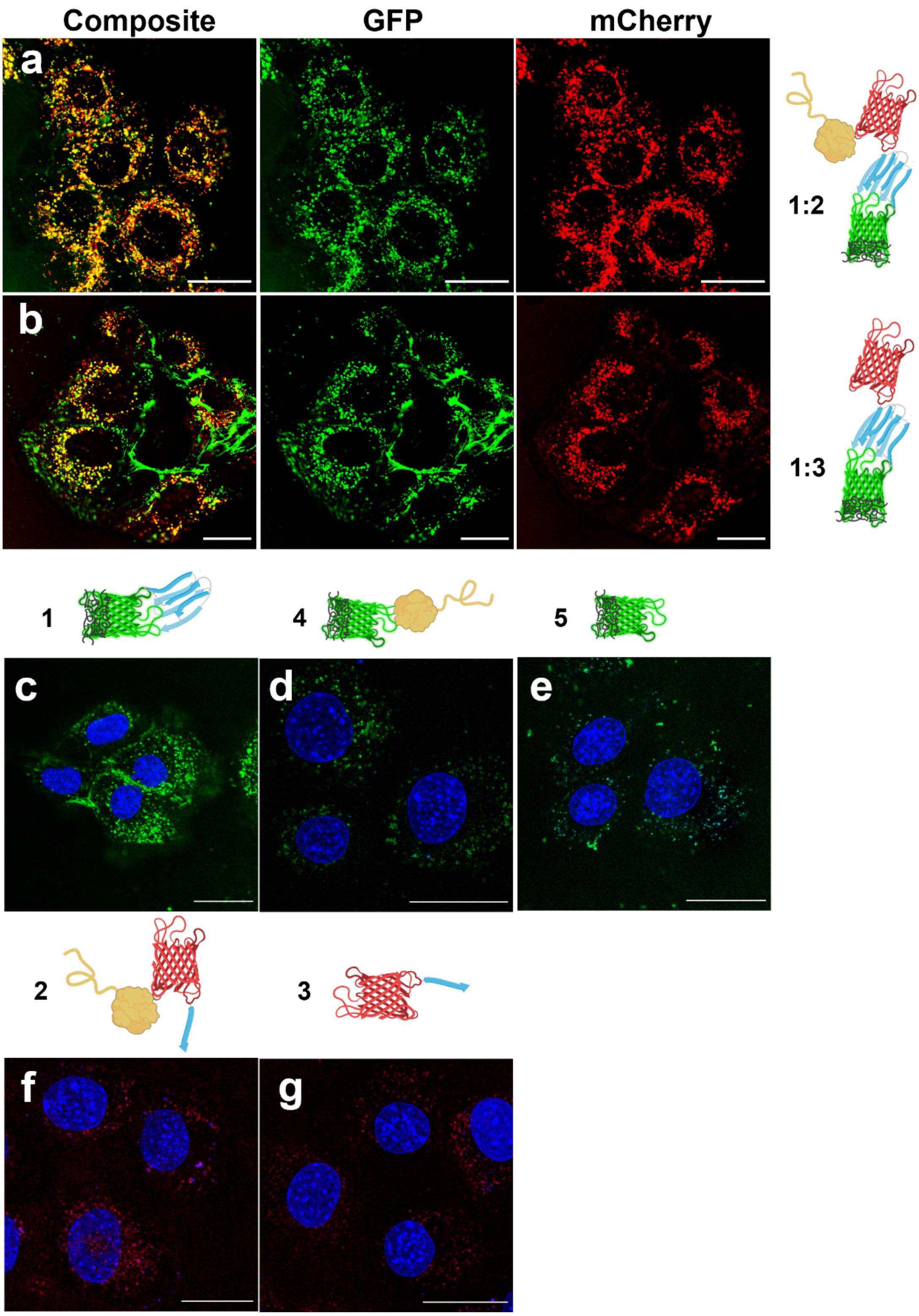

Figure S23. Confocal images of C2C12 cells after exposure to various conditions. C2C12s (250’000 cells/dish) were incubated with (a) **1:2**, (b) **1:3,** (c) **1**, (d) **4**, (e) **5**, (f) **2**, and (g) **3** (1 µM protein, 100 µL) for 2 hours at 37 ºC. GFP fluorescence shown in green and mCherry fluorescence shown in red. Scale bars = 25 µm.

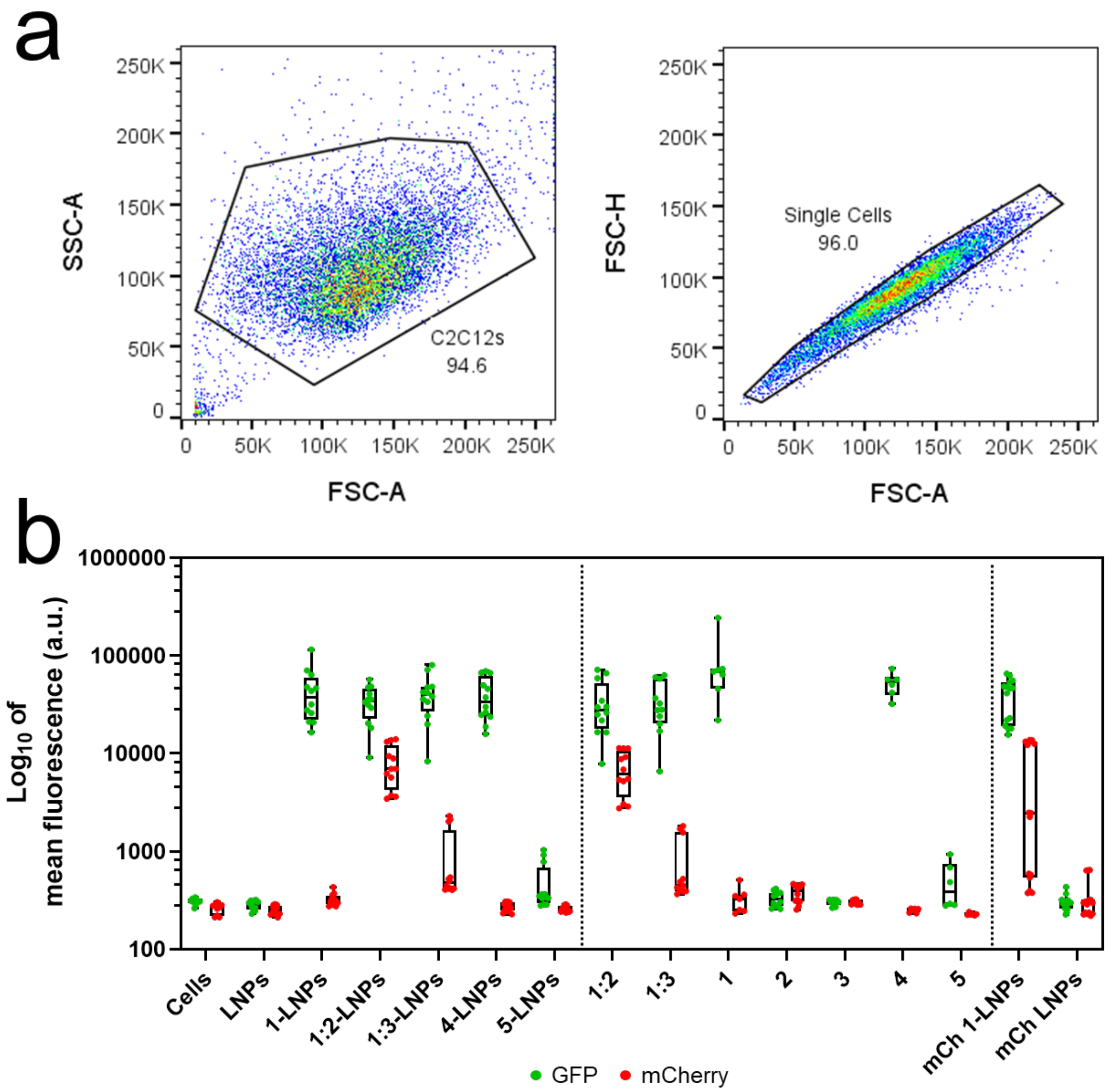

Figure S24. Flow cytometry data of C2C12 cells after exposure to control proteins. (a) Gating strategy on control C2C12 cells. **(b)** C2C12s were incubated with either LNPs (approximately 1.5 x 10^{10} vesicles, 100 µL) or proteins (1 µM, 100 µL) for 2 hours at 37 ºC. GFP fluorescence shown in green and mCherry fluorescence shown in red. Box and whiskers plots display min to max error bars and data points. Each data point is the mean of fluorescence of 10’000 single cell events.

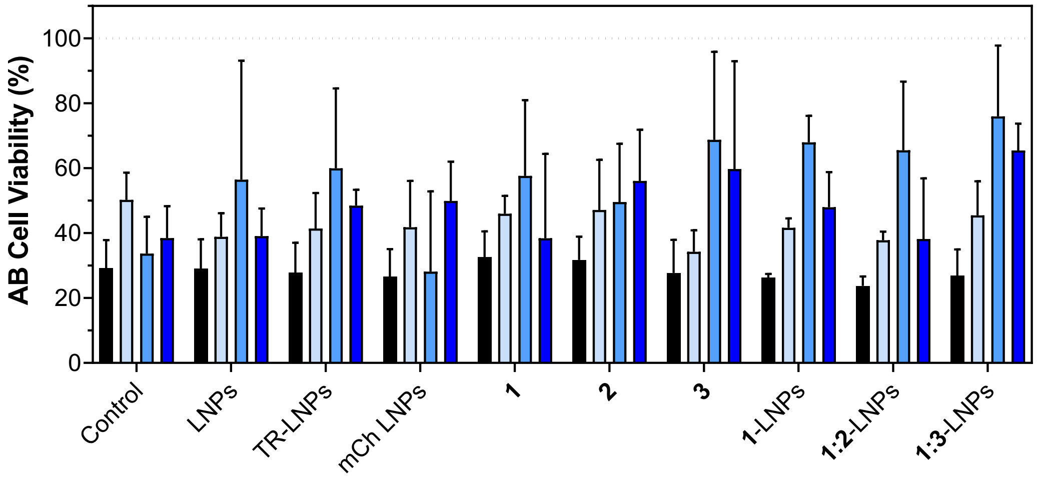

Figure S25. Cell viability of C2C12s (C3 mouse myoblasts) after exposure to different conditions. Cell viability was determined *via* alamarBlue® assay in which the cell metabolism reduction of resazurin to resorufin was measured. Data was normalised with respect to a standard curve. 50,000 cells per well were exposed to a range of conditions (20 µL of 1 µM proteins or approximately 1.5 x 10^{10} vesicles) for 2 hours with measurements taken immediately after this exposure (Day 0, shown in black), 24 hours post-exposure (Day 1, shown in light blue), and on Day 3 (shown in blue) and Day 7 (shown in navy blue). The mean and standard deviation are reported. The mean and standard deviation are reported.

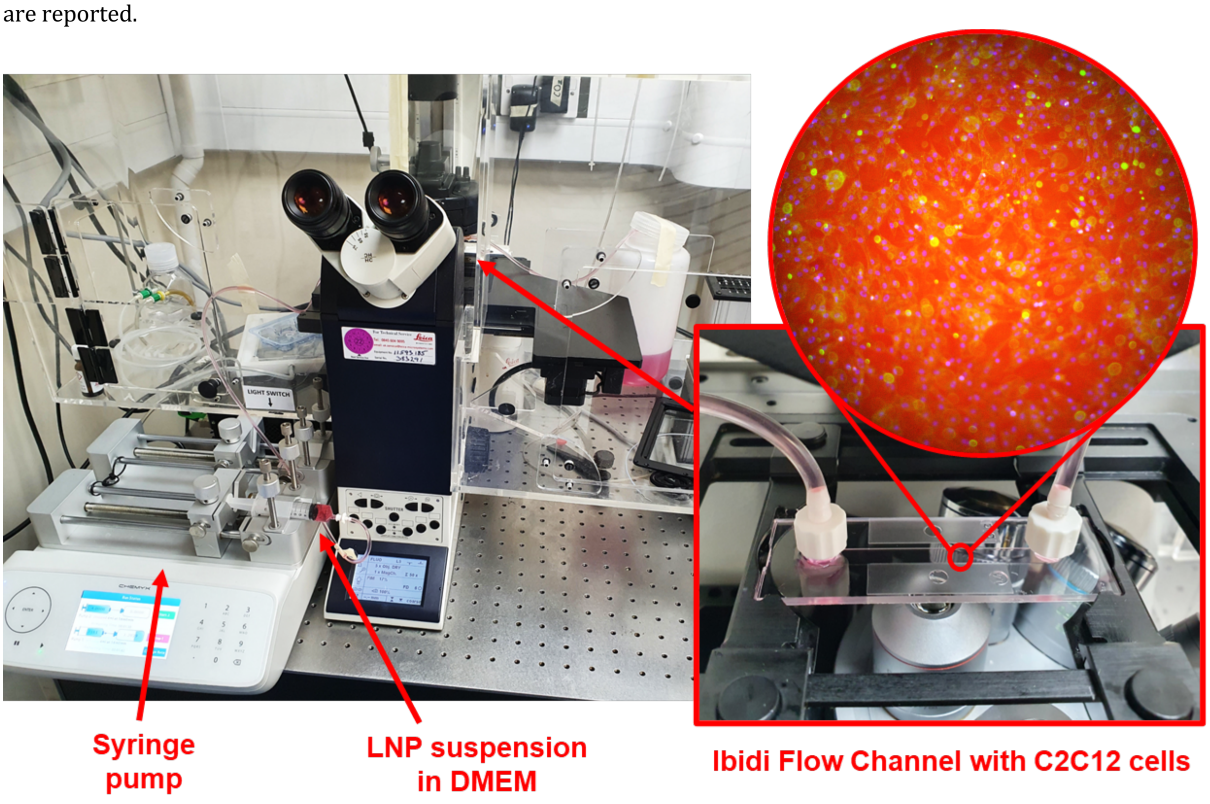

Figure S26. Set up of flow experiments. C2C12s were incubated in Ibidi Single Flow Chambers (250 µL effective volume, 0.4 mm channel height, 250'000 cells/slide) overnight at 37 ºC before the flow experiments. Flow channel was slotted on the widefield microscope, and connected to waste on the right, and to a dual syringe pump containing either DMEM media as wash solution, or the LNP suspension (approximately 1.5 x 10^{10} vesicles in 25 mL DMEM) on the left connexion. Flow rate was adjusted depending on the syringe volume to obtain a shear stress of 2 dynes in the chamber. Cells were washed with 10 mL of media before injecting 25 mL of the condition of interest, and were then washed with 30 mL DMEM to show retention of the constructs despite the shear stress. When flow was stopped in between syringe changes, **1:2**-LNP scattering could be observed as spheres, as shown in the zoom-in image.

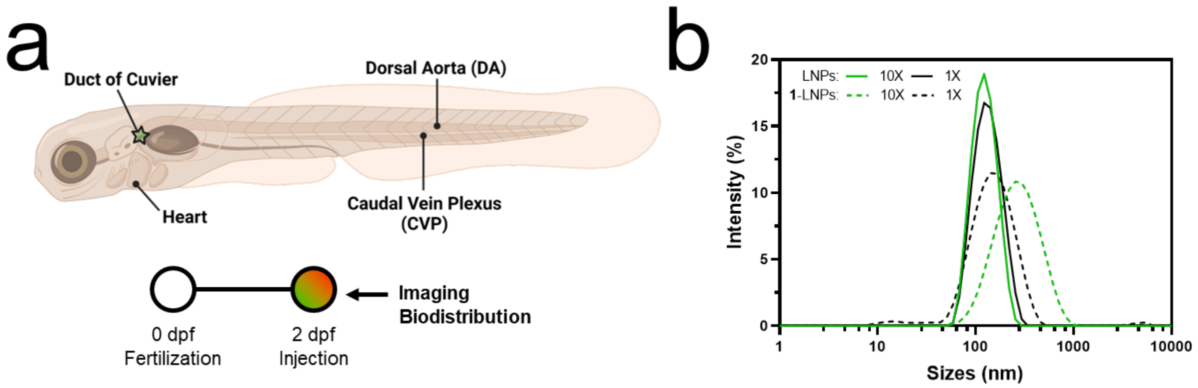

Figure S27. Zebrafish experiments. (a) Schematic of larval zebrafish anatomy relevant to the experiment. Image depicting larval zebrafish with organs and anatomical features noted. The systemic injection site at the Duct of Cuvier is noted with a green star. (b) Dynamic light scattering (DLS) showing the hydrodynamic diameter intensity of LNPs extruded as 10% (v/v) lipid film to buffer (shown in black) or as 100% (v/v) lipid film to buffer (shown in green), uncoated (shown as solid line) or coated with protein fusion **1** (shown as dotted line). All DLS measurements were performed in triplicate after equilibration at 25°C for 120 seconds. Average of the three measurements is plotted for each sample.

Supplementary Videos

Video S1. Live microscopy of C2C12 myoblasts (250'000 cells) in flow channel during the sample application of **1:2**-LNPs (approximately 1.5 x 10^{10} vesicles in 25 mL DMEM) for 15 minutes at 2 dynes shear stress. Nuclei are shown in blue, GFP is shown in green and mCherry in red. Images were taken every 15 seconds. Scale bar 200 μm.

Video S2. Live microscopy of C2C12 myoblasts (250'000 cells) in flow channel after sample application of **1:2**-LNPs for 15 minutes flowing DMEM (25 mL) at 2 dynes shear stress. Nuclei are shown in blue, GFP is shown in green and mCherry in red. Images were taken every 15 seconds. Scale bar 200 μm.

Video S3. Live microscopy of C2C12 myoblasts (250'000 cells) in flow channel during the sample application of **1:3**-LNPs (approximately 1.5 x 10^{10} vesicles in 25 mL DMEM) for 15 minutes at 2 dynes shear stress. Nuclei are shown in blue, GFP is shown in green and mCherry in red. Images were taken every 15 seconds. Scale bar 200 μm.

Video S4. Live microscopy of C2C12 myoblasts (250'000 cells) in flow channel after sample application of **1:3**-LNPs for 15 minutes flowing DMEM (25 mL) at 2 dynes shear stress. Images were taken every 15 seconds. Nuclei are shown in blue, GFP is shown in green and mCherry in red. Scale bar 200 μm.

Video S5. Live microscopy of the heart of a 2 day post-fertilisation larval zebrafish after injection of **1:2**-LNPs (2 nL, approximately 3 x 10^6 vesicles). GFP is shown in green and mCherry in red. Scale bar 50 μm.

Video S6. Live microscopy of the dorsal aorta of a 2 day post-fertilisation larval zebrafish after injection of **1:2**-LNPs (2 nL, approximately 3 x 10^6 vesicles). GFP is shown in green and mCherry in red. Scale bar 10 μm.

Video S7. Live microscopy of the caudal vein plexus of a 2 day post-fertilisation larval zebrafish after injection of **1:2**-LNPs (2 nL, approximately 3 x 10^6 vesicles). GFP is shown in green and mCherry in red. Scale bar 10 μm.

Video S8. Live microscopy of the heart of a 2 day post-fertilisation larval zebrafish after injection of **1:3**-LNPs (2 nL, approximately 3 x 10^6 vesicles). GFP is shown in green and mCherry in red. Scale bar 50 μm.

Video S9. Live microscopy of the dorsal aorta of a 2 day post-fertilisation larval zebrafish after injection of **1:3**-LNPs (2 nL, approximately 3 x 10^6 vesicles). GFP is shown in green and mCherry in red. Scale bar 10 μm.

Video S10. Live microscopy of the caudal vein plexus of a 2 day post-fertilisation larval zebrafish after injection of **1:3**-LNPs (2 nL, approximately 3 x 10^6 vesicles). GFP is shown in green and mCherry in red. Scale bar 10 μm.

Video S11. Live microscopy of the heart of a 2 day post-fertilisation larval zebrafish after injection of **1**-LNPs (2 nL, approximately 3 x 10^6 vesicles). GFP is shown in green and mCherry in red. Scale bar 50 μm.

Video S12. Live microscopy of the dorsal aorta of a 2 day post-fertilisation larval zebrafish after injection of **1**-LNPs (2 nL, approximately 3 x 10^6 vesicles). GFP is shown in green and mCherry in red. Scale bar 10 μm.

Video S13. Live microscopy of the caudal vein plexus of a 2 day post-fertilisation larval zebrafish after injection of **1**-LNPs (2 nL, approximately 3 x 10^6 vesicles). GFP is shown in green and mCherry in red. Scale bar 10 μm.

Video S14. Live microscopy of the heart of a 2 day post-fertilisation larval zebrafish after injection of protein fusion **2** (2 nL, 30 μM). GFP is shown in green and mCherry in red. Scale bar 50 μm.

Video S15. Live microscopy of the dorsal aorta of a 2 day post-fertilisation larval zebrafish after injection of protein fusion **2** (2 nL, 30 μM). GFP is shown in green and mCherry in red. Scale bar 10 μm.

Video S16. Live microscopy of the caudal vein plexus of a 2 day post-fertilisation larval zebrafish after injection of protein fusion **2** (2 nL, 30 μM). GFP is shown in green and mCherry in red. Scale bar 10 μm.

Video S17. Live microscopy of the heart of a 2 day post-fertilisation larval zebrafish after injection of protein fusion **3** (2 nL, 30 μM). GFP is shown in green and mCherry in red. Scale bar 50 μm.

Video S18. Live microscopy of the dorsal aorta of a 2 day post-fertilisation larval zebrafish after injection of protein fusion **3** (2 nL, 30 μM). GFP is shown in green and mCherry in red. Scale bar 10 μm.

Video S19. Live microscopy of the caudal vein plexus of a 2 day post-fertilisation larval zebrafish after injection of protein fusion **3** (2 nL, 30 μM). GFP is shown in green and mCherry in red. Scale bar 10 μm.
